# A multi-proxy approach to exploring *Homo sapiens*’ arrival, environments and adaptations in Southeast Asia

**DOI:** 10.1038/s41598-021-99931-4

**Published:** 2021-10-26

**Authors:** Anne-Marie Bacon, Nicolas Bourgon, Frido Welker, Enrico Cappellini, Denis Fiorillo, Olivier Tombret, Nguyen Thi Mai Huong, Nguyen Anh Tuan, Thongsa Sayavonkhamdy, Viengkeo Souksavatdy, Phonephanh Sichanthongtip, Pierre-Olivier Antoine, Philippe Duringer, Jean-Luc Ponche, Kira Westaway, Renaud Joannes-Boyau, Quentin Boesch, Eric Suzzoni, Sébastien Frangeul, Elise Patole-Edoumba, Alexandra Zachwieja, Laura Shackelford, Fabrice Demeter, Jean-Jacques Hublin, Élise Dufour

**Affiliations:** 1 UMR 8045 BABEL, CNRS, Université de Paris, Faculté de Chirurgie dentaire, 1 rue Maurice Arnoux, 92120 Montrouge, France; 2 Department of Human Evolution, Max Planck Institute for Evolutionary Anthropology, Leipzig, Germany; 3 Applied and Analytical Palaeontology, Institute of Geosciences, Johannes Gutenberg University, Mainz, Germany; 4 Section for Evolutionary Genomics, GLOBE Institute, University of Copenhagen, Copenhagen, Denmark; 5 UMR 7209 Archéozoologie, Archéobotanique: Sociétés, Pratiques, Environnements, Muséum National d’Histoire Naturelle, CNRS, Paris, France; 6 Anthropological and Palaeoenvironmental Department, Institute of Archaeology, Hoan Kiem District, Ha Noi, Vietnam; 7 Department of Heritage, Ministry of Information, Culture and Tourism, Vientiane, Laos; 8 Institut des Sciences de l’Évolution de Montpellier, Université de Montpellier, CNRS, IRD, EPHE, Montpellier, France; 9 Ecole et Observatoire des Sciences de la Terre (EOST Géologie), Institut de Physique du Globe de Strasbourg (IPGS) (CNRS/UMR 7516), Institut de Géologie, Université de Strasbourg, Strasbourg, France; 10 UMR 7362 Laboratoire Image Ville et Environnement, Institut de Géologie, Strasbourg, France; 11 Department of Earth and Environmental Sciences, Traps’ MQ Luminescence Dating Facility, Macquarie University, Sydney, Australia; 12 Geoarchaeology & Archaeometry Research Group, Southern Cross University, Lismore, Australia; 13 Institute of Vertebrate Paleontology and Paleoanthropology (IVPP) of the Chinese Academy of Sciences, Beijing, China; 14 Spitteurs Pan, Technical Cave Supervision and Exploration, La Chapelle-en-Vercors, France; 15 Muséum d’Histoire Naturelle, La Rochelle, France; 16 Department of Biomedical Sciences, University of Minnesota Medical School, Duluth, MN USA; 17 Department of Anthropology, University of Illinois at Urbana-Champaign, Urbana, IL USA; 18 Lundbeck Foundation GeoGenetics Centre, GLOBE Institute, Copenhagen, Denmark; 19 UMR 7206 Eco-Anthropologie, Muséum National d’Histoire Naturelle, CNRS, Paris, France; 20 Collège de France, Chaire de Paléoanthropologie, Paris, France

**Keywords:** Evolutionary ecology, Ecology, Evolution, Anthropology, Palaeontology

## Abstract

The capability of Pleistocene hominins to successfully adapt to different types of tropical forested environments has long been debated. In order to investigate environmental changes in Southeast Asia during a critical period for the turnover of hominin species, we analysed palaeoenvironmental proxies from five late Middle to Late Pleistocene faunas. Human teeth discoveries have been reported at Duoi U’Oi, Vietnam (70–60 ka) and Nam Lot, Laos (86–72 ka). However, the use of palaeoproteomics allowed us to discard the latter, and, to date, no human remains older than ~ 70 ka are documented in the area. Our findings indicate that tropical rainforests were highly sensitive to climatic changes over that period, with significant fluctuations of the canopy forests. Locally, large-bodied faunas were resilient to these fluctuations until the cooling period of the Marine Isotope Stage 4 (MIS 4; 74–59 ka) that transformed the overall biotope. Then, under strong selective pressures, populations with new phenotypic characteristics emerged while some other species disappeared**.** We argue that this climate-driven shift offered new foraging opportunities for hominins in a novel rainforest environment and was most likely a key factor in the settlement and dispersal of our species during MIS 4 in SE Asia.

## Introduction

Although the earliest forms of *Homo* occupied diverse C_3_-C_4_ environmental niches in Africa^1^, the genus is generally seen as primarily being adapted to open environments^2,3^. In Asia, early *Homo erectus* likely inhabited areas devoid of forests along river valleys in north China and Java and a niche partitioning between archaic humans and other large primates living in heavily forested habitats has been proposed^4–8^.

During the Late Pleistocene, the Far East witnessed a major turnover of hominins with the extinction of the last *H. erectus* in Indonesia^8^, the likely presence of the last Denisovans in several parts of the continent^9^ and eventually the replacement of all archaic groups following the arrival of *Homo sapiens*^10^. On a continental scale, it has been suggested that the shift from open habitats (mixed savannah and woodland) to rainforest habitats at the transition between the Middle Pleistocene and the Late Pleistocene triggered the decline of archaic hominins, unable to adapt to these new environments^11^.

Determining the palaeoenvironmental context facing different hominin species in Southeast (SE) Asia thus has the potential to feed into the debates relating to the uniqueness of our species. However, in the Pleistocene tropical Indochinese subregion, rare dental remains tentatively assigned to hominins have often been reinterpreted as remains of great apes (mainly orangutans of the *Pongo* genus)^5,12–14^, making it difficult to firmly associate hominins with records of past vegetation in many cases.

The dispersal route of *H. sapiens* towards southern China likely crossed Indochina^15^, but the timing of this event, its process —one or several waves possibly since ~ 100 thousand years ago (ka)^16,17^—and how *H. sapiens* adapted to rainforest environments remain unresolved. Certainly, the paucity of detailed chronology for several SE Asian sites contributes to obscuring our understanding of the period. To date, the earliest indisputable archaeological evidence of hominin adaptation to Asian tropical rainforests is actually quite recent and dated to ~ 73–63 ka in Lida Ajer, Sumatra^18^.

**Figure 1:**
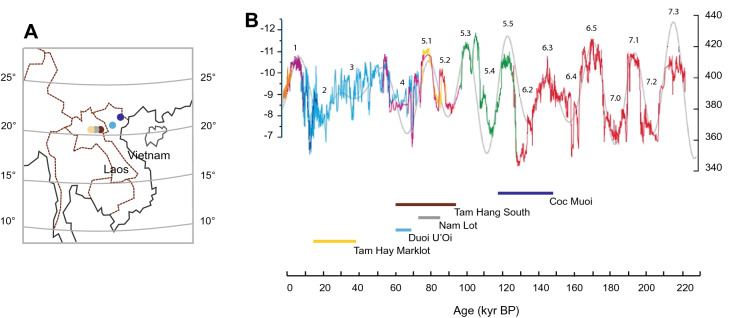
(**A**) Location of sites in northern Laos (Tam Hang South, Nam Lot, and Tam Hay Marklot) and northern Vietnam (Coc Muoi and Duoi U’Oi). (**B**) Sanbao, Dongee, and Hulu Chinese caves *δ*^18^O records showing millennial-scale climate shifts related to changes in East Asian summer monsoon intensity for the last 224 ka (modified after^87^). The decreases in *δ*^18^O values (‰, VPDB, left ordinate axis) correspond to increases in precipitation, i.e., the amount effect^77^. The right ordinate axis corresponds to the Northern Hemisphere summer insolation (65°N, W m^−2^). The age intervals of the faunas have been placed below the curve of *δ*^18^O records, from the oldest (right) to the youngest (left).

Here, we seek to try and address some of these crucial issues for the understanding of the evolution of our species by analysing five mammalian faunas from Vietnam and Laos, whose age ranges fall within different Marine Isotope Stages (MIS): Coc Muoi (148–117 ka, MIS 6–5), Tam Hang South (94–60 ka, MIS 5–4), Nam Lot (86–72 ka, MIS 5), Duoi U’Oi (70–60 ka, MIS 4) and Tam Hay Marklot (38.4–13.5 ka, MIS 3–2)^19–21^ (Fig. 1). From a palaeoecological point of view, the crown dimensions and stable isotopic measurements of identified taxa from these faunas are proxies for environmental reconstruction^7,11,20,22–24^ and a primary source of information on biotas occupied by hominins. In the area studied, the earliest occurrence of *H. sapiens* is documented by skeletal remains of several individuals from ~ 70 ka at Tam Pà Ling (~ 70–46 ka^25,26^) and by two teeth at Duoi U’Oi (70–60 ka^22^). However, an older putative hominin specimen associated with the Nam Lot assemblage (86–72 ka^22^) opens the possibility of an even earlier arrival^27^. We thus used palaeoproteomics^28–30^ to resolve the specific assignment of this specimen based on its dental enamel proteome, with the goal of better contextualizing the arrival of modern humans locally.

During the late Middle to the Late Pleistocene, local faunas were composed of a large proportion of modern taxa associated with a few archaic taxa^23,31–35^. Overall, the faunas were similar to those of other continents at the time as they were dominated by megaherbivores (> 1000 kg up to 5000 kg^36^), including elephant, stegodon, giant tapir, and several species of rhinoceroses and large bovids^37^. This similarity also extends to the local association of species with different ecologies and to their discrepancy with present-day spatial distributions (e.g., orangutans with pandas or tigers with hyenas). Most palaeontologists now consider that this unexpected association of species, named non-analogue faunas (^38–42^, but see Ref.^43^), results from the different responses of species to environmental changes that is, in an “individualistic manner”— according to their life-histories^44–47^.

Using classical zooarchaeological approaches, the analysis of SE Asian mammalian faunas for palaeoecological reconstructions has failed to detect major functional changes in mammalian communities^37,48,49^, with the palaeontological record appearing to be somewhat uniform. Indeed, in this tropical area, the species display broad ecological ranges, both latitudinal and altitudinal. Still, the question remains as to how species survived climatic shifts during the Pleistocene and adapted to non-analogue climates, exhibiting different sets of environmental variables (structure of the vegetation, rates of insolation, seasonality, amount of rainfall, etc.) than those of today^50,51^. Furthermore, the evolution of various lineages at the infraspecific level is generally unknown because movements of populations are seldom traceable in fossil records. An additional limitation of the studies of species dynamics in tropical Asia is the absence of preserved DNA in the fossil remains, which prevents the reconstruction of the genetic history of mammalian lineages. In Eurasia, most molecular analyses of ancient DNA (aDNA) focused on Beringian faunas, emphasising that the Late Pleistocene was a dynamic period for cold-adapted mammals influenced by climate changes^52–64^. These studies demonstrate various processes (*i.e.*, contraction of populations, local extinction, migration, replacement by new populations, or interspecies competition) resulting in the success of new and better-adapted populations over time^47,65–67^. In the meantime, the influence of climate cooling on warm-adapted populations remains largely unknown.

Using morphometric and particularly isotopic proxies from teeth, it is however possible to address various environmental issues and specifically to assess the effects of large climate oscillations on rainforest ecosystems at a regional scale and their impacts on the mammalian communities and associated hominins, as indicated by the growing body of research^20,22,24,68–73^.

Our dataset contains several hundreds of isolated teeth of mammals belonging to six mammalian Orders, *i.e.*, Artiodactyla, Perissodactyla, Proboscidea, Carnivora, Primates, and large Rodentia (Methods, Supplementary Materials and Methods, Supplementary Figs. S1–S3, Supplementary Tables S3–S4). All sites are located in a narrow latitudinal belt between 23° and 20° running through the northern regions of Laos and Vietnam (Fig. 1A). The location of the sites minimizes the variations in species body size due to abiotic parameters related to latitudinal distribution, *i.e.*, cline effect (temperature, distance from the coast, rain seasonality, amount of rainfall). However, the five sites are located at various altitudes, ranging from lowland sites at the level of the alluvial plain (Duoi U’Oi) to medium mountain sites (Nam Lot and Tam Hang South).

First, we compared carbon (*δ*^13^C_apatite_) and oxygen (*δ*^18^O) isotope measurements from dental enamel from a corpus of 335 specimens belonging to a large spectrum of taxa, using new data from Coc Muoi, Duoi U’Oi, and Tam Hang South, and those already published of Nam Lot and Tam Hay Marklot^20,22^. We estimated the *δ*^13^C_carbon source_ values in the diet of animals to specifically analyse the changes in proportions of C_3_-plants (trees, bushes, shrubs, and grasses) *versus* C_4_-plants (grasses, sedges) over the studied period^74^. The *δ*^13^C of bioapatite allows the reconstruction of palaeoenvironments based on these isotopically-distinct carbon sources. We also used the *δ*^13^C_carbon source_ to differentiate the C_3_ canopy forests from other C_3_ forested habitats to reveal local fluctuations of the tropical rainforests in relation to climatic changes^75,76^. *δ*^18^O values were used to provide additional palaeoecological information related to variation in abiotic conditions (latitude, climate, temperature, moisture content, amount, and isotopic composition of precipitation)^77–84^ (“Methods”).

Additionally, we used original morphometric data -the dental crown area of 213 specimens belonging to five taxa among herbivores and omnivores, the sambar deer (*Rusa unicolor*), the muntjac (*Muntiacus* sp.), the serow (*Capricornis sumatraensis*), the boar (*Sus scrofa*), and the macaque (*Macaca* sp.)- to detect significant phenotypic changes through time within their lineages. Combining the proxies based on stable isotope data with these morphometric data enabled us to identify which climate shifts had the most substantial impact on the mammalian communities in relation to rainforest dynamics.

Our fourth proxy takes into account the type of digestive physiology, using the ratio of ruminants *versus* hindgut fermenting herbivores by body mass category^42^, as an indicator of the expansion of open landscapes (primarily through the occurrence of exclusive grazing taxa) and therefore the contraction of rainforests.

Finally, we discuss how the climate changes that occurred during the Late Pleistocene might have influenced the adaptation of the first *H. sapiens* locally, and more widely in the SE Asian region. For that purpose, we used available climatic records, e.g., pollen data^85,86^, and Chinese caves *δ*^18^O data from speleothems^87^, as other relevant sources of information.

## Results

### Rejection of an early *Homo* species presence at Nam Lot

MS/MS spectra unambiguously assign the Nam Lot incisor (NL 433) to the genus *Pongo* (orangutans) with no unique and high-confidence matches to the genus *Homo*^29,30^. For those positions where we have proteomic data for the Nam Lot specimen, no sequence differences exist between *Pongo abelii* and *P. pygmaeus* in our reference sequences. As a result, we assign the specimen to the genus *Pongo* without further species specification (Supplementary Methods and Results).

### Stable isotope data

**Figure 2:**
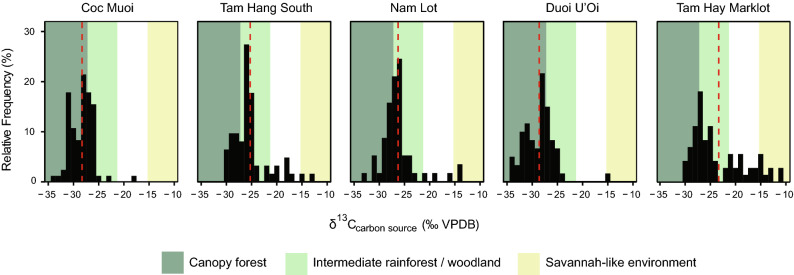
Histogram distribution of the relative frequency (%) in *δ*^13^C_carbon source_ values for all taxa in the five SE Asian faunas, following a chronological sequence from the oldest (left) to the youngest (right). Each bin represents a spacing of 1‰. Shaded areas represent *δ*^13^C_carbon source_ values associated with closed-canopy forests (*δ*^13^C_carbon source_ < −27.2‰); intermediate rainforests and woodland biomes (*δ*^13^C_carbon source_ >  − 27.2 ‰ and < − 21.3 ‰; and savannah-like environments (*δ*^13^C_carbon source_ > − 15.3‰). The white area (*δ*^13^C_carbon source_ > − 21.3 ‰ and < − 15.3 ‰) consists of values resulting from the combined consumption of both C_3_ and C_4_ resources, and does not correspond to any specific ecological environment. The dashed red line represents the mean *δ*^13^C_carbon_ _source_ value in each site.

**Figure 3:**
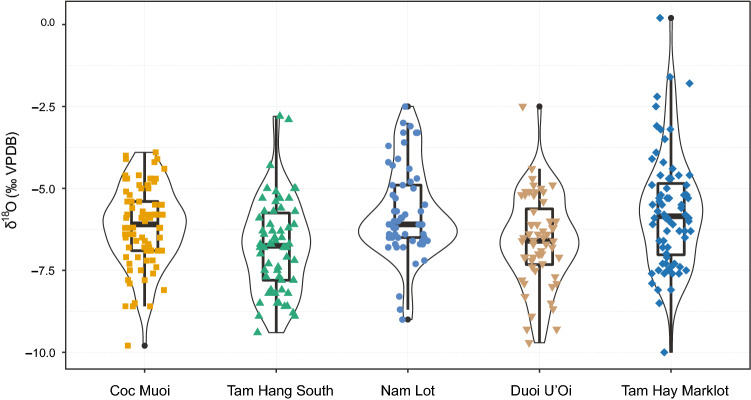
Distribution in *δ*^18^O values for all taxa in the five SE Asian faunas, following a chronological sequence from the oldest (left) to the youngest (right): Coc Muoi (), Tam Hang South (), Duoi U’Oi () and previously published data from Nam Lot () and Tam Hay Marklot (). The outline of the violin plots represents kernel probability density, where the width shows the proportion of the data found there. The boxes from the box and whisker plots inside the violin plots represent the 25th–75th percentiles, with the median as a bold horizontal line.

The *δ*^13^C_source_ and *δ*^18^O_apatite_ values of specimens belonging to all taxonomic groups are shown in Figs. 2 and 3 and Supplementary Tables S5–S7. The values (*δ*^13^C_apatite_, *δ*^13^C_carbon source_, and *δ*^18^O_apatite_) for all specimens and reference standards are presented in Supplementary Annexes S1–S2. With regards to the new data measured here, the *δ*^13^C_carbon source_ values for Coc Muoi, Duoi U’Oi and Tam Hang South range from − 33.8 to − 18.1 ‰ (average *δ*^13^C_carbon source_ = − 28.0 ± 2.4 ‰ (1 σ), n = 84), − 34.3 to − 15.1 ‰ (average *δ*^13^C_carbon source_ = − 28.4 ± 3.0 ‰ (1 σ), n = 60) and − 30.0 to − 12.0 ‰ (average *δ*^13^C_carbon source_ = − 25.0 ± 3.6 ‰ (1 σ), n = 62), respectively (Fig. 2).

The new *δ*^18^O_apatite_ values obtained for the three sites range from − 9.8 to − 3.9 ‰ (average *δ*^18^O_apatite_ = − 6.1 ± 1.2 ‰ (1 σ), n = 84), − 9.7 to − 2.5 ‰ (average *δ*^18^O_apatite_ = − 6.6 ± 1.3 ‰ (1 σ), n = 60) and − 9.4 to − 2.8 ‰ (average *δ*^18^O_apatite_ = − 6.7 ± 1.4 ‰ (1 σ), n = 62), respectively for Coc Muoi, Duoi U’Oi and Tam Hang South (Fig. 3).

Statistically significant differences between sites, from both the novel (Coc Muoi, Tam Hang South, and Duoi U’Oi) and published data (Nam Lot and Tam Hay Marklot), were determined through Kruskal–Wallis one-way analysis of variance for *δ*^13^C_carbon source_ (H(4) = 83.3, *p-value* < 2.2e−16) and for *δ*^18^O_apatite_ (H(4) = 25.5, *p-value* = 4.019e−05). Post-hoc Dunn’s test pair-wise comparisons draw out the sites from Vietnam as distinct from the sites in Laos regarding their *δ*^13^C_carbon source_ values. Tam Hay Marklot and Nam Lot also appear to be significantly different from each other. Finally, *δ*^18^O_apatite_ values from Tam Hang South are identified as significantly different from those of all other sites except Duoi U’Oi, while Duoi U’Oi itself is being drawn out as significantly different to Nam Lot and Tam Hay Marklot (Supplementary Tables S8, S9).

Broadly, the ranges and medians of *δ*^18^O values fluctuated (Fig. 3), in accordance with *δ*^13^C_carbon source_ values (Fig. 2). However, the distribution of the *δ*^13^C_carbon source_ values highlights that the C_3_ forested environments (canopy forests, intermediate rainforests and woodlands) remained predominant over the period studied. Furthermore, when we look at the percentages of specimens according to the distribution of *δ*^13^C_carbon source_ values associated to the different biomes in Table 1, the data demonstrate that environmental conditions changed significantly through the Coc Muoi—Tam Hay Marklot temporal series. Tropical forests were thus apparently sensitive to climate change. Our results particularly illustrate the dynamics of the canopy forests (*δ*^13^C_carbon source_ <  − 27.2 ‰), and show their potential for contraction across space and time: Coc Muoi (65.4%), Tam Hang South (27.4%), Nam Lot (42.1%), Duoi U’Oi (73.3%), and Tam Hay Marklot (26.3%).

**Table 1:** Percentage (%) and number of specimens (n/N) in the five faunas according to the distribution of *δ*^13^C_carbon source_ values (‰ VPDB) from the oldest (top) to the youngest (bottom).

Sites	*δ*^13^C_carbon source_ < − 27.2 ‰		*δ*^13^C_carbon source_ > − 27.2 ‰ and < − 21.3 ‰		*δ*^13^C_carbon source_ > − 21.3 ‰ and < − 15.3 ‰		*δ*^13^C_carbon source_ > − 15.3‰	
Coc Muoi	65.4%	55/84	33.3%	28/84	1.1%	1/84	–	0/84
Tam Hang South	27.4%	17/62	58.0%	36/62	11.2%	7/62	3.2%	2/62
Nam Lot	42.1%	24/57	49.1%	28/57	5.2%	3/57	3.5%	2/57
Duoi U’Oi	73.3%	44/60	25.0%	15/60	–	0/60	1.6%	1/60
Tam Hay Marklot	26.3%	19/72	43.0%	31/72	18.0%	13/72	12.5%	9/72

Closed-canopy forests (*δ*^13^C_carbon source_ <  − 27.2 ‰); intermediate rainforests and woodland biomes (*δ*^13^C_carbon source_ >  − 27.2 ‰ and < − 21.3 ‰); no specific ecological environment (*δ*^13^C_carbon source_ > − 21.3 ‰ and < − 15.3 ‰); and savannah-like environments (*δ*^13^C_carbon source_ > − 15.3‰).

### Distribution of herbivore species by body mass and digestive strategy

**Figure 4:**
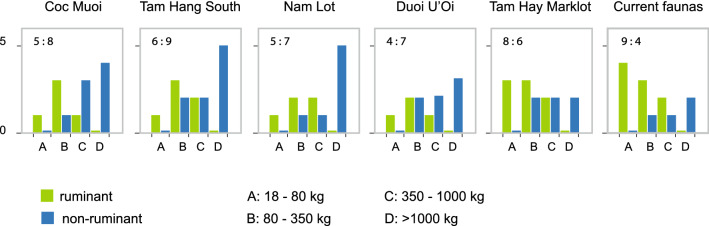
Number of species by body mass category and digestive strategy in the five faunas, following a chronological sequence from the oldest (left) to the youngest (right). The ratio refers to the number of ruminants *versus* non-ruminant taxa. See Supplementary Table S10 for the list of taxa within each body mass category.

The sequence of the faunas by body mass and digestive strategy is presented in Fig. 4 and Supplementary Table S10. In the three oldest faunas, hindgut fermenting taxa, *i.e.*, non-ruminant taxa, including seven large herbivores (> 350 kg) and megaherbivores (> 1000 kg) belonging to the following genera, *Megatapirus*, *Tapirus*, *Stegodon*, *Elephas*, *Rhinoceros,* and *Dicerorhinus* (*vs.* only one ruminant *Bos* species), dominated the biomass. Duoi U’Oi with a ratio “ruminant *vs.* non-ruminant taxa” of 4:7 shows a change in the composition of megaherbivores with the absence of *Megatapirus* (> 350 kg) and *Stegodon* (> 1000 kg). However, hindgut fermenting herbivores remain predominant since the loss in the diversity of large-bodied archaic taxa is not compensated by an increase in ruminants. Tam Hay Marklot marks a shift that represents small- to medium-sized ruminants (18 to 350 kg) (among which *Rucervus eldii*, *Axis porcinus* and *Naemorhedus caudatus*) becoming predominant (ratio ruminant *vs.* non-ruminant taxa of 8:6). This trend apparently continued to the present, as seen in the increase of grazing species in current faunas at these latitudes (ratio 9:4) (Fig. 4).

### Crown area dimensions as a phenotypic signal

**Figure 5:**
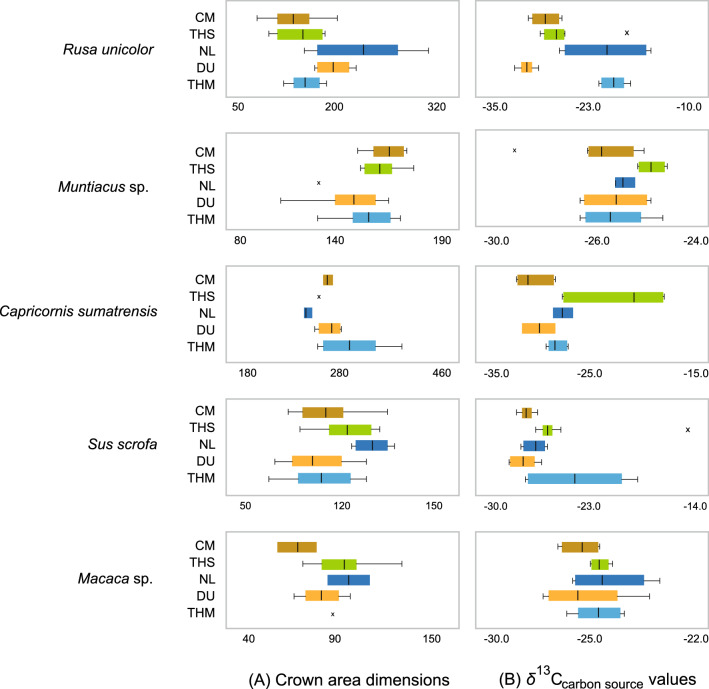
Distribution of crown area dimensions (**A**) and *δ*^13^C_carbon source_ values (**B**) in five taxa (the boxes represent the 25th–75th percentiles, median and whisker plots), following a chronological sequence from the oldest (top) to the youngest (bottom). *CM* Coc Muoi, *THS* Tam Hang South, *NL* Nam Lot, *DU* Duoi U’Oi, *THM* Tam Hay Marklot. See Supplementary Table S13 for the number of specimens.

The dimensional ranges of crown areas of p3 (*Rusa unicolor* and *Sus scrofa)* and m3 (*Macaca* sp., *Muntiacus* sp., and *Capricornis sumatraensis*) differ between sites (Fig. 5A). However, statistical analyses were limited by unbalanced sample size for some sites, and only *R. unicolor* (n = 65) and *S. scrofa* (n = 61) were analysed with the Kruskal–Wallis test (H(4) = 21.09, *p-value* = 0.0003 and H(4) = 14.25, *p-value* = 0.007, respectively). Post-hoc Dunn’s test pair-wise comparisons draw out *R. unicolor* from Coc Muoi as significantly differing from those of Nam Lot (*p-value* < 0.005) and Duoi U’Oi (*p-value* < 0.05); and *R. unicolor* of Nam Lot differing from that of Tam Hay Marklot (p < 0.05). *S. scrofa* samples also show significant differences between populations (*p-value* < 0.05): Coc Muoi *vs.* Nam Lot; Tam Hang South *vs.* Duoi U’Oi; Nam Lot *vs.* Duoi U’Oi and Tam Hay Marklot (Supplementary Tables S11 and S12).

Overall, there is an appearance of cumulative effects from Coc Muoi to Nam Lot, whereby populations follow a directional evolution towards either a greater (*R. unicolor*, *S. scrofa*, and *Macaca* sp.) or a smaller (*Muntiacus* sp. and *C. sumatraensis*) crown area surface according to the taxon. In the overall faunal sequence, Duoi U’Oi marks a shift with a change in this directional evolution. This shift is particularly notable in *S. scrofa*, but the five taxa studied seemingly appear affected by this reversal of dimensions in lineages (Fig. 5A). This reversal is used here as a signal indicating that populations with new phenotypic characteristics emerged, either due to adaptation or replacement of populations (through extinction or assimilation), in the face of high selective pressures. Therefore, in the Coc Muoi—Tam Hay Marklot temporal series, Duoi U’Oi seems to mark some kind of turnover in populations.

## Discussion

From the available record discussed here, no clear human presence in the area prior to ~ 70 ka can be demonstrated. However, the ability to obtain protein sequence information from tropical areas^30^ and to distinguish between *Pongo* and *Homo,* as shown by the results of our palaeoproteomic analysis of the Nam Lot incisor (86–72 ka), opens up the possibility to directly address the question of early *H. sapiens* presence in SE Asia in the future.

**Figure 6:**
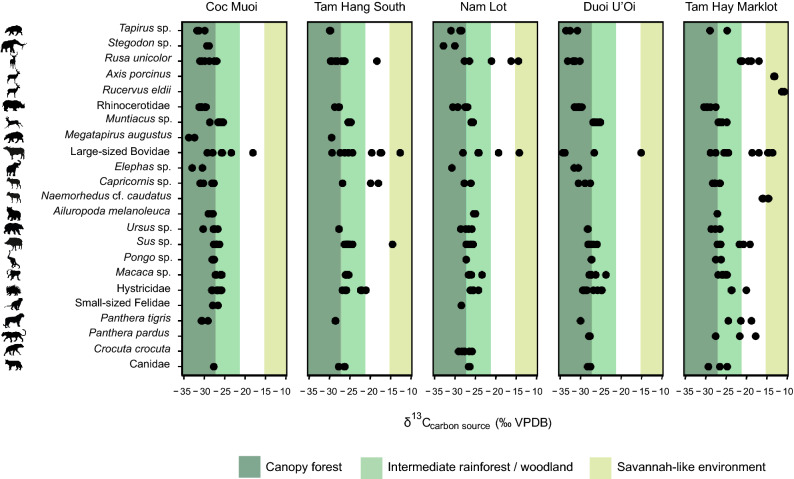
Comparison of range of *δ*^13^C values in selected taxa in the five faunas, following a chronological sequence from the oldest (left) to the youngest (right). Shaded areas represent *δ*^13^C_carbon source_ values associated with closed-canopy forests (*δ*^13^C_carbon source_ <  − 27.2 ‰), intermediate rainforests and woodland biomes (*δ*^13^C_carbon source_ >  − 27.2 ‰ and < − 21.3 ‰); and savannah-like environments (*δ*^13^C_carbon source_ > − 15.3 ‰). The white area (*δ*^13^C_carbon source_ > − 21.3 ‰ and < − 15.3 ‰) consists in values resulting from the combined consumption of both C_3_ and C_4_ resources, and does not correspond to any specific ecological environment.

The relative similarity in *δ*^13^C_carbon source_ and *δ*^18^O values between Coc Muoi (148–117 ka) and Duoi U’Oi (70–60 ka) suggests that climatic conditions induced a C_3_-dominated ecosystem in two distinct periods. As shown in the curves of the Sanbao/Hulu *δ*^18^O Chinese caves records^87^ in Fig. 1B, and considering the age ranges of the faunas, the predominance of these forested ecosystems could be associated with two high-amplitude drops in monsoon intensity, during MIS 6 for Coc Muoi (MIS 6.2^88,89^) and during MIS 4 for Duoi U’Oi (Fig. 2 in^87^). In Coc Muoi and Duoi U’Oi, closed rainforests contained most of the mammalian biomass composed primarily of browsers weighing up to ~ 5000 kg (Fig. 4 and Supplementary Table S10). However, on closer inspection, the two sites reveal marked differences in the species relying on canopy forests for their diet (Fig. 6). Firstly, Duoi U’Oi marks a decline in the diversity of megaherbivores with the absence of two archaic taxa: the giant tapir *Megatapirus augustus* and the proboscidean *Stegodon orientalis*. Both sites are situated in the same vegetation zone < 400 m above sea level (asl), and other sources of variability are reduced, supporting the hypothesis of a predominant climatic effect on mammalian communities.

Secondly, the results of *δ*^13^C_carbon source_ values demonstrate that, at Duoi U’Oi, the environmental changes induced a redistribution of ecological niches with new species interactions. The difference with the older Tam Hang South and Nam Lot faunas is notable, and the MIS 4 Duoi U’Oi fauna appears significantly different from both MIS 5 faunas (Fig. 6 and Supplementary Table S8). In particular, the sambar deer *R. unicolor*, the tapir *Tapirus sp.,* and the large-sized bovids foraged in this novel C_3_-dominated ecosystem of Duoi U’Oi. Furthermore, the Duoi U’Oi *δ*^18^O values, globally lower than those that prevailed in Nam Lot, suggest the arise of colder and/or wetter conditions (Fig. 3).

While forested environments dominated over the period, some faunas also comprised species whose diet relied on mixed C_3_–C_4_ and/or C_4_ resources (Figs. 2 and 6). This is particularly the case of the Tam Hang South and Nam Lot faunas, with *δ*^13^C_carbon source_ values showing the presence of more open environments in conjunction with the reduction of forested habitats: 11.2% (C_3_–C_4_) and 3.2% (C_4_) for Tam Hang South and 5.2% (C_3_–C_4_) and 3.5% (C_4_) for Nam Lot (Table 1). These data have been associated with increased seasonality^24^, and the data of Zheng and Lei (pollen records from the nearby Leizhou Peninsula of southern China, at the latitudes 21°–20° and altitude < 260 m^85^) indicate that mountainous slopes were covered by monsoon evergreen forests, with a dense shrub layer. Tam Hang South and Nam Lot also show different sets of environmental variables with marked differences of *δ*^18^O values between each other (but not due to altitudinal variations since they are about 150 m apart and located at the same elevation ~ 1120 m), suggesting, despite the lack of age precision, how dynamic the interglacial MIS 5 was.

In the second half of the Late Pleistocene, for which the mammalian faunas are not documented in the studied area, the landscape remained highly forested and humid between ~ 70 and ~ 32 ka, as indicated by the carbon and oxygen isotope records from the terrestrial snail *Camaena massiei* in the nearby Tam Pà Ling site in Laos^90^. A notable change towards more open landscapes associated with increased aridity, as a result of low sea level and expansion of land surface on the continent^91^**,** is later illustrated by our *δ*^13^C_carbon source_ values of Tam Hay Marklot (38.4–13.5 ka^20^) (Fig. 6). Further west, Tham Lod rockshelter in Thailand (33–11.5 ka^70,92^) shows similar conditions: the mixed-feeders, sambar deer and large bovids, seemingly moved to grassland areas and the grazers, medium-sized cervids (*Axis*, *Rucervus*) and small bovids (*Naemorhedus*), occupied new spaces, most likely coming from southern latitudes where subpopulations had, over the course of time, become adapted to living in open landscapes^93^ (Supplementary Figs. S5 and S6, Supplementary Annex S4). The sites of Boh Dambang (25–18 ka, Cambodia^94^), further south in the heart of the postulated savannah corridor^91^, and Tam Hay Marklot show nevertheless that the peninsula remained covered by patches of closed-canopy forests inhabited by browsers (*Muntiacus*, *Rhinoceros*, and *Tapirus*), even at the height of the aridity around the Last Glacial Maximum (LGM; 26.5–19 ka)^95^. Indeed, 26.3% of *δ*^13^C_carbon source_ values at Tam Hay Marklot and 9.6% at Boh Dambang are indicative of such environments (Supplementary Table S14), with results obtained from quite comparable sampled taxonomic groups in both sites (Supplementary Table S7). The contraction of the forested habitat significantly altered the carrying capacity of ecosystems, forcing large predators to seek new hunting opportunities among savannah-dwelling species, either for tigers (*Panthera tigris*), leopards (*P. pardus*), or hyenas (*Crocuta crocuta*) (Fig. 6 and Supplementary Fig. S5).

The present analysis combining crown area dimensions (Fig. 5A) with *δ*^13^C_carbon source_ data (Fig. 5B) reveals a major turnover of populations in the mammalian community of Duoi U’Oi correlated with the return of a C_3_-dominated landscape demonstrated by the drop in carbon isotope values. At Duoi U’Oi, in contrast to the older sites, populations with new phenotypic traits emerged, better adapted to this novel environment. This body of evidence may be interpreted as an adaptive response to a major selective pressure. The fact that species of different body weight ranging from ~ 15 kg (muntjac) to ~ 220 kg (sambar deer), of different dietary strategies (omnivores *vs.* herbivores), and adapted to different niches (ground-dwelling ungulates *vs.* arboreal monkeys), experienced similar evolutionary trends indicates that the entire ecosystem was impacted. As shown in Fig. 1B, the curves of the Sanbao/Hulu *δ*^18^O caves records^87^ reveal that the period witnessed a short-term climatic change with a violent and rapid drop of monsoon intensity at the onset of MIS 4 (an event of comparable amplitude than that which occurred during LGM). This climatic event likely resulted into strong selection pressures that triggered new adaptations and movements of populations. The abruptness of this climate transition with a duration of approximately < 300 years^96^, and a drop of mean temperatures of around 5–6 °C^85^, is the most likely explanation for the turnover of populations and the disappearance of the last archaic species (*Stegodon* and *Megatapirus*). To date, from the studied faunal records, there is no evidence that these species were associated with *H. sapiens*. Growing evidence based on phylogeographic analyses (aDNA) show that within-species populations replacements, either due to interactions between populations or due to effects of abrupt climatic changes, could be rapid processes leading to major extinction and recolonization events^53,54^.

In southern China, the late Middle to early Late Pleistocene series from Yugong, Quzai, and Baxian displays the same trend, as shown by the pattern of the *δ*^13^C values in *R. unicolor* and *S. scrofa*^24,35,73^ (Supplementary Figs. S7 and S8, Supplementary Annex S3). Therefore, the cooling event might have led canopy forest-dwelling populations from south China to expand their range in lower latitudes.

From a palaeoecological point of view, our findings confirm that a rainforest ecosystem prevailed at the end of the late Middle Pleistocene at these latitudes (Supplementary Fig. S7). They are consistent with the environmental reconstruction made by Louys and Roberts^11^. Our findings also question the relationship between the changes in the rainforest environment and the major turnover of earlier hominins (*H. erectus*, Denisovans) in SE Asia before the arrival of *H. sapiens.* Indeed, in the assumption that archaic hominins were not able to adapt to a rainforest habitat^5,11,17^, an environment like that of Coc Muoi could have been an obstacle for their local settlement.

Not only did the environmental changes impact the faunas as shown in our study, but also potentially *H. sapiens*. Indeed, within this broad record of forest persistence in the Late Pleistocene, we do notice important changes at the time of its first known occurrence in the area. Humans are documented by two heavily worn teeth (therefore assigned cautiously to hominins or *Homo* sp.) at Duoi U’Oi (70–60 ka^22^), and *H. sapiens* was present ~ 200 km away at the Tam Pà Ling cave site at that time (~ 70–46 ka^25,26^). From the available record, it is clear that humans who settled in this area during MIS 4, ca. 70–50 ka^97^, had to cope with heavily forested environments^90^. However, the relative cooling period of MIS 4 may have caused a profound transformation of the composition and structure of the forest, as demonstrated further East at the same latitude by the pollen records analysis (at an altitude < 260 m) by Zheng and Lei^85^. Indeed, owing to the lowering of the upland vegetation zone, the monsoon evergreen forests changed into montane forests with more temperate plants and a notable increase in conifers, previously rare trees, and ferns (20%). Based on palynological records in a comparable altitudinal zone (< 212 m) from the south Chinese caves in Chongzuo, Li et al.^86^ highlighted similar changes during the Late Pleistocene with the occurrence of subtropical mixed broadleaved-coniferous forests.

The palynological analysis of Duoi U’Oi, although based on few elements, likewise indicates a relatively high frequency of fern spores (25%) and non-arboreal taxa (31%), but also a low representation of mangrove pollen grains (2%)^22^. In the absence of archaeological evidence (lithic or organic industry, bones with butchery marks, etc.), many aspects of the human foraging behaviour remain challenging to assess in this part of SE Asia at this time. However, the different types of tropical rainforests provide edible plants, fruits, seeds, nuts and honey^98^, and the rivers would have offered predictable resources of shells, fishes, molluscs, and algae. The Duoi U’Oi biota supported a wide spectrum of game, despite the loss of archaic megaherbivores. The presence of humans at Duoi U’Oi is not only documented by two isolated teeth associated with the faunal assemblage, but also by indirect evidence. Indeed, the mortality profile of the sambar deer at the site clearly suggests an anthropic signature^19^. At Duoi U’Oi, humans were able to pursue and tackle their prey deep into the rainforest and selectively hunted mature adult individuals. Likely, the novel type of vegetation that transformed the shrub, fern, and herb strata during MIS 4 rendered the forest easier to enter and to navigate, offering new hunting opportunities for foragers. Humans apparently managed to successfully adapt in the prevailing habitat of closed-canopy forests^18,99–101^, most likely well before developing specialized foraging behaviour, with particularly a greater proportion of arboreal and semi-arboreal species *vs.* terrestrial species among hunted prey ~ 45 ka^99,102–104^.

Owing to the climate dynamics of the Late Pleistocene, our findings support a successful wave of dispersal of our species into the region during MIS 4^97,105–107^. A combination of ecological and behavioural factors seems to have helped early *H. sapiens* to successfully respond to the challenge of a rainforest environment: the turnover of the vegetation allowing humans to occupy a new ecological niche at the onset of MIS 4^108^ and the capacity of our species to adapt efficiently to this environment^109^. In the light of recent re-assessments of *H. sapiens* dispersal in the far East ca. 65 ka to 45 ka, although highly debated^97,110,111^, our species likely entered into south China through a similar type of rainforest, as also shown by the similarities in the isotopic data between Duoi U’Oi and Baxian^24,35^ (Supplementary Fig. S7). There are no available comparable isotopic data for the SE Asian mainland in the mid-Late Pleistocene, and it remains to demonstrate the habitats *H. sapiens* expanded through as it moved towards Island SE Asia during this period. However, we know that, concomitantly to this climate-driven turnover of the vegetation, modern humans reached Sumatra by 73–63 ka where they occupied a dense evergreen rainforest ecosystem^18^. From 46 ka, at Niah cave in Borneo, modern humans efficiently exploited the tropical environments, using sophisticated hunting technologies and being able to process toxic plants for consumption^103^. Interestingly, the climatic shift during MIS 4 has been considered as the primary driver of the migration of humans out of Africa towards Eurasia, due to cooler conditions and lower sea level^112–114^, allowing the populations to reach northern Australia possibly by 65 ka^115,116^.

## Conclusion

The study of the evolution of past SE Asian ecosystems is restricted by a paucity of palaeontological records, atypical conservation of remains, discontinuity, and available age precision of faunas, which limit our understanding of warm-adapted species responses to climate changes. Our multi-proxy approach, combining morphometric and isotopic data along with the type of digestive physiology, has nonetheless revealed that the cooling event of MIS 4 likely deeply affected the overall biotope of the region at the time of human arrival, leading to a turnover in mammalian populations associated with a densely forested landscape. Our findings suggest that the novel composition and structure of the rainforest was most likely the key factor facilitating the rapid dispersal of *H. sapiens* into SE Asia.

Furthermore, our analysis highlights the importance of palaeoproteomics to clarify the taxonomic assignment of remains (hominins *vs.* pongines). Indeed, given the scarcity of evidence of early *H. sapiens* in the region, this method will be essential for further contextualizing the arrival and dispersal of our species on the continent.

## Methods

### Geographical context

The sites are located at different altitudes: 113 m above sea level (asl) (Duoi U’Oi, Vietnam), 361 m asl (Coc Muoi, Vietnam), 1120 m asl (Tam Hang South/Nam Lot, Laos), and 809 m asl (Tam Hay Marklot, Laos). They are located 120 km (Duoi U’Oi), 170 km (Coc Muoi), and 270 km (Laotian sites) from the coast bordering the Gulf of Tonkin (Supplementary Fig. S1). Today, this zone is characterized by a humid subtropical climate defined by hot and humid summers and cold and mild winters according to the Köppen-Geiger climate classification. Local climate data, average annual temperature, and average annual rainfall are: 23.7 °C and 1735 mm (Hoà Binh province, Duoi U’Oi, Vietnam); 21.9 °C and 1349 mm (Lang Son province, Coc Muoi, Vietnam); 19.8 °C and 1331 mm (Houaphan province, upland region of Xamneua, Nam Lot, Tam Hang South, and Tam Hay Marklot, Laos) (https://en.climate-data.org/).

### Palaeontological and chronological data

The sites were excavated between 2003 and 2015. The description of sites and palaeontological contents can be found elsewhere in publications^19–22^ and in a condensed version in Supplementary data. All assemblages present similar preservation of remains due to taphonomic and geological processes of deposition in the karsts. They consist mainly of isolated teeth of mammals and, due to preservation bias, only species >  ~ 5 g constitute the assemblages: Coc Muoi (NISP = 1323), Tam Hang South (NISP = 673), Nam Lot I (385 teeth) and Nam Lot II (5 teeth) (NISP = 390), Duoi U’Oi (NISP = 871) and Tam Hay Marklot (NISP = 1364) (Supplementary Tables S3 and S4). The taphonomic analysis of the sites reveals that the porcupines were the main accumulator agent of bones of large mammals (most of the teeth are gnawed) before burial in the sediments. All remains deposited at the sites have been transported throughout the karstic network by waters that led to the loss of the smallest elements^19^**.** Based on observations in the wild, Brain^117^ showed that remains collected by porcupines give a good representation of the number of carcasses left at a site or surrounding a site, and therefore is a good representation of the abundance of species. Thus, the assemblages are constituted of similar taxonomic groups. Almost all groups could be sampled for the stable carbon and oxygen isotope analyses (except for the Proboscidea from Tam Hang South and Tam Hay Marklot), allowing to compare communities through time (Supplementary Table S7).

The five faunas do not represent a continuous record as two major gaps are present, between Coc Muoi (148–117 ka) and Tam Hang (92–60 ka) and between Duoi U’Oi (70–60 ka) and Marklot (38.4–13.5 ka) (Fig. 1 and Supplementary Figs. S2 and S3). No artefacts or other objects (charcoal, ornaments, traps, etc.) have been found in association with the faunal remains.

### Crown area dimensions

In this analysis, we used the crown area dimensions (maximum length x maximum width) of teeth as an indicator of ecological changes in five lineages of mammals (*Muntiacus* sp., *Capricornis sumatraensis*, *Rusa unicolor*, *Sus scrofa*, and *Macaca* sp.). We chose these taxa, defined at the species or the genus level, because they are common to all five faunas and documented by a sufficient number of specimens (Supplementary Tables S5 and S6 and Annex S6). However, such analysis is constrained by numerous biases including the differential representation of tooth types within a given taxon. That is why we selected left and right m3s in *Muntiacus* sp., *C. sumatraensis* and *Macaca* sp. Concerning the two other taxa, several tooth types were represented in significant numbers, p3, p4 and m3 in *S. scrofa*, and p3 and p4 in *R. unicolor*, but had different ranges of variation. In *R. unicolor*, p4s have similar ranges of variation between sites, unlike p3s. In *S. scrofa*, m3s showed a greater variability with large overlaps between sites. Considering the purpose of our study the use of crown area dimensions as a signal of a new phenotype p3 was the most useful tooth type in both taxa. This is most likely due to selective adaptive pressures on skulls. In suids, for example, populations differ in skull, palate, and tooth row lengths^118^. We have not attempted to estimate the body mass of individuals.

### Stable carbon and oxygen isotope data

Stable carbon isotopes of bioapatite (*δ*^13^C_apatite_) reflect the relative proportion in a consumer’s diet of ingested carbon derived from a food web’s primary sources, namely plants using either C_3_ or C_4_ photosynthetic pathways^74^. In tropical and subtropical regions, more humid forest and woodland habitats are associated with C_3_ plants that exhibit low *δ*^13^C values, whereas drier and more open environments are characterized by C_4_ plants with high *δ*^13^C values^76,119,120^. Additionally, the lowest *δ*^13^C values reflect densely forested conditions resulting from a “canopy effect” and can thus be used to differentiate C_3_ forested environments. Finally, using measured *δ*^13^C_apatite_ values and body mass-adapted enrichment factors (Supplementary Materials and Methods), we estimated the initial *δ*^13^C value of the carbon source in the animal’s diet, herein labelled as “*δ*^13^C_carbon source_”.

Stable oxygen isotopes (*δ*^18^O values) were used to provide additional palaeoecological information. The primary source of variation in *δ*^18^O of enamel is the oxygen isotopic composition of drinking water and chemically-bound water in diet (*i.e.*, water found in plants)^78–83^. This water is itself controlled by various environmental and geographic conditions such as latitude, climate, temperature, moisture content, amount and isotopic composition of precipitation (at low latitudes, the variation of *δ*^18^O rainfalls is mainly influenced by the amount of precipitation, *i.e.*, amount effect)^77,78,84^.

Fossil teeth allocated to the mammalian Orders (Artiodactyla, Perissodactyla, Proboscidea, Carnivora, Primates, and Rodentia) from Coc Muoi (n = 84), Tam Hang South (n = 62), and Duoi U’Oi (n = 60) were sampled and analysed for the present study (Supplementary Tables S5 and S7). Enamel was first cleaned mechanically using a handheld dental drill equipped with a diamond-tipped burr. Using either a diamond-tip cutting wheel or a diamond-tipped burr, samples—powder or fragment—were then taken along the full height of the crown for each specimen. When enamel fragments rather than powder were sampled, the complete enamel pieces were crushed using an agate mortar and pestle. Powdered enamel teeth samples were soaked in 1 ml of CH_3_COOH (0.1) M for four hours at room temperature, and then rinsed several times in distilled water and finally dried overnight at 65 °C. Using the carbonate phase of enamel, stable carbon and oxygen isotopic ratios measurement were performed at the "Service de Spectrométrie de Masse Isotopique du Muséum (SSMIM)" in Paris, using a Thermo Scientific Delta V Advantage isotopic mass spectrometer along with a Thermo Scientific Kiel IV Carbonate Device chemical preparer. Isotopic abundances are presented in δ (delta) notation expressed as deviation per mil (‰), where: *δ*^13^C = (^13^C/^12^Csample/^13^C/^12^Cstandard − 1) × 1000 and *δ*^18^O = (^18^O/^16^O_sample_/^18^O/^16^O_standard_ − 1) × 1000.

In this analysis, we used the *δ*^13^C limits corresponding to broad Pre-Industrial environments: − 27.2 ‰ and − 21.3 ‰ as the upper *δ*^13^C limit for closed-canopy forests^121^ and intermediate rainforests and woodland biomes, respectively^74,122^, and − 15.3 ‰ as the lower *δ*^13^C limit for C_4_ savannah-like environment^74^.

### Statistical analyses

Kruskal–Wallis one-way analysis of variance was performed across the dataset on both the novel (Coc Muoi, Tam Hang South, and Duoi U’Oi) and published data (Nam Lot and Tam Hay Marklot) to determine statistical differences in *δ*^13^C_carbon source_ and *δ*^18^O_apatite_ values between sites. For this, samples from Coc Muoi (n = 84), Tam Hang South (n = 62), Duoi U’Oi (n = 60), as well as already-published sites of Nam Lot (n = 57^22^) and Tam Hay Marklot (n = 72^20^) were used. Crown area dimensions between sites were also investigated for *R. unicolor* (n = 65) and *S. scrofa* (n = 61). Kruskal–Wallis test was chosen over parametric ANOVA for all analyses as preliminary tests were carried to check for normally distributed data and equal variance, which revealed that non-parametric testing was to be used. All statistical analyses were conducted using the free program R software (R Core Team, 2018).

## Supplementary Information

Supplementary Information.
