# Supplementary material for: A multi-proxy approach to exploring Homo sapiens’ arrival, environments and adaptations in Southeast Asia
Source: Sci Rep. 2021 Oct 26;11:21080. doi: 10.1038/s41598-021-99931-4 (PMC8548499; doi:10.1038/s41598-021-99931-4)
Supplement: Supplementary file 1 — Supplementary Information. [file 41598_2021_99931_MOESM1_ESM.pdf]

Supplementary Information

A Multi-proxy approach to exploring *Homo sapiens*' arrival, environments and adaptations in Southeast Asia

Anne-Marie Bacon[1*], Nicolas Bourgon[2, 3], Frido Welker[4], Enrico Cappellini[4], Denis Fiorillo[5], Olivier Tombret[5], Nguyen Thi Mai Huong [6], Nguyen Anh Tuan [6], Thongsa Sayavonkhamdy[7], Viengkeo Souksavatdy[7], Phonephanh Sichanthongtip[7], Pierre-Olivier Antoine[8], Philippe Duringer[9], Jean-Luc Ponche[10], Kira Westaway[11], Renaud Joannes-Boyau[12,13], Quentin Boesch[9], Eric Suzzoni[14], Sébastien Frangeul[14], Elise Patole-Edoumba[15], Alexandra Zachwieja[16], Laura Shackelford[17], Fabrice Demeter[18, 19], Jean-Jacques Hublin[2, 20], Élise Dufour[5]

[1] UMR 8045 BABEL, CNRS, Université de Paris, Faculté de chirurgie dentaire, Montrouge, France.
[2] Department of Human Evolution, Max Planck Institute for Evolutionary Anthropology, Leipzig, Germany.
[3] Applied and Analytical Palaeontology, Institute of Geosciences, Johannes Gutenberg University, Mainz, Germany
[4] Section for Evolutionary Genomics, GLOBE Institute, University of Copenhagen, Denmark.
[5] UMR 7209 Archéozoologie, Archéobotanique : Sociétés, Pratiques, Environnements, Muséum National d'Histoire Naturelle, CNRS, Paris, France.
[6] Anthropological and palaeoenvironmental Department, Institute of Archaeology, Hoan Kiem District, Ha Noi, Vietnam.
[7] Department of Heritage, Ministry of Information, Culture and Tourism, Lao People's Democratic Republic.
[8] Institut des Sciences de l'Évolution de Montpellier, Université de Montpellier, CNRS, IRD, EPHE, Montpellier, France.
[9] Université de Strasbourg, Ecole et Observatoire des Sciences de la Terre (EOST Géologie), Institut de Physique du Globe de Strasbourg (IPGS) (CNRS/UMR 7516), Institut de Géologie, Strasbourg, France.
[10] UMR 7362 Laboratoire Image Ville et Environnement, Institut de Géologie, Strasbourg, France.
[11] 'Traps' MQ Luminescence Dating Facility, Department of Earth and Environmental Sciences, Macquarie University, Sydney, Australia.
[12] Geoarchaeology & Archaeometry Research Group, Southern Cross University, Lismore, Australia.
[13] Institute of Vertebrate Paleontology and Paleoanthropology (IVPP) of the Chinese Academy of Sciences, Beijing, China.
[14] Spitteurs Pan, Technical cave supervision and exploration, La Chapelle-en-Vercors, France.
[15] Muséum d'Histoire Naturelle, La Rochelle, France.
[16] Department of Biomedical Sciences, University of Minnesota Medical School, Duluth, MN, USA.
[17] Department of Anthropology, University of Illinois at Urbana-Champaign, Urbana IL, USA.
[18] Lundbeck Foundation GeoGenetics Centre, GLOBE Institute, Copenhagen, Denmark.
[19] UMR 7206 Eco-anthropologie, Muséum National d'Histoire Naturelle, CNRS, Paris, France.
[20] Collège de France, Chaire de Paléoanthropologie, Paris, France.

Material and Methods

Context and nature of the fossiliferous deposits: The fossil localities studied are located in the northern parts of Vietnam (Duoi U'Oi and Coc Muoi) and Laos (Tam Hang South, Nam Lot, and Tam Hay Marklot), in the same 23°-20° latitudinal belt (Bacon et al., 2008, 2015, 2018a; Bourgon et al., 2020) (Fig. S1). The landscape of this region contains typical tower karsts that emerge from the alluvial plain covered by cultivated fields and houses. All five karstic sites formed in massive limestone beds, Carboniferous to Triassic in age, and present the same type of sedimentary infillings in the caves and cavities.

The deposits consist of either well-cemented breccias plastered as relics on the walls and roofs of caves and cavities and/or silty to sandy clays located on the cave floor. The analysis of the sites suggests that the breccias were created principally by reworked carbonate clasts from the limestone massif, some speleothems and sandy to silty clays, along with fossil remains. These consituents were carried and deposited by water inside the cave network (mainly endokarstic processes mixed with variable exokarstic material) (Duringer et al., 2012). Fossiliferous deposits are most likely a result of a long transportation process of remains through the subterraneous cave networks, over several thousands of years or more. The detailed description of sites can be found elsewhere in original publications.

Conservation of assemblages and taphonomic biases: The five assemblages contain mainly isolated teeth of a large array of mammals including Artiodactyla, Perissodactyla, Proboscidea, Carnivora, Primates and Rodentia (> 5 kg). Small-bodied microvertebrates are lacking. The analysis of these assemblages revealed that they share taphonomic pathways, due to the action of biotic (rodents and carnivores) and abiotic (water flows) agents, through comparable processes of deposition (Duringer et al., 2012).

The high percentages of teeth (> 70%) showing gnawing marks in all assemblages indicate the significant role of porcupines (*Hystrix* sp.), as the last accumulator agent of remains of carcasses at the sites, before being buried in the sediments and transported into the cave network. The differential preservation of lower *versus* upper teeth has been associated with the capacity of porcupines to collect all detachable and transportable remains of carcasses of animals of various body-sizes (~5 kg up to 5,000 kg), either mandibles for large-sized ungulates or cranial remains with maxillae for smaller ones (muntjacs), or complete jaws in some cases (wild pigs) (Bacon et al., 2015). Factors involved in the selection of remains by porcupines might be weight, size, and density of elements (Brain, 1981). Therefore, this capacity of porcupines to collect a wide range of animals > 5kg, means that assemblages are representative of the species diversity. Furthermore, as previously noticed by Brain (1981) for African sites "*It is evident that the minimum numbers of individuals animal represented by the porcupine collected remains do indeed mirror the actual abundance of the antelope species*", it can be reasonably proposed that assemblages are also representative of the abundance of species at a local scale.

Brief description of sites and dating: The five mammal assemblages are bracketed by ages obtained from the dating of flowstones (Uranium-series) and/or cave sediments (red thermoluminescence [TL], single-grain optically stimulated luminescence [SG-OSL], and post-infrared infrared-stimulated luminescence [pIR-IRSL]). Only the Marklot fauna has been dated by direct dating on a few numbers of teeth (combined ESR/U-series) due to the lack of suitable

sediments to date. Therefore, these multiple and independent dating techniques provide a reliable age for the fauna assemblages. We present a brief description of sites and dating results from the oldest to the youngest:

Coc Muoi cave, Northeast Vietnam: The site is close to the Chinese border 155 km NNE from Hanoi in the Lang Son province. The site is a cave situated in a small isolated hill, 361 m above the sea level (Bacon et al., 2018a). The results of both the SG-OSL and pIR-IRSL dating of the sandy clays containing bones and teeth indicate that the sediments were deposited in the cave between 148 and 117 ka. The results of two U-series dating of the overlying flowstone, 114 and 108 ka, represent the minimum age for breccia and fossil accumulation (Fig. S2a).

Tam Hang South rock shelter, Northeast Laos: The site is a group of rock shelters situated along the wall of the Mountain of Pà Hang, in the Huà Pan province, at an elevation of 1120 m. The Mountain also includes other major Pleistocene to Holocene sites, Nam Lot cave, Tam Pà Ling, and Tam Hang Central (Bacon et al., 2015; Patole-Edoumba & Demeter, 2019). The fossiliferous breccias of Tam Hang South were located at the basal part of the Pà Hang cliff, plastered against the wall of the rock shelter. The sedimentary infilling consists of argillaceous-rich breccias separated by flowstones, the dating of which was based on different techniques: red TL, SG-OSL and U-series results provided an age range of 94 - 60 ka (Fig. S2b).

Nam Lot cave, Northeast Laos: The cave is located along the cliff of the Pà Hang Mountain, 250 m to the east of the Tam Hang site (Duringer et al., 2012; Bacon et al., 2015, 2018b). The fauna assemblage was derived from the breccia and from the silty to sandy clays in the lower part of the cave close to the entrance. Based on red TL, SG OSL and U-series dating, the age range for the assemblage is 86 - 72 ka (Bacon et al., 2015) (Fig. S3a).

Duoi U'Oi cave, Northwest Vietnam: The cave is located on the other side of the border from Laos, around 100 km away from the Pà Hang Mountain (Bacon et al., 2008, 2018b). It is a lowland site situated around ~5 m above the alluvial plain. The red TL and SG-OSL dating of the sedimentary breccia unit which contains the faunal assemblage, and the U-series dating of the calcitic floor that crosses the fossiliferous breccia, provide an age range of 70 – 60 ka (Bacon et al., 2015) (Fig. S3b). The age constraints on the fauna are also supported by additional radiocarbon dates on teeth, which are > 32 cal kBP (Wood et al. 2016, 2021).

Tam Hay Marklot cave, Northeast Laos: The cave is located in the Huà Pan province, northeastern Laos (Bourgon et al., 2020). Remains of the faunal assemblage have been found in a soft sandy to gravelly clays that covered almost the entire surface of the cave (Fig. S3c). Based on combined ESR and U-Th dating of five mammalian teeth, the age range of the fauna is 38.4 – 13.5 ka.

Carbon stable isotopes: The carbon isotopic composition of bioapatite reflects that of diet, whereas the oxygen mainly reflects ingested water (DeNiro & Epstein, 1978; Lee-Thorp et al., 1989; Sponheimer & Lee-Thorp, 1999; Cerling & Harris 1999; Sullivan & Krueger, 1981; Longinelli, 1984; Luz et al., 1984; Passey et al., 2005).

For the geographic area in this study, a $\delta^{13}C$ range of -37 ‰ to -23 ‰ is considered to represent C_3 plants, as higher values (> -23 ‰) are restricted to particular context and species (Smith & Epstein, 1971; O'Leary, 1988; Kohn, 2010). C_4 plants present higher $\delta^{13}C$ values of -17 ‰ to -10 ‰ (Smith and Epstein 1971; O'Leary 1988). The burning of fossil fuel over the past 150 years has decreased the $\delta^{13}C$ of atmospheric CO_2, consequently this has influenced the $\delta^{13}C$ of all living organisms. As the $\delta^{13}C$ values of plants are based on modern samples, a correction must be applied (~ +1.7 ‰) (Friedli et al., 1986) when comparing with organisms

that predate the fossil-fuel-induced atmospheric CO_2 shift. Therefore, the upper δ^{13}C limit for C_3 plants used for the present study has been shifted from -23 ‰ to -21.3 ‰, and the lower limit for C_4 plants from -17 ‰ to -15.3 ‰.

The composition of the fauna isotopic diet is then metabolized and incorporated into the tissues of animals (Lee-Thorp et al., 1989). However, trophic fractionation occurs when the food's carbon is incorporated into the tissues of animals (bone collagen, enamel carbonate, hairs, etc.) and also varies according to the animals (DeNiro & Epstein, 1978; Lee-Thorp et al., 1989; Cerling & Harris 1999; Passey et al., 2005). Although arguably little is known of the digestive physiologies of fossil species to accordingly adjust discrimination factors, some comparable modern data are nevertheless available (Cerling & Harris 1999; Balasse, 2002; Passey et al., 2005, Lee-Thorp & Van der Merwe, 1987; Bocherens, 2002; Bocherens et al., 2011; Cerling et al., 2004; Fox-Dobbs et al., 2006; Sponheimer et al., 2013). Furthermore, Tejada-Lara et al. (2018) recently established a relationship where body mass can be used to predict ^{13}C isotope enrichment in mammals. As mentioned by the authors, a single systematical enrichment factor, rather than multiple ones, can alter interpretations of animal ecologies and thus potentially lead to less precisely reconstructed environments. In this paper, species-specific enrichment factors were calculated for each taxon according to mean body mass following the equations proposed by Tejada-Lara et al. (2018).

As explained in Tejada-Lara et al. (2018), the formulae use log transformed (ln) body mass. Additionally, in order to obtain the ‰ isotope enrichment value, the obtained diet-bioapatite (ε^*) needs to be inverted (e^x). For all intent and purposes and intent, this results in the following formulae:

General: $\varepsilon^*_{\text{diet-bioapatite}} = e^{2.4 + 0.034\ (\ln BM)}$

Foregut fermenter: $\varepsilon^*_{\text{diet-bioapatite}} = e^{2.34 + 0.05\ (\ln BM)}$

Hindgut fermenter: $\varepsilon^*_{\text{diet-bioapatite}} = e^{2.42 + 0.032\ (\ln BM)}$

The enrichment factor of the giant panda (*Ailuropoda melanoleuca*) was not calculated, as was Tejada-Lara et al. (2018) clearly demonstrated that the giant panda's calculated enrichment factor was incorrect, and also not suited for calculating enrichment factor of carnivores. As such, a $\varepsilon^*_{\text{diet-bioapatite}}$ δ^{13}C enrichment of +10.51 ‰, found in the literature (Han et al., 2016) was used for the giant panda (*Ailuropoda melanoleuca*). While calculating the enrichment factor is clearly not suited to carnivores, an average carnivore-herbivore enamel spacing of -1.3 ‰ was established (Clementz et al., 2009). This spacing can then be added to their $\delta^{13}C_{apatite}$, resulting in an averaged apatite value of their prey, which can be transformed using the general formulae from Tejada-Lara et al. (2018). This effectively obtains estimates of the δ^{13}C values of the environment in which carnivores hunted at the time. Finally, no $\varepsilon^*_{\text{diet-bioapatite}}$ δ^{13}C enrichment are available for ursids and calculating enrichment factor was not suited due to their carnivore digestive physiology. Since ursids are not strict carnivore, obtaining $\delta^{13}C_{source}$ values, in a similar way to carnivores as described above, was also not possible. As such, we used a $\varepsilon^*_{\text{diet-bioapatite}}$ δ^{13}C enrichment of 13.3 ‰ from pigs (Passey et al., 2005), as the closest and most suitable alternative for omnivorous taxa.

Such conversion accounts for differences in faunal assemblages between sites, thus ensuring better comparisons between taxa that can be relating directly to the environment. Finally, we use the notation "$\delta^{13}C_{\text{carbon source}}$" instead of the commonly seen "$\delta^{13}C_{diet}$" (Louys & Roberts, 2020; Tejada-Lara et al., 2020) as $\delta^{13}C_{apatite}$ of carnivores were also tentatively converted to $\delta^{13}C_{\text{carbon source}}$ values (*i.e.* plant δ^{13}C values). The "$\delta^{13}C_{\text{carbon source}}$" thus effectively reflects the initial carbon uptake derived from plants, a terrestrial food web's primary carbon

source, and not always (namely in the case of carnivores) the δ^{13}C value of the consumer's diet. While $\delta^{13}C_{apatite}$ values were converted to $\delta^{13}C_{carbon\ source}$, the $\delta^{18}O_{apatite}$ values were not similarly converted to drinking water δ^{18}O values because empirically determined water-enamel ^{18}O fractionation (or closely related ones) is not determined for too many modern-day analogue species of the investigated fossil taxa (or closely related ones).
During every mass spectrometer run (15 runs in total for the three sites), an internal laboratory standard (Marble LM, accepted δ^{13}C = +2.13 ‰ and δ^{18}O = −1.83 ‰) was analysed that has been normalised to the International Atomic Energy Agency reference material (NBS 19). It was used for tooth sample correction and for controlling the precision of the mass spectrometer (1σ (δ^{13}C) = 0.031 ‰ and 1σ (δ^{18}O) = 0.044 ‰ for the 15 runs). Each tooth sample was usually analysed one or two times except for 6 individuals from Coc Muoi and Duoi U'Oi that were analysed three times. The repeated analyses were used to test for intra-individual heterogeneity and analytical reproducibility of enamel analysis (Table S1). Maximum standard deviations were 0.136 ‰ and 0.165 ‰ for δ^{13}C and δ^{18}O enamel analysis, respectively.

Palaeoproteomics, Methods: Two extracts (13.4 mg and 12.0 mg, respectively) of the lower incisor (NL 433) were prepared and analyzed using an HCl-based demineralization protocol without using a digestive protease, as previously described (Cappellini et al., 2019). Raw data were searched against an enamel-specific proteome database including Hominoidea and *Macaca* entries for the following protein sequences: COL1A1, COL1A2, COL17A1, ALB, TUFT1, ODAM, DSPP, AMELX, AMELY, AMBN, ENAM, KLK4, MMP20, FAM20A, FAM20C), obtained from UniProt and Genbank. Missing isoforms (for AMELX, AMELY, AMBN, ENAM) and predicted protein sequences for ancestral nodes within Hominidae (for ALB, AMBN, AMELX, AMELY, AMTN, COL17A1, ENAM, MMP20) were added to this protein sequence database, as previously described (Welker et al., 2020), to minimize cross-species proteomic effects (Welker, 2018). MaxQuant (v. 1.6.3.4) settings mirrored those described in Welker et al. (2020), with the omission of histidine to aspartic acid (H>D) and histidine to hydroxyglutamate as variable modifications. Deamidation was calculated as described in Mackie et al. (2018), with values grouped at the protein-level.

To assign a biological identity to this specimen, we retained only those proteins with at least 2 unique peptides. Subsequently, we reconstructed the ancient protein sequence of the Nam Lot individual, obtained relevant SAPs, and created a heatmap of the amino acid distances between species pairs, including the Nam Lot individual (packages seqinr v. 3.6.1, (Charif & Lobry, 2007), and pheatmap v. 1.0.12, (Kolde, 2015), on the resulting variant matrix (restricted to ALB, AMELX, AMBN, ENAM and COL17A1). Simultaneously, the variant matrix was interrogated for sites in the Nam Lot individual that are uniquely present in the genus *Homo* or in the genus *Pongo*. The quality of spectra overlapping such positions was then manually validated. A similar procedure was used for the two reported AMELY-specific peptides. These did not represent high-confidence spectra, and as a result we do not assign the specimen to a biological sex.

Proteomic data generated for the Nam Lot specimen has been deposited in ProteomeXchange under accession number PXD027426.

Results: Extraction of the Nam Lot 433 (NL 433) enamel incisor fragment resulted in the acquisition of 45,237 MS2 spectra combined over two independent LC-MS/MS injections. After quality filtering (see Methods), we identified 7 unique protein groups (AMELX, AMBN,

ENAM, MMP20, ALB, and COL17A1; Table S2), with AMELX represented by two isoforms. The Nam Lot enamel is therefore composed of the same core enamel proteome as other Pleistocene enamel proteomes (Cappellini et al., 2019; Welker et al., 2019, 2020). The deamidation of these proteins is higher than that observed for a small list of contaminants identified (Table S2), providing an indication that these proteins are likely to be of endogenous origin.

As with previous Pleistocene enamel proteomes, sequence coverage for AMTN and MMP20 is limited to small, uninformative sequence regions. We therefore restricted phylogenetic analysis to AMELX, AMBN, ENAM, ALB, and COL17A1. The reconstructed protein sequence of the NL 433 specimen assigns it unambiguously to the genus *Pongo* (Fig. S4a). All retained peptide matches are 100% matches to the *Pongo* sequences, with no unique matches to the genus *Homo*. In several proteins, the Nam Lot specimen provided protein sequences overlapping uniquely-derived single amino acid polymorphisms of the genus *Pongo* (Fig. S4b-d). For those stretches where we have protein sequence data for the ancient specimens, both species within the genus *Pongo*, *P. abelli* and *P. pygmaeus*, carry the same protein sequence. Therefore, we cannot assign the Nam Lot 433 specimen to a more specific orangutan species.

Supplementary Figures

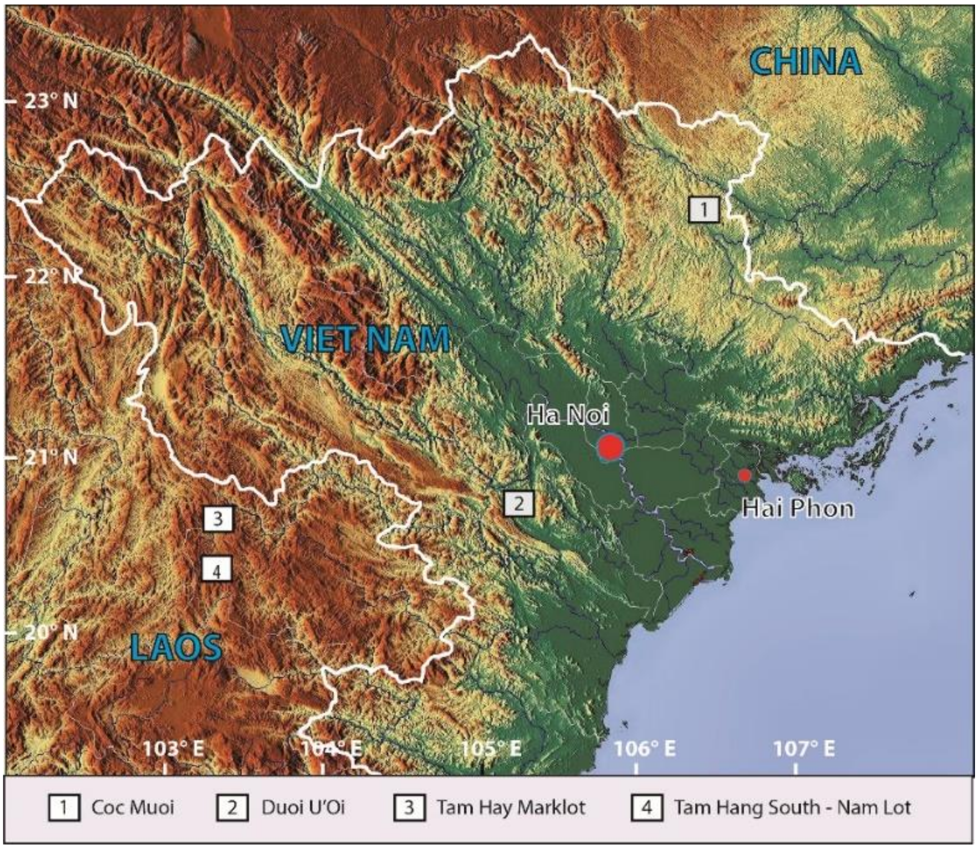

Figure S1: Satellite image of the studied area with the location of sites (Figure made by P. Duringer). The satellite image is from the website (http://www.maps-for-free.com/), and reworked using the software Illustrator CS5 (version 15.0.0).

Figure S2: Sediments and datings.

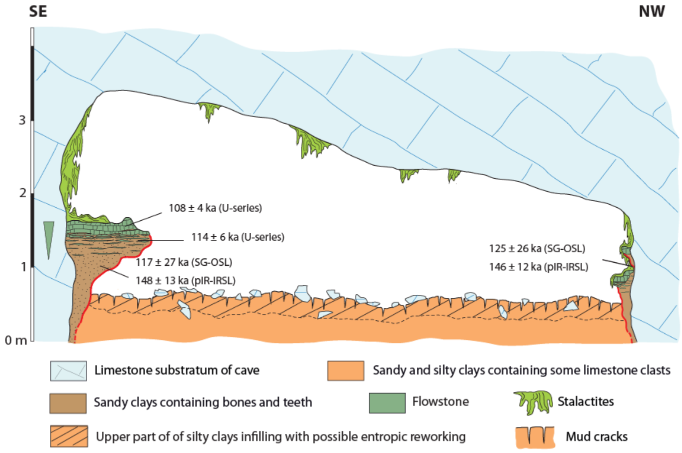

a. Vertical section of the Coc Muoi cave, with results of U-series dating of the flowstones and luminescence dating (SG-OSL and pIR-IRSL) of the breccia unit (drawing of Philippe Duringer and Jean-Luc Ponche published in Bacon et al., 2018; Fig. 5).

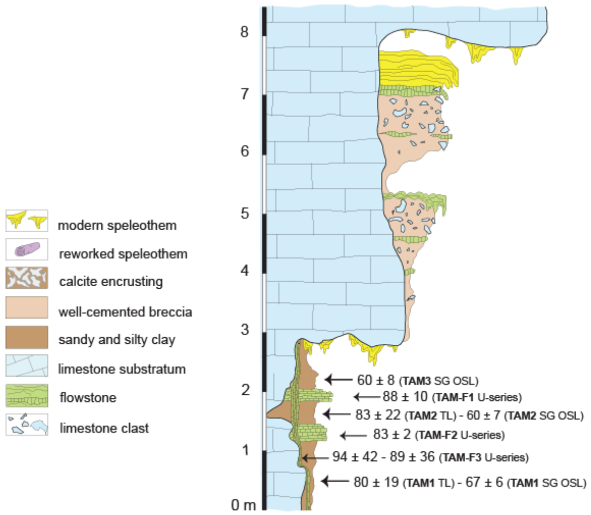

b. Vertical section of the wall of the Tam Hang rock shelter, with results of U-series dating of the flowstones and luminescence dating (TL and SG-OSL) of the breccia unit (drawing of Philippe Duringer and Jean-Luc Ponche published in Bacon et al., 2015; Fig. 2).

Figure S3: Sediments and datings.

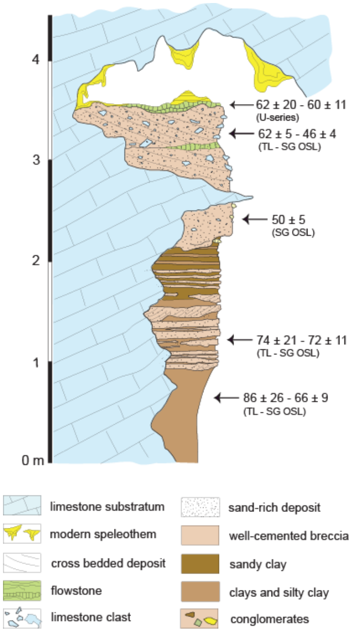

a. Vertical section of the breccia unit of Nam Lot cave, with results of U-series dating of the flowstones and luminescence dating (TL and SG-OSL) of the breccia (drawing of Philippe Duringer and Jean-Luc Ponche published in Bacon et al., 2015; Fig. 3).

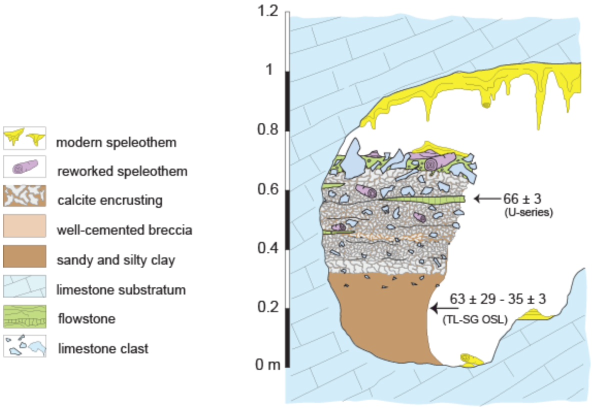

b. Vertical section of the breccia unit of Duoi U'Oi cave, with results of U-series dating of the flowstones and luminescence dating (TL and SG-OSL) of the breccia (drawing of Philippe Duringer and Jean-Luc Ponche published in Bacon et al., 2015; Fig. 4).

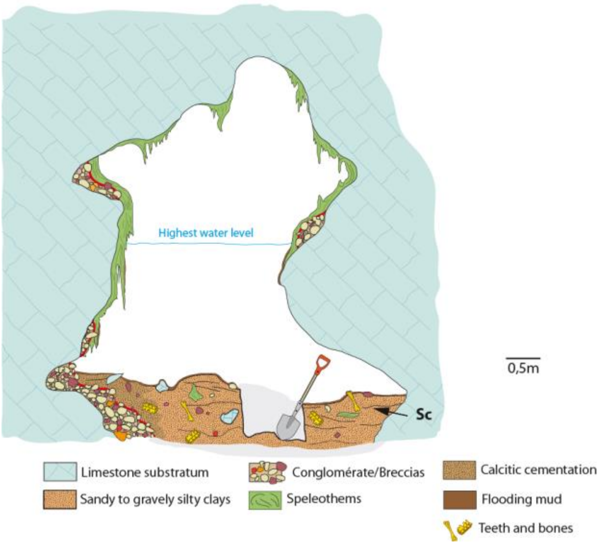

c. Section of the corridor in the Tam Hay Marklot cave. Specimens of the faunal assemblage have been found in the soft sandy to gravelly clays that covered almost the entire soil of the cave (drawing of Philippe Duringer and Jean-Luc Ponche published in Bourgon et al., 2020; Fig. SI7). The age range of the fauna is 38.4 – 13.5 ka.

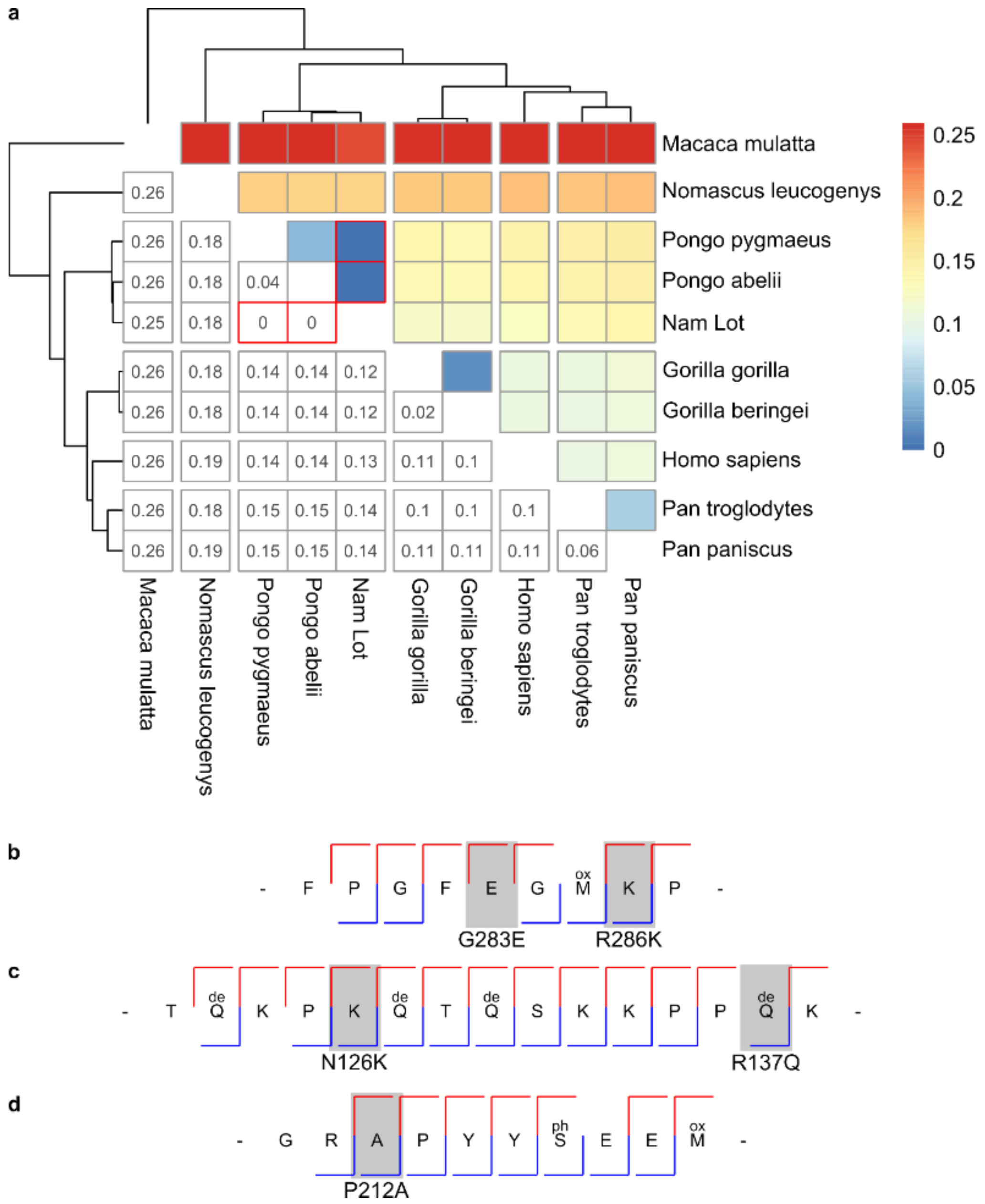

Figure S4. Palaeoproteomic analysis reveals the Nam Lot 433 lower incisor to represent a *Pongo* sp. specimen. **a**. Heatmap clustering of amino acid sequence distances between Nam Lot and extant Hominoidea, with *Macaca mulatta* as an outgroup. The phylogenetic tree based on protein distances reflected the commonly proposed hypothesis among Hominidae. **b**. Ion fragment series of a peptide overlapping *Pongo*-derived SAPs at positions 283 and 286 in AMBN. **c**. Ion fragment series of a peptide overlapping *Pongo*-derived SAPs at positions 126 and 137 in ENAM. **d**. Ion fragment series of a peptide overlapping *Pongo*-derived SAP at position 212 in ENAM. Figure S4 was created using the Package ‘pheatmap’ (v.1.0.12), available using this link (https://mran.microsoft.com/snapshot/2017-09-01/web/packages/pheatmap/pheatmap.pdf).

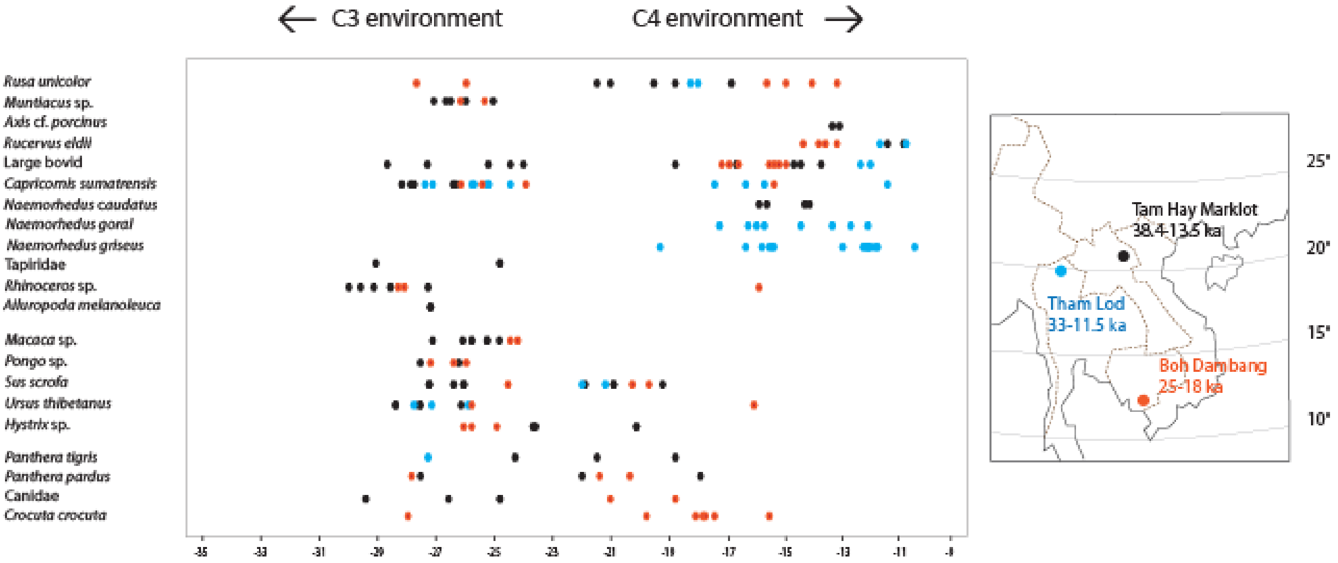

Figure S5. Distribution of $\delta^{13}C_{carbon\ source}$ values (‰ VPDB) in herbivores, omnivores and carnivores from Tham Lod rockshelter, Thailand (33 – 11.5 ka; Suraprasit et al., 2020), Tam Hay Marklot, Laos (38.4 – 13.5 ka; Bourgon et al., 2020), and Boh Dambang, Cambodia (25 -18 ka; Bacon et al., 2018c) (see Supplementary Annexes S4-S5 for $\delta^{13}C_{carbon\ source}$ values).

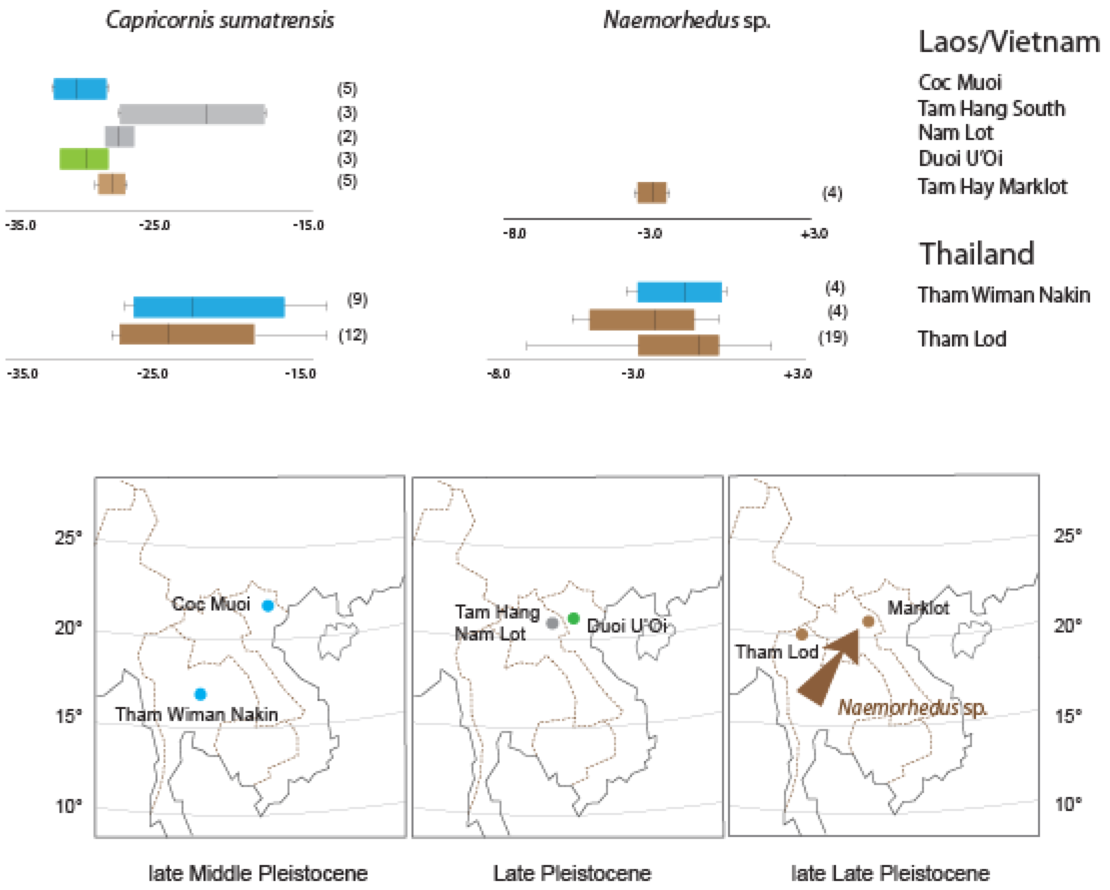

Figure S6. Ranges of $\delta^{13}C_{carbon\ source}$ values in *Capricornis sumatraensis* and *Naemorhedus* sp. in sites from Thailand, Laos, and Vietnam. *N.* cf. *caudatus* at Tam Hay Marklot, Laos (38.4 – 13.5 ka); *N. crispus* and *N. griseus* at Tham Lod, Thailand (33 – 11.5 ka; Suraprasit et al., 2020). Below, location of sites by period, with the hypothesis of movements of populations of *Naemorhedus* species, coming likely from southern latitudes where populations had become over the course of time adapted to living in grasslands (see Supplementary Annexes S1, S2, S4 for $\delta^{13}C_{carbon\ source}$ values).

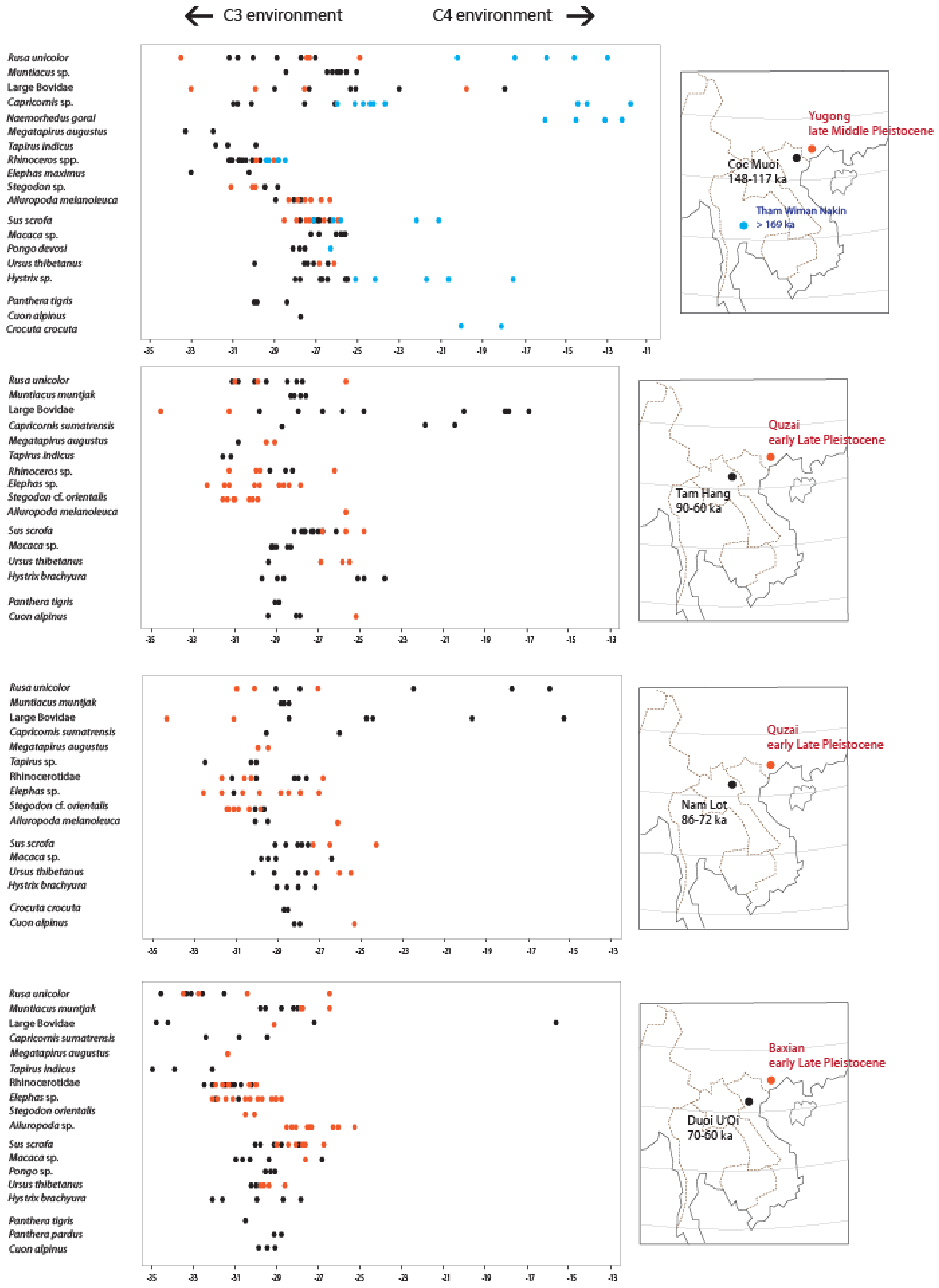

Figure S7. Distribution of $\delta^{13}C_{\text{carbon source}}$ values (‰ VPDB) in herbivores, omnivores and carnivores from Vietnam, Laos, Thailand and southern China sites (see Supplementary Annexes S1-S3 for $\delta^{13}C_{\text{carbon source}}$ values).

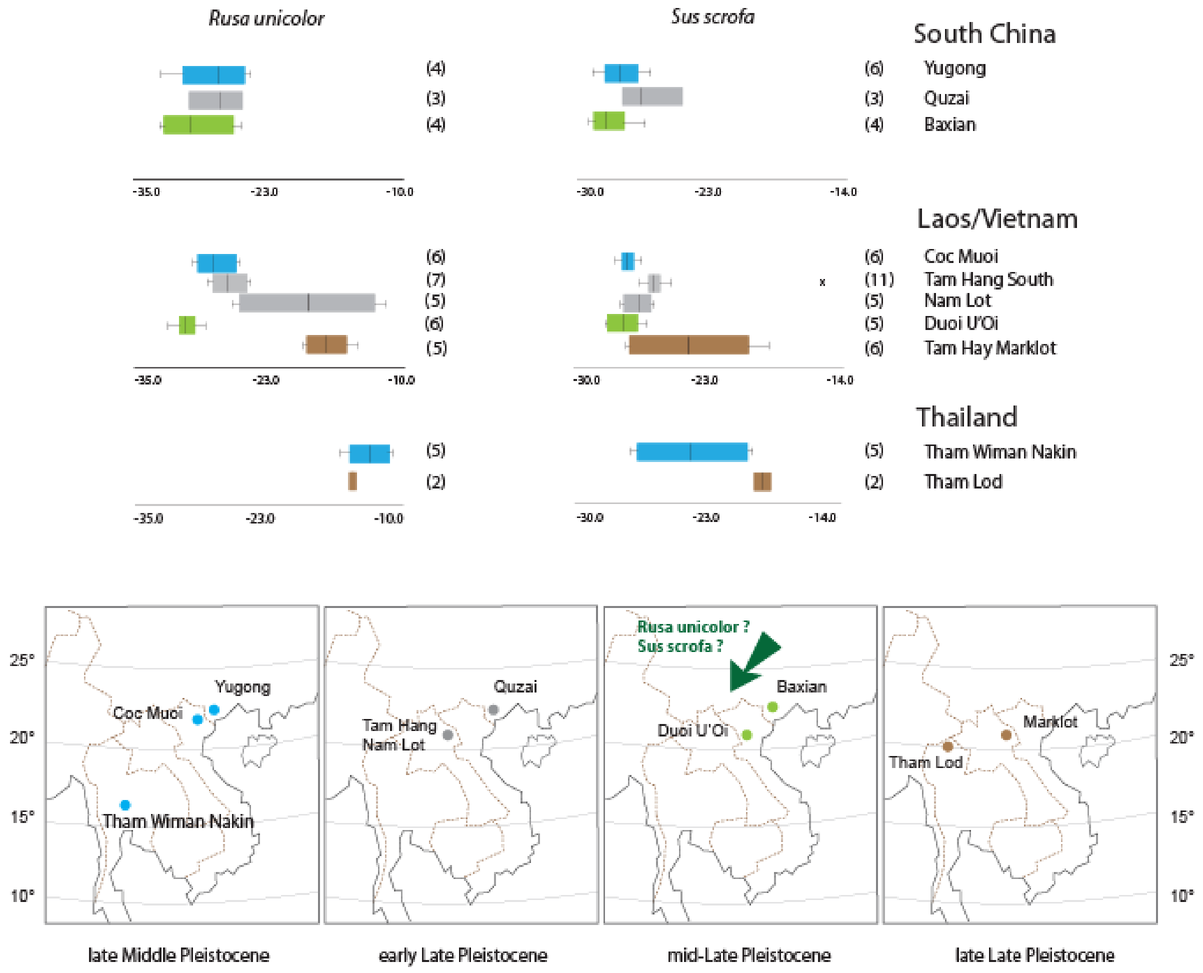

Figure S8. Ranges of $\delta^{13}C_{\text{carbon source}}$ values in *Rusa unicolor* and *Sus scrofa* from Thailand, Laos, Vietnam and southern China sites. Below, location of sites by period, with the hypothesis of movements of populations of *R. unicolor* and *S. scrofa* inferred from the present analysis. Other taxa (rhinoceroses, bears, bovids) could have been also concerned by this range shift (see carbon isotope data in Sun et al., 2019).

Vietnam: Coc Muoi (148 – 117 ka); Duoi U'Oi (70 – 60 ka). Laos: Tam Hang South (94 – 60 ka); Nam Lot (86 – 72 ka); Tam Hay Marklot (38.4 – 13.5 ka). Southern China: Yugong cave, Guangxi (late Middle Pleistocene; Sun et al., 2019); Baxian cave, Guangxi (early Late Pleistocene; Ma et al., 2017; Sun et al., 2019); Quzai cave, Guangxi (early Late Pleistocene; Ma et al., 2019). Thailand: Tham Lod (33 – 11.5 ka; Suraprasit et al., 2020); Tham Wiman Nakin (> ~170 ka; (Esposito et al., 1998, 2002; Pushkina et al., 2010; Suraprasit et al., 2020).

Supplementary Tables

Table S1

site	individual	Std δ^{13}C (‰)	Std δ^{18}O (‰)
Coc Muoi	34890	0.056	0.040
Coc Muoi	34911	0.042	0.036
Coc Muoi	34937	0.136	0.165
Duoi Uoi	34831	0.050	0.151
Duoi Uoi	34845	0.007	0.125
Duoi Uoi	34851	0.044	0.140

Standard deviation of repeated analysis (n=3) for 6 individuals from Coc Muoi and Duoi U'Oi used to estimate the intra-individual heterogeneity and analytical reproductibility of enamel analysis.

Table S2

<table>
<tr><th>Protein</th><th>UniProt Pongo abelii accession</th><th>Source</th><th>Peptides</th><th>Razor+ Unique peptides</th><th>Coverage (%)</th><th>Deamidation N (% ± 1 SD)</th><th>Deamidation Q (% ± 1 SD)</th></tr>
<tr><td>AMELX (Isoform 1</td><td>H2PUX0</td><td>Enamel proteome</td><td>506</td><td>506</td><td>81.2</td><td rowspan="2">92.2 (2.3)</td><td rowspan="2">99.3 (0.3)</td></tr>
<tr><td>AMELX (Isoform 3</td><td>H2PUX0</td><td>Enamel proteome</td><td>500</td><td>74</td><td>82.4</td></tr>
<tr><td>AMBN</td><td>H2PDI5</td><td>Enamel proteome</td><td>208</td><td>208</td><td>353</td><td>99.9 (0.0)</td><td>98.9 (0.5)</td></tr>
<tr><td>ENAM</td><td>H2PDI6</td><td>Enamel proteome</td><td>376</td><td>376</td><td>17.9</td><td>99.2 (0.3)</td><td>96.1 (1.2)</td></tr>
<tr><td>MMP20</td><td>H2NF32</td><td>Enamel proteome</td><td>7</td><td>7</td><td>8.7</td><td>100 (0.0)</td><td>NA</td></tr>
<tr><td>AMTN</td><td>H2PDI4</td><td>Enamel proteome</td><td>2</td><td>2</td><td>8.7</td><td>NA</td><td>100 (0.0)</td></tr>
<tr><td>ALB</td><td>Q5NVH5</td><td>Enamel proteome</td><td>16</td><td>16</td><td>17.6</td><td>99.0 (1.0)</td><td>100 (0.0)</td></tr>
<tr><td>COL17A1</td><td>H2NBI5</td><td>Enamel proteome</td><td>19</td><td>19</td><td>4.1</td><td>NA</td><td>84.9 (15.7)</td></tr>
<tr><td>A2VCT4</td><td></td><td>Contaminant</td><td>2</td><td>2</td><td>3.7</td><td rowspan="3">50.8 (28.4)</td><td rowspan="3">NA</td></tr>
<tr><td>ENSBTAP00000024146</td><td></td><td>Contaminant</td><td>2</td><td>2</td><td>1.6</td></tr>
<tr><td>XP_585019</td><td></td><td>Contaminant</td><td>2</td><td>2</td><td>3.3</td></tr>
</table>

Protein group LC-MS/MS results and coverage. Deamidation was calculated for the contaminants together. Peptide counts taken from the MaxQuant proteingroups.txt file.

Table S3

Order	Common name	Taxon	Coc Muoi	Tam Hang South	Nam Lot	Duoi U'Oi	Tam Hay Marklot
**Artiodactyla**	Sambar	*Rusa unicolor*	X	X	X	X	X
	-	medium-sized cervid	X	X			
	Thamin	*Rucervus eldii*					X
	Hog deer	*Axis porcinus*					cf.
	Indian muntjac	*Muntiacus muntjak*		X	X	X	
	-	*Muntiacus* sp.	X				X
	Kouprey	*Bos sauveli*	cf.	cf.			
	Gayal	*Bos frontalis*					cf.
	-	*Bos* sp.			X		
	Large-sized Bovidae	Bovidae	X	X	X	X	X
	Water buffalo	*Bubalus bubalis*		X	X	cf.	X
	Southern serow	*Capricornis sumatraensis*		X	X	X	cf.
	-	*Capricornis* sp.	X				
	Chinese goral	*Naemorhedus caudatus*					cf.
	-	*Naemorhedus* sp.					
	Wild boar	*Sus scrofa*	X	X	X	X	X
	Bearded pig	*Sus barbatus*		cf.		X	cf.
	-	*Sus* sp.					
**Perissodactyla**	Giant tapir	*Megatapirus augustus*	X	X			
	-	*Tapirus indicus intermedius*		cf.			
	Malayan tapir	*Tapirus indicus*	X			X	
	-	*Tapirus* sp.			X		X
	Indian rhinoceros	*Rhinoceros unicornis*	cf.	X	X	X	
	Javan rhinoceros	*Rhinoceros sondaicus*	X	X	X	X	X
	Sumatran rhinoceros	*Dicerorhinus sumatrensis*	X		0	X	X
	-	*Rhinoceros* sp.		X	X	X	
**Proboscidea**	Asian elephant	*Elephas maximus*	X				
	-	*Elephas* sp.		X	X	X	
	Stegodon	*Stegodon orientalis*		X	cf.		
	-	*Stegodon* sp.	X				

Faunal lists of the five sites (Artiodactyla, Perissodactyla, and Proboscidea).

Table S4

Order	Common name	Taxon	Coc Muoi	Tam Hang South	Nam Lot	Duoi U'Oi	Tam Hay Marklot
**Carnivora**	Dhole	*Cuon alpinus*	X		X	X	?
	Dhole	*Cuon alpinus antiquus*		cf.			
	-	*Cuon* sp.					
	Wild dogs	Canidae					X
	Hog-badger	*Arctonyx collaris*				X	
	Hog-badger	*Arctonyx collaris rostratus*		cf.			
	Eurasian badger	*Meles meles*		X	X	X	
	Large tooth ferret-badger	*Melogale personata*		X			
	Yellow-throated marten	*Martes flavigula*		cf.	X		
	-	*Martes* sp.	X				
	Large Indian civet	*Viverra zibetha*		X	X	X	
	Large-spotted civet	*Viverra megaspila*				cf.	
	Common palm civet	*Paradoxurus hermaphroditus*		X			
	-	*Paradoxurus* sp.					
	-	large-sized meline	X				
	-	small-sized meline	X				X
	Tiger	*Panthera tigris*	X	X		X	X
	Leopard	*Panthera pardus*				X	X
	Leopard cat	*Prionailurus bengalensis*		cf.			
	Golden cat	*Felis temmincki*			cf.		
	Clouded leopard	*Neofelis nebulosa*			X		
	-	small-sized felid	X				X
	Spotted hyena	*Crocuta crocuta*			X		
	Asiatic black bear	*Ursus thibetanus*	X			X	X
	Asiatic black bear	*Ursus thibetanus kokeni*		cf.	cf.		
	Sun bear	*Helarctos malayanus*	cf.	X		X	X
	Giant Panda	*Ailuropoda melanoleuca*	X		X		X
	-	*Ailuropoda* sp.					
**Primates**	Orangutan	*Pongo pygmaeus*		X	X	X	
	Orangutan	*Pongo devosi*	X				
	-	*Pongo* sp.					X
	Macaque	*Macaca* sp.	X			X	X
	-	Colobine	X	X	X	X	
	Gibbon	*Hylobates* sp.	X	X		X	
		Hominine				X	
**Rodentia**	Porcupine	*Hystrix brachyura*		X	X	X	
	-	*Hystrix* sp.	X				x
	Brush-tailed porcupine	*Atherurus macrourus*					cf.
	-	*Atherurus* sp.	X				

Faunal lists of the five sites (Carnivora, Primates, and large Rodentia).

Table S5

Site	Taxon	Crown area	C13 / O18
**Coc Muoi**	*Rusa unicolor*	11 rp3, 5 lp3	3 rm3, 3 lm3
	Muntiacus sp.	2 lm3, 2 rm3	3 lm3, 2 lP2/P3, 2 lP3/P4
	Large-sized Bovidae (*Bos* cf. *sauveli*)	-	6 rm3
	Capricornis sp.	1 lm3, 1 rm3	2 rm1/m2, 3lm1/m2
	Megatapirus augustus	-	1 m, 1 p3
	Tapirus indicus	-	1 p3, 2 p4
	Rhinocerotidae	-	8 p4, 2 M3, 2 m3
	Elephas maximus	-	2 m
	Stegodon sp.	-	2 milk teeth
	Ailuropoda melanoleuca	-	2 lM1, 1 rM1
	Sus scrofa	8 lp3, 5 rp3	5 lm3, 1 m3
	Macaca sp.	2 lm3	3 c/C, 1 lM3, 1 lm1/m2
	Pongo devosi	-	1 lI1, 1rM1, 1 lM, 1 lm1
	Ursus thibetanus/*Ursus* sp.	-	2 lM1, 1 lM2, 1 rm3, 1 lm3
	Hystrix sp.	-	8 I/i
	Atherurus sp.	-	3 I/i
	Panthera tigris	-	2 lP3, 1 lm1
	Small-sized Felidae	-	2 rP4
	Cuon alpinus	-	1 rP4
**Tam Hang South**	*Rusa unicolor*	3 lp3	2 lp2, 3 m, 1 M, 1 rP3
	Muntiacus muntjak	4 lm3, 4 rm3	4 rm3
	Large-sized Bovidae	-	4 rp4, 1 lp4, 1 p4, 1 p3, 1 rm3
	Capricornis sumatraensis	1 m3	1M, 1 m, 1 m3
	Megatapirus augustus	-	1 rm1/m2
	Tapirus indicus cf. *intermedius*	-	1 rM1/M2, 1 rp2
	Rhinoceros spp.	-	1 rp4/m1, 1 lm3, 1 ld4
	Sus scrofa	9 rp3, 3 lp3	1 lP4, 4 rP4, 5 M
	Macaca sp.	2 rm3, 5 lm3	6 lm3
	Ursus thibetanus cf. *kokeni*	-	2 rP4
	Helarctos malayanus	-	1 lm2, 1 lm3
	Hystrix brachyura	-	6 I/i
	Panthera tigris	-	2 lP3
	Cuon alpinus cf. *antiquus*	-	1 rp4, 1 lm2, 1 lM1
**Nam Lot**	*Rusa unicolor*	3 lp3, 5 rp3	2 rp3, 2 lm, 1 rM1
	Muntiacus muntjak	1 rm3	2 rM, 1 lM
	Large-sized Bovidae	-	1 rp4, 1 lp4, 1 lm3, 2 M
	Capricornis sumatraensis	1 lm3, 2 rm3	2 rm3
	Tapirus sp.	-	1 lm, 1li
	Rhinoceros spp.	-	2 p/m, 2 ld, 1d, 1 ld2
	Elephas sp.	-	1 m (fragment)
	Stegodon cf. *orientalis*	-	2 m (fragment)
	Ailuropoda melanoleuca	-	1 rm1, 1 lM2
	Sus scrofa	3 rp3, 1 lp3	3 rp4, 1 rm1, 1 fgt
	Macaca sp.	2 lm3	2 i/I, 2 rm3
	Pongo sp.	-	1 i
	Ursus thibetanus cf. *kokeni*	-	2 rm3, 2 lP4
	Hystrix brachyura	-	2 I/I, 3 p/m
	Crocuta crocuta	-	1 lp3, 1 pm, 1 rp2, 1 rp3, 2 lP2
	Small-sized Felidae	-	1 lP3
	Cuon alpinus	-	1 pm, 1 lp3

Site, taxon, and tooth type used for the morphometric (crown area dimension) and the stable carbon (C13) and oxygen (O18) isotope analyses.

Table S6

Site	Taxon	Crown area	C13 / O18
**Duoi U'Oi**	*Rusa unicolor*	3 lp3, 1 rp3	6 lm3
	Muntiacus muntjak	7 lm3, 5 rm3	5 lm3
	Large-sized Bovidae	-	4 m
	Capricornis sumatraensis	2 lm3, 2 rm3	2 rm3, 1 lm3
	Tapirus indicus	-	1 lM1/M2, 2 lP/M
	Rhinocerotidae	-	2 rM2, 5 rM3, 1 rp2
	Elephas sp.	-	2 milk teeth
	Sus scrofa	9 rp3, 5 lp3	5 lp4
	Macaca sp.	7 lm3, 8 rm3	5 i/I
	Pongo pygmaeus	-	2 rp3
	Ursidae	-	2 rI3
	Hystrix brachyura	-	5 I/i
	Panthera tigris	-	1 rm1
	Panthera pardus	-	2 lp4
	Cuon alpinus	-	3 lP4
**Tam Hay Marklot**	*Rusa unicolor*	18 rp3, 16 lp3	5 rm3
	Muntiacus sp.	13 lm3, 4 rm3	5 lm3
	Axis cf. *porcinus*	-	2 lm3
	Rucervus eldii	-	2 lm3
	Large-sized Bovidae	-	5 rp2, 5 lp2
	Capricornis cf. *sumatraensis*	6 lm3, 2 rm3	5 lm3
	Naemorhedus cf. *caudatus*		4 lm3
	Tapiridae/*Tapirus* sp.	-	1 lc, 1 p/m
	Rhinoceros sondaicus	-	1 ld3, 1rd3, 1 lm2, 2 lm3
	Ailuropoda melanoleuca	-	1 rm2
	Sus scrofa	10 rp3, 8 lp3	6 lp4
	Macaca sp.	1 rm3	2 rp, 3 lm1/m2
	Pongo sp.	-	1 c/C, 1 lM
	Ursus thibetanus	-	1 rM2, 2 lM2
	Helarctos malayanus	-	2 lM2
	Hystrix sp.	-	4 I/i
	Panthera tigris	-	1 rp4, 2 lP4
	Panthera pardus	-	1 rP3, 1 lp4, 1 lP4
	Cuon alpinus	-	1 rm1, 1 rP3, 1 lM1
	Canidae	-	1 lM1

Site, taxon, and tooth type used for the morphometric (crown area dimension) and the stable carbon (C13) and oxygen (O18) isotope analyses.

Table S7

	Artiodactyla		Perissodactyla		Proboscidea		Carnivora		Primates		Rodentia	
**Coc Muoi**	35.71 %	30/84	20.23 %	17/84	4.76 %	4/84	16.66 %	14/84	10.71 %	9/84	11.90 %	10/84
**Tam Hang South**	56.45 %	35/62	9.67 %	6/62	-	-	14.51 %	9/62	9.67 %	6/62	9.67 %	6/62
**Nam Lot**	35.08 %	20/57	15.78 %	9/57	5.26 %	3/57	26.31 %	15/57	10.52 %	6/57	7.01 %	4/57
**Duoi U'Oi**	38.33 %	23/60	18.33 %	11/60	3.33 %	2/60	13.33 %	8/60	13.33 %	8/60	13.33 %	8/60
**Tam Hay Marklot**	54.16 %	39/72	9.72 %	7/72	-	-	20.83 %	15/72	9.72 %	7/72	5.55 %	4/72
**Boh Dambang**	50 %	26/52	5.76 %	3/52	-	-	28.84 %	15/52	9.61 %	5/52	5.76 %	3/52

Percentage (%) and number of specimens (n/N) by taxonomic group used for the carbon and oxygen isotope analyses.

Table S8

group1	group2	n (group1	n (group2)	statistic	p	p.adj
CM	THS	84	62	5.58	0.000	0.000
CM	NL	84	57	3.72	0.000	0.000
CM	DU	84	60	-0.52	0.601	0.601
CM	THM	84	72	7.05	0.000	0.000
THS	NL	62	57	-1.62	0.106	0.133
THS	DU	62	60	-5.65	0.000	0.000
THS	THM	62	72	1.14	0.256	0.284
NL	DU	57	60	-3.93	0.000	0.000
NL	THM	57	72	-3.93	0.000	0.000
DU	THM	60	72	6.98	0.000	0.000

Results of the Post-hoc Dunn's test pair-wise comparisons on $\delta^{13}C_{carbon\ source}$ values between sites (CM, Coc Muoi; THS, Tam Hang South; NL, Nam Lot; DU, Duoi U'Oi; THM, Tam Hay Marklot).

Table S9

group1	group2	n (group1	n (group2)	statistic	p	p.adj
CM	THS	84	62	-2.83	0.005	0.009
CM	NL	84	57	1.42	0.155	0.221
CM	DU	84	60	-1.99	0.047	0.078
CM	THM	84	72	1.22	0.221	0.277
THS	NL	62	57	3.91	0.000	0.001
THS	DU	62	60	0.76	0.445	0.495
THS	THM	62	72	3.87	0.000	0.001
NL	DU	57	60	-3.14	0.0002	0.006
NL	THM	57	72	-0.27	0.788	0.788
DU	THM	60	72	3.04	0.002	0.006

Results of the Post-hoc Dunn's test pair-wise comparisons on $\delta^{18}O$ values between sites (CM, Coc Muoi; THS, Tam Hang South; NL, Nam Lot; DU, Duoi U'Oi; THM, Tam Hay Marklot).

Table S10

		18 – 80 kg	80 – 350 kg	350 – 1000 kg	>1000 kg
**Coc Muoi<br>(148 – 117 ka)**	**Ruminant**	*Muntiacus* sp.	*Rusa unicolor*<br>medium-sized cervid<br>*Capricornis* sp.	*Bos* cf. *sauveli*	
	**Non-ruminant**		*Sus scrofa*	*Megatapirus augustus*<br>*Tapirus indicus*<br>*Dicerorhinus sumatrensis*	*Rhinoceros sondaicus*<br>*Rhinoceros* cf. *unicornis*<br>*Elephas maximus*<br>*Stegodon* sp.
**Tam Hang South<br>(92 – 60 ka)**	**Ruminant**	*Muntiacus muntjak*	*Rusa unicolor*<br>medium-sized cervid<br>*Capricornis sumatraensis*	*Bos* cf. *sauveli*<br>*Bubalus bubalis*	
	**Non-ruminant**		*Sus scrofa*<br>*Sus* cf. *barbatus*	*Megatapirus augustus*<br>*Tapirus indicus* cf. *intermedius*	*Rhinoceros sondaicus*<br>*Rhinoceros unicornis*<br>*Rhinoceros* sp.<br>*Elephas* sp.<br>*Stegodon orientalis*
**Nam Lot<br>(86 – 72 ka)**	**Ruminant**	*Muntiacus muntjak*	*Rusa unicolor*<br>*Capricornis sumatraensis*	*Bos* sp.<br>*Bubalus bubalis*	
	**Non-ruminant**		*Sus scrofa*	*Tapirus* sp.	*Rhinoceros sondaicus*<br>*Rhinoceros unicornis*<br>*Rhinoceros* sp.<br>*Elephas* sp.<br>*Stegodon* cf. *orientalis*
**Duoi U'Oi<br>(70 – 60 ka)**	**Ruminant**	*Muntiacus muntjak*	*Rusa unicolor*<br>*Capricornis sumatraensis*	*Bubalus* cf. *bubalis*	
	**Non-ruminant**		*Sus scrofa*<br>*Sus barbatus*	*Tapirus indicus*<br>*Dicerorhinus sumatrensis*	*Rhinoceros sondaicus*<br>*Rhinoceros unicornis*<br>*Elephas* sp.
**Tam Hay Marklot<br>(38.4 – 13.5 ka)**	**Ruminant**	*Muntiacus* sp.<br>*Axis* cf. *porcinus*<br>*Naemorhedus caudatus*	*Rusa unicolor*<br>*Rucervus eldii*<br>*Capricornis* cf. *sumatraensis*	*Bos* sp.<br>*Bubalus bubalis*	
	**Non-ruminant**		*Sus* sp.<br>*Sus* cf. *barbatus*	*Tapirus* sp.<br>*Dicerorhinus sumatrensis*	*Rhinoceros sondaicus*<br>*Elephas* sp.
**Current faunas**	**Ruminant**	*Muntiacus muntjak*<br>*Muntiacus rooseveltorum*<br>*Naemorhedus caudatus*<br>*Cervus nippon*	*Rusa unicolor*<br>*Rucervus eldii*<br>*Capricornis sumatraensis*	*Bos gaurus*<br>*Bos javanicus*	
	**Non-ruminant**		*Sus scrofa*	*Dicerorhinus sumatrensis*	*Rhinoceros sondaicus*<br>*Elephas maximus*

Lists of taxa by body mass and dietary strategy. The current faunas are those from the studied latitudinal zone in the pre-industrial period (Corbet and Hill, 1992); body size categories are from Faith et al. (2019). No herbivore species smallest than ~20 kg is recorded in the four fossil assemblages. Only species of Tragulidae constitute the current faunas at these latitudes (Corbet and Hill, 1992).

Table S11

group1	group2	n (group1	n (group2)	statistic	p	p.adj
CM	THS	16	3	0.95	0.341	0.426
CM	NL	16	8	4.11	0.000	0.000
CM	DU	16	4	2.91	0.004	0.012
CM	THM	16	34	1.82	0.069	0.136
THS	NL	3	8	1.74	0.082	0.136
THS	DU	3	4	1.34	0.179	0.255
THS	THM	3	34	-0.08	0.936	0.936
NL	DU	8	4	-0.25	0.804	0.893
NL	THM	8	34	-3.12	0.002	0.009
DU	THM	4	34	-2.03	0.042	0.105

Results of the Post-hoc Dunn's test pair-wise comparisons on crown area dimensions of *Rusa unicolor* between sites (CM, Coc Muoi; THS, Tam Hang South; NL, Nam Lot; DU, Duoi U'Oi; THM, Tam Hay Marklot).

Table S12

group1	group2	n (group1	n (group2)	statistic	p	p.adj
CM	THS	13	12	1.75	0.080	0.133
CM	NL	13	4	2.47	0.014	0.034
CM	DU	13	14	-0.88	0.378	0.431
CM	THM	13	18	-0.09	0.929	0.929
THS	NL	12	4	1.23	0.220	0.314
THS	DU	12	14	-2.65	0.008	0.030
THS	THM	12	18	-1.97	0.049	0.098
NL	DU	4	14	-3.09	0.002	0.020
NL	THM	4	18	-2.61	0.009	0.030
DU	THM	14	18	0.86	0.388	0.431

Results of the Post-hoc Dunn's test pair-wise comparisons on crown area dimensions of *Sus scrofa* between sites (CM, Coc Muoi; THS, Tam Hang South; NL, Nam Lot; DU, Duoi U'Oi; THM, Tam Hay Marklot).

Table S13

	Rusa unicolor		*Muntiacus* sp.		*Capricornis sumatraensis*		*Sus scrofa*		*Macaca* sp.	
	**p3**	**$\delta^{13}C$**	**m3**	**$\delta^{13}C$**	**m3**	**$\delta^{13}C$**	**p3**	**$\delta^{13}C$**	**m3**	**$\delta^{13}C$**
**Coc Muoi**	16	6	4	7	2	5	13	6	2	5
**Tam Hang South**	3	8	8	4	1	3	12	11	7	6
**Nam Lot**	8	5	1	3	3	2	4	5	2	4
**Duoi U'Oi**	4	6	12	5	4	3	14	5	15	5
**Tam Hay Marklot**	34	5	17	5	8	5	18	6	1	5
**Total**	65	30	42	24	18	18	61	33	27	25

Taxon, tooth type, and number of specimens used for the crown area measurements, and number of specimens used for the $\delta^{13}C$ isotope analysis (data of the Figure 5).

Table S14

	**$\delta^{13}C_{carbon\ source}$ < –27.2 ‰**		**$\delta^{13}C_{carbon\ source}$ >–27.2 ‰ and < –21.3 ‰**		**$\delta^{13}C_{carbon\ source}$ > –21.3 ‰ and < –15.3 ‰**		**$\delta^{13}C_{carbon\ source}$ > –15.3‰**	
**Coc Muoi**	65.48 %	55/84	33.33 %	28/84	1.19 %	1/84	-	0/84
**Tam Hang South**	27.42 %	17/62	58.06 %	36/62	11.29 %	7/62	3.23 %	2/62
**Nam Lot**	42.11 %	24/57	49.12 %	28/57	5.26 %	3/57	3.51 %	2/57
**Duoi U'Oi**	73.33 %	44/60	25.00 %	15/60	-	0/60	1.67 %	1/60
**Tam Hay Marklot**	26.39 %	19/72	43.06 %	31/72	18.05 %	13/72	12.50 %	9/72
**Boh Dambang**	9.61 %	5/52	32.69 %	17/52	38.46 %	20/52	19.23 %	10/52

Percentage (%) and number of specimens (n/N) in the five faunas compared with the data from the fauna of Boh Dambang (Bacon et al., 2018c), according to the distribution of $\delta^{13}C_{carbon\ source}$ values (‰ VPDB).

References

Bacon, A.-M. *et al.* The Late Pleistocene Duoi U'Oi cave in northern Vietnam: palaeontology, sedimentology, taphonomy, palaeoenvironments. *Quaternary Science Reviews* 27, 1627-1654 (2008).

Bacon, A.-M. *et al.* Late Pleistocene mammalian assemblages of Southeast Asia: new dating, mortality profiles and evolution of the predator-prey relationships in an environmental context. *Palaeogeography, Palaeoclimatology, Palaeoecology* 422, 101-127 (2015).

Bacon, A.-M. *et al.* A rhinocerotid-dominated megafauna at the MIS6-5 transition: The late Middle Pleistocene Coc Muoi assemblage, Lang Son province, Vietnam. *Quaternary Science Reviews* 186, 123-141 (2018a).

Bacon, A.-M. *et al.* Nam Lot (MIS 5) and Duoi U'Oi (MIS 4) Southeast Asian sites revisited: Zooarchaeological and isotopic evidences. *Palaeogeography, Palaeoclimatology, Palaeoecology* 512, 132-144 (2018b).

Bacon, A.-M. *et al.* Testing the savannah corridor hypothesis during MIS2: The Boh Dambang hyena site in southern Cambodia. *Quaternary International* 464, 417-439 (2018c).

Balasse, M. Reconstructing dietary and environmental history from enamel isotopic analysis: time resolution of intra-tooth sequential sampling. *International Journal of Osteoarchaeology* 12, 155-165 (2002).

Bocherens, H. Preservation of Isotopic Signals (^{13}C, ^{15}N) in Pleistocene Mammals. *Biogeochemical approaches to paleodietary analysis*, 65-88 (2002).

Bocherens, H., *et al.* Niche partitioning between two sympatric genetically distinct cave bears (*Ursus spelaeus* and *Ursus ingressus*) and brown bear (*Ursus arctos*) from Austria: isotopic evidence from fossil bones. *Quaternary International* 245, 238-248 (2011).

Bocherens, H. *et al.* Flexibility of diet and habitat in Pleistocene South Asian mammals: Implications for the fate of the giant fossil ape *Gigantopithecus*. *Quaternary International* 434, 148–155 (2017).

Bourgon, N. *et al.* Zinc isotopes in Late Pleistocene fossil teeth from a Southeast Asian cave setting preserve paleodietary information. *Proceedings of National Academy of Sciences* 117, 4675-4681 (2020).

Brain, C. K. The hunters and the hunted? An introduction to African cave taphonomy (The University of Chicago press, Chicago and London, 1981).

Cappellini, E., *et al.* Early Pleistocene enamel proteome from Dmanisi resolves *Stephanorhinus* phylogeny. *Nature* 574, 103-107 (2019).

Cerling, T.E. & Harris, J.M. Carbon Isotope Fractionation between Diet and Bioapatite in Ungulate Mammals and Implications for Ecological and Paleoecological Studies. *Oecologia* 120, 347-363 (1999).

Cerling, T.E., Hart, J.A. & Hart. T.B. Stable isotope ecology in the Ituri Forest. *Oecologia* 138, 5-12 (2004).

Charif, D. & Lobry, J.R. SeqinR 1.0-2: A Contributed Package to the R Project for Statistical Computing Devoted to Biological Sequences Retrieval and Analysis. *Structural Approaches to Sequence Evolution,* 207-232 (2007).

Clementz, M.T., Fox-Dobbs, K., Wheatley, P.V., Koch, P.L. & Doak, D.F. Revisiting old bones: coupled carbon isotope analysis of bioapatite and collagen as an ecological and palaeoecological tool. *Geological Journal* 44, 605-620 (2009).

Corbet, G.B. & Hill, J.E. The mammals of the Indomalayan region. Natural History Museum publications. Oxford University Press (1992).

DeNiro, M.J. & Epstein, S. Influence of diet on the distribution of carbon isotopes in animals. *Geochimica et Cosmochimica Acta* 42, 495-506 (1978).

Duringer, P., Bacon, A.-M., Sayavongkhamdy, T. & Nguyen Thi Kim Thuy. Karst development, breccias history, and mammalian assemblages in Southeast Asia: A brief review. *Comptes Rendus Palevol* 11, 133-157 (2012).

Esposito, M. U-series dating of fossil teeth and carbonates from Snake cave, Thailand. *Journal of Archaeological Science* 29, 341-349 (2002).

Esposito, M., Chaimanee, Y., Jaeger, J.-J. & Reyss, J.-L. Datation des concrétions carbonatées de la "Grotte du serpent" (Thaïlande) par la méthode Th/U. *Comptes Rendus de l'Académie des Sciences*, Paris 326, 603-608 (1998).

Faith, J. T., Rowan, J. & Du, A. Early hominins evolved within non-analog ecosystems. *Proceedings of the National Academy of Sciences* 116, 21478-21483 (2019).

Fox-Dobbs, K., Wheatley, P.V. & Koch, P.L. Carnivore specific bone bioapatite and collagen carbon isotope fractionations: Case studies of modern and fossil grey wolf populations ». AGU Fall Meeting Abstracts 53 (2006).

Friedli, H., Lötscher, H., Oeschger, H., Siegenthaler, U. & Stauffer, B. Ice Core Record of the 13C/12C Ratio of Atmospheric CO_2 in the Past Two Centuries ». *Nature* 324, 237-238 (1986).

Han, H.A.N. *et al.* Distinctive diet-tissue isotopic discrimination factors derived from the exclusive bamboo-eating giant panda. *Integrative zoology* 11*,* 447-456 (2016).

Kohn, M.J. Carbon Isotope Compositions of Terrestrial C3 Plants as Indicators of (Paleo)ecology and (Paleo)climate. *Proceedings of the National Academy of Sciences* 107, 19691-19695 (2010).

Kolde, R. Package 'pheatmap'. https://mran.microsoft.com/snapshot/2017-09-01/web/packages/pheatmap/pheatmap.pdf (2015)

Lee-Thorp, J.A. & van der Merwe, N.J. Carbon isotope analysis of fossil bone apatite. *South African Journal of Science* 83, 712-715 (1987).

Lee-Thorp, J.A., Sealy, J.C. & van der Merwe, N.J. Stable carbon isotope ratio differences between bone collagen and bone apatite, and their relationship to diet. *Journal of Archaeological Science* 16, 585-599 (1989).

Li, D. *et al*. The stable isotope record in cervid tooth enamel from Tantang Cave, Guangxi: Implications for the Quaternary East Asian monsoon. *Quaternary International* 434, 156-162 (2017).

Longinelli, A. Oxygen isotopes in mammal bone phosphate: a new tool for paleohydrological and paleoclimatological research? *Geochimica et Cosmochimica Acta* 48, 385-390 (1984).

Louys, J. & Roberts, P. Environmental drivers of megafauna and hominin extinction in Southeast Asia. *Nature* 586, 402-406 (2020).

Luz, B., Kolodny, Y. & Horowitz, M. Fractionation of oxygen isotopes between mammalian bone-phosphate and environmental drinking water. *Geochimica et Cosmochimica Acta* 48, 1689-1693 (1984).

Ma, J. *et al.* Isotopic evidence of foraging ecology of Asian elephant (*Elephas maximus*) in South China during the Late Pleistocene. *Quaternary International* 443, 160-167 (2017).

Ma, J., Wang, Y., Jin, C., Hu, Y. & Bocherens, H. Ecological flexibility and differential survival of Pleistocene *Stegodon orientalis* and *Elephas maximus* in mainland southeast Asia revealed by stable isotope (C, O) analysis. *Quaternary Science Reviews* 212, 33-44 (2019).

Mackie, M. *et al.* Palaeoproteomic profiling of conservation layers on a 14th Century Italian wall *painting. Angewandte Chemie* 57, 7369-7374 (2018).

Marwick, B. & Gagan, M. K. Late Pleistocene monsoon variability in northwest Thailand: an oxygen isotope sequence from the bivalve *Margaritanopsis laosensis* excavated in Mae Hong Son province. *Quaternary Science Reviews* 30, 3088-3098 (2011).

O'Leary, M.H. Carbon Isotopes in Photosynthesis. *BioScience* 38, 328-336 (1988).

Passey, B.H. *et al.* Carbon isotope fractionation between diet, breath CO2, and bioapatite in different mammals ». *Journal of Archaeological Science* 32, 1459-1470 (2005).

Patole-Edoumba E. & Demeter F. (Dir.). *Pà Hang, la montagne habitée.* Catalogue de l'exposition au Muséum d'histoire naturelle de La Rochelle, *Les Indes Savantes* (2019).

Pushkina, D., Bocherens, H., Chaimanee, Y. & Jaeger, J.-J., 2010. Stable carbon isotope reconstructions of diet and paleoenvironment from the late Middle Pleistocene Snake Cave in Northeastern Thailand. *Naturwissenschaften* 97, 299–309 (2010).

Smith, B.N. & Epstein, S. Two Categories of $^{13}C/^{12}C$ Ratios for Higher Plants 1. *Plant Physiology* 47, 380-384 (1971).

Sponheimer, M. & Lee-Thorp, J.A., 1999. Oxygen Isotopes in Enamel Carbonate and their Ecological Significance. *Journal of Archaeological Science* 26, 723-728 (1999).

Sponheimer, M. *et al.* Isotopic evidence of early hominin diets. *Proceedings of the National Academy of Sciences* 110, 10513-10518 (2013).

Sullivan, C. H. & Krueger, H. W. Carbon isotope analysis of separate chemical phases in modern and fossil bone. *Nature* 292, 333–335 (1981).

Sun, F. *et al.* Paleoecology of Pleistocene mammals and paleoclimatic change in South China: Evidence from stable carbon and oxygen isotopes. *Palaeogeography, Palaeoclimatology, Palaeoecology* 524, 1-12 (2019).

Suraprasit, K. *et al.* Long-term isotope evidence on the diet and habitat breadth of Pleistocene to Holocene caprines in Thailand; Implications for the extirpation and conservation of Himalayan gorals. *Frontiers in Ecology and Evolution* 8, 1-16 (2020).

Suraprasit, K., Jaeger, J.-J., Chaimanee, Y. & Sutcharit, C. Taxonomic reassessment of large mammals from the Pleistocene Homo-bearing site of Tham Wiman Nakin (Northeast Thailand): relevance for faunal patterns in mainland Southeast Asia. Quaternary International. doi.org/10.1016/j.quaint.2020.06.050 (2020).

Tejada-Lara, J.V. *et al.* Body mass predicts isotope enrichment in herbivorous mammals. *Proceedings of the Royal Society B* 285*,* 20181020 (2018).

Tougard, C. Les faunes de grands mammifères du Pléistocène moyen terminal de Thaïlande dans leur cadre phylogénétique, paléoécologique et biochronologique. Thèse de Doctorat, Université de Montpellier II, 175 p (1998).

Tougard, C., Jaeger, J.J., Chaimanee, Y., Suteethorn, V. & Triamwichanon, S. Discovery of a *Homo* sp. tooth associated with a mammalian cave fauna of Late Middle Pleistocene age, Northern Thailand. *Journal of Human Evolution* 35, 47-54 (1998).

Wattanapituksakul, A., Filoux, A., Amphansri, A. & Tumpeesuwan, S. Late Pleistocene caprinae assemblages of Tham Lod Rockshelter (Mae Hong Son Province, Northwest Thailand). *Quaternary International* 493, 212-226 (2018).

Welker, F. Elucidation of cross-species proteomic effects in human and hominin bone proteome identification through a bioinformatics experiment. *BMC Evolutionary Biology* 18: 23 (2018)

Welker, F. *et al.* Enamel proteome shows that *Gigantopithecus* was an early diverging pongine. *Nature* 576, 262-265 (2019).

Welker, F. *et al.* The dental proteome of *Homo antecessor*. *Nature* 580, 235-238 (2020).

Wood, R., *et al.* The effect of grain size on carbonate contaminant removal from tooth enamel: Towards an improved pretreatment for radiocarbon dating. *Quaternary Geochronology* 36, 174-187 (2016).

Wood, R. *et al*. Do weak or strong acids remove carbonate contamination from ancient tooth enamel more effectively? The effect of acid pretreatment on radiocarbon and 13C analyses. Radiocarbon 63, 935-952 (2021).

Annex S1. Faunal lists from main sites investigated in the present study (Coc Muoi, Tam Hang South and Duoi U'Oi) with associated $\delta^{13}C_{apatite}$, $\delta^{13}C_{carbon\ source}$ and $\delta^{18}O_{apatite}$values (‰ VPDB), as well as body mass and $\delta^{13}C$ (‰ VPDB) Enrichment Factor used to obtain $\delta^{13}C_{carbon\ source}$.

Number	Country	Site	Taxon	Body Mass (kg)	$\delta^{13}C_{apatite}$ (‰ VPDB)	$\delta^{13}C$ (‰) Enrichment Factor	$\delta^{13}C_{carbon\ source}$ (‰ VPDB)	$\delta^{18}O_{apatite}$ (‰ VPDB)
DU876	Vietnam	Duoi U'Oi	*Sus scrofa*	137	-14.2	13.19	-27.4	-5.1
DU890	Vietnam	Duoi U'Oi	*Sus scrofa*	137	-12.8	13.19	-26.0	-8.3
DU905	Vietnam	Duoi U'Oi	*Sus scrofa*	137	-13.7	13.19	-26.9	-7.1
DU906	Vietnam	Duoi U'Oi	*Sus scrofa*	137	-15	13.19	-28.2	-6
DU913	Vietnam	Duoi U'Oi	*Sus scrofa*	137	-14.7	13.19	-27.9	-6.2
DU546	Vietnam	Duoi U'Oi	*Rusa unicolor*	220	-16.6	13.59	-30.2	-7.1
DU557	Vietnam	Duoi U'Oi	*Rusa unicolor*	220	-19.6	13.59	-33.2	-6.1
DU567	Vietnam	Duoi U'Oi	*Rusa unicolor*	220	-18.1	13.59	-31.7	-6.1
DU608	Vietnam	Duoi U'Oi	*Rusa unicolor*	220	-18.1	13.59	-31.7	-7
DU990	Vietnam	Duoi U'Oi	*Rusa unicolor*	220	-18.3	13.59	-31.9	-5.7
DU1087	Vietnam	Duoi U'Oi	*Rusa unicolor*	220	-17.7	13.59	-31.3	-8.5
DU437	Vietnam	Duoi U'Oi	*Muntiacus muntjak*	24	-13.2	12.17	-25.4	-9.3
DU461	Vietnam	Duoi U'Oi	*Muntiacus muntjak*	24	-14.8	12.17	-27.0	-7.7
DU511	Vietnam	Duoi U'Oi	*Muntiacus muntjak*	24	-14.6	12.17	-26.8	-5.1
DU433	Vietnam	Duoi U'Oi	*Muntiacus muntjak*	24	-13.8	12.17	-26.0	-7.8
DU392	Vietnam	Duoi U'Oi	*Muntiacus muntjak*	24	-13	12.17	-25.2	-6.5
DU574	Vietnam	Duoi U'Oi	*Capricornis sumatraensis*	112	-15.8	13.14	-28.9	-7.1
DU613	Vietnam	Duoi U'Oi	*Capricornis sumatraensis*	112	-17.4	13.14	-30.5	-7.5
DU538	Vietnam	Duoi U'Oi	*Capricornis sumatraensis*	112	-14.5	13.14	-27.6	-6.1
DU992	Vietnam	Duoi U'Oi	Bovidae	875	-19.8	14.57	-34.4	-7.1
DU983	Vietnam	Duoi U'Oi	Bovidae	875	-12.1	14.57	-26.7	-5.2
DU984	Vietnam	Duoi U'Oi	Bovidae	875	-0.6	14.57	-15.2	-4.7
DU560	Vietnam	Duoi U'Oi	Bovidae	875	-19.3	14.57	-33.9	-2.5
	Vietnam	Duoi U'Oi	*Hystrix brachyura*	12	-16.7	12.18	-28.9	-8
	Vietnam	Duoi U'Oi	*Hystrix brachyura*	12	-12.6	12.18	-24.8	-6.7
	Vietnam	Duoi U'Oi	*Hystrix brachyura*	12	-16.26	12.18	-28.4	-9.71
	Vietnam	Duoi U'Oi	*Hystrix brachyura*	12	-12.8	12.18	-25.0	-6.39
	Vietnam	Duoi U'Oi	*Hystrix brachyura*	12	-17.08	12.18	-29.3	-8.68
	Vietnam	Duoi U'Oi	*Hystrix brachyura*	12	-13.7	12.18	-25.9	-6.6
	Vietnam	Duoi U'Oi	*Hystrix brachyura*	12	-17.3	12.18	-29.5	-5.1
DU7	Vietnam	Duoi U'Oi	*Hystrix brachyura*	12	-14.7	12.18	-26.9	-8.9
DU728	Vietnam	Duoi U'Oi	*Ursus* sp.	100	-15	13.3	-28.3	-6.5
DU729	Vietnam	Duoi U'Oi	*Ursus* sp.	100	-14.9	13.3	-28.2	-9.3
DU1152	Vietnam	Duoi U'Oi	*Cuon alpinus*	15	-14.2	13.39	-27.6	-7.3
DU1153	Vietnam	Duoi U'Oi	*Cuon alpinus*	15	-14.9	13.39	-28.3	-6.7

DU77	Vietnam	Duoi U'Oi	*Cuon alpinus*	15	-14.5	13.39	-27.9	-7.4
DU68	Vietnam	Duoi U'Oi	*Panthera pardus*	41	-14.2	13.81	-28.0	-5.2
DU86	Vietnam	Duoi U'Oi	*Panthera pardus*	41	-13.9	13.81	-27.7	-5.2
DU707	Vietnam	Duoi U'Oi	*Panthera tigris*	212	-15.5	14.53	-30.0	-5.4
DU326	Vietnam	Duoi U'Oi	*Macaca* sp.	6	-11.9	11.91	-23.8	-6.2
DU331	Vietnam	Duoi U'Oi	*Macaca* sp.	6	-15.6	11.91	-27.5	-5.1
DU339	Vietnam	Duoi U'Oi	*Macaca* sp.	6	-14.4	11.91	-26.3	-4.4
DU343	Vietnam	Duoi U'Oi	*Macaca* sp.	6	-15.4	11.91	-27.3	-5
DU322	Vietnam	Duoi U'Oi	*Macaca* sp.	6	-16	11.91	-27.9	-6.3
DU32	Vietnam	Duoi U'Oi	*Rhinoceros unicornis*	2250	-17.1	14.4	-31.5	-7.2
DU26	Vietnam	Duoi U'Oi	*Rhinoceros unicornis*	2250	-16.1	14.4	-30.5	-6.4
DU28	Vietnam	Duoi U'Oi	*Rhinoceros unicornis*	2250	-16.2	14.4	-30.6	-6.6
DU30	Vietnam	Duoi U'Oi	*Rhinoceros sondaicus*	2250	-15.7	14.4	-30.1	-6.4
DU31	Vietnam	Duoi U'Oi	*Rhinoceros sondaicus*	2250	-15.3	14.4	-29.7	-4.9
DU38	Vietnam	Duoi U'Oi	*Rhinoceros sondaicus*	2250	-16.6	14.4	-31.0	-7
DU27	Vietnam	Duoi U'Oi	*Dicerorhinus sumatrensis*	950	-17.5	14	-31.5	-7.9
DU24	Vietnam	Duoi U'Oi	*Dicerorhinus sumatrensis*	950	-16.4	14	-30.4	-6.6
DU47	Vietnam	Duoi U'Oi	*Tapirus indicus*	300	-17.3	13.5	-30.8	-7.9
DU43	Vietnam	Duoi U'Oi	*Tapirus indicus*	300	-20.1	13.5	-33.6	-6.9
DU53	Vietnam	Duoi U'Oi	*Tapirus indicus*	300	-19.1	13.5	-32.6	-8.3
DU1021	Vietnam	Duoi U'Oi	*Pongo pygmaeus*	55	-14.6	12.78	-27.4	-6
DU1022	Vietnam	Duoi U'Oi	*Pongo pygmaeus*	55	-14.5	12.78	-27.3	-5.1
DU1023	Vietnam	Duoi U'Oi	*Pongo pygmaeus*	55	-14.3	12.17	-26.5	-4.9
DU634	Vietnam	Duoi U'Oi	*Elephas* sp.	4250	-16.9	14.69	-31.6	-7
DU-	Vietnam	Duoi U'Oi	*Elephas* sp.	4250	-15.9	14.69	-30.6	-6.8
CM169	Vietnam	Coc Muoi	*Muntiacus* sp.	24	-14.2	12.17	-26.4	-7.1
CM307	Vietnam	Coc Muoi	*Muntiacus* sp.	24	-14.5	12.17	-26.7	-6.5
CM357	Vietnam	Coc Muoi	*Muntiacus* sp.	24	-13.5	12.17	-25.7	-5.7
Cm313	Vietnam	Coc Muoi	*Muntiacus* sp.	24	-14	12.17	-26.2	-5.5
CM175	Vietnam	Coc Muoi	*Muntiacus* sp.	24	-13.9	12.17	-26.1	-6.9
CM176	Vietnam	Coc Muoi	*Muntiacus* sp.	24	-13.2	12.17	-25.4	-4.4
CM324	Vietnam	Coc Muoi	*Muntiacus* sp.	24	-16.5	12.17	-28.7	-6.2
CM450	Vietnam	Coc Muoi	*Sus scrofa*	137	-14.5	13.19	-27.7	-6.2
CM676	Vietnam	Coc Muoi	*Sus scrofa*	137	-13.9	13.19	-27.1	-6.9
CM677	Vietnam	Coc Muoi	*Sus scrofa*	137	-13.8	13.19	-27.0	-6.5
CM678	Vietnam	Coc Muoi	*Sus scrofa*	137	-13.1	13.19	-26.3	-6.4
CM748	Vietnam	Coc Muoi	*Sus scrofa*	137	-13.7	13.19	-26.9	-7.2
CM746	Vietnam	Coc Muoi	*Sus scrofa*	137	-13.9	13.19	-27.1	-6.2
CM543	Vietnam	Coc Muoi	*Ailuropoda melanoleuca*	92	-17.4	10.51	-27.9	-4.2
CM544	Vietnam	Coc Muoi	*Ailuropoda melanoleuca*	92	-17.7	10.51	-28.2	-5.6
CM564	Vietnam	Coc Muoi	*Ailuropoda melanoleuca*	92	-18.5	10.51	-29.0	-6.7

CM415	Vietnam	Coc Muoi	*Ursus* sp. (? *thibetanus*)	100	-14.5	13.3	-27.8	-5.8
CM518	Vietnam	Coc Muoi	*Ursus thibetanus*	100	-17	13.3	-30.3	-6.9
CM519	Vietnam	Coc Muoi	*Ursus thibetanus*	100	-13.4	13.3	-26.7	-6.4
CM521	Vietnam	Coc Muoi	*Ursus* sp. (? *thibetanus*)	100	-14.4	13.3	-27.7	-8.5
CM525	Vietnam	Coc Muoi	*Ursus thibetanus*	100	-14.1	13.3	-27.4	-6.8
CM508	Vietnam	Coc Muoi	*Pongo devosi*	55	-15.3	12.78	-28.1	-4
CM579	Vietnam	Coc Muoi	*Pongo devosi*	55	-15	12.78	-27.8	-4.7
CM551	Vietnam	Coc Muoi	*Pongo devosi*	55	-14.8	12.78	-27.6	-4.8
CM553	Vietnam	Coc Muoi	*Pongo devosi*	55	-15	12.78	-27.8	-5
CM509	Vietnam	Coc Muoi	*Macaca* sp.	6	-14	11.91	-25.9	-4.7
CM577	Vietnam	Coc Muoi	*Macaca* sp.	6	-13.8	11.91	-25.7	-7.4
CM614	Vietnam	Coc Muoi	*Macaca* sp.	6	-14.2	11.91	-26.1	-5.9
CM618	Vietnam	Coc Muoi	*Macaca* sp.	6	-15.1	11.91	-27.0	-5.2
CM555	Vietnam	Coc Muoi	*Macaca* sp.	6	-15.4	11.91	-27.3	-4.7
CM534	Vietnam	Coc Muoi	*Panthera tigris*	212	-16.2	14.53	-30.7	-7.5
CM566	Vietnam	Coc Muoi	*Panthera tigris*	212	-14.6	14.53	-29.1	-5.4
CM632	Vietnam	Coc Muoi	*Panthera tigris*	212	-16	14.53	-30.5	-8.6
CM571	Vietnam	Coc Muoi	*Cuon alpinus*	15	-14.3	13.39	-27.7	-6.7
CM535	Vietnam	Coc Muoi	Small-sized Felidae	18	-14.5	13.46	-28.0	-8.6
CM536	Vietnam	Coc Muoi	Small-sized Felidae	18	-13.2	13.46	-26.7	-6.2
CM421	Vietnam	Coc Muoi	*Hystrix* sp.	12	-14.8	12.18	-27.0	-8.1
CM472	Vietnam	Coc Muoi	*Hystrix* sp.	12	-15.8	12.18	-28.0	-3.9
CM902	Vietnam	Coc Muoi	*Hystrix* sp.	12	-16	12.18	-28.2	-7.2
CM903	Vietnam	Coc Muoi	*Hystrix* sp.	12	-14.5	12.18	-26.7	-7.5
CM904	Vietnam	Coc Muoi	*Hystrix* sp.	12	-16	12.18	-28.2	-4.1
CM910	Vietnam	Coc Muoi	*Hystrix* sp.	12	-13.6	12.18	-25.8	-8.6
CM911	Vietnam	Coc Muoi	*Hystrix* sp.	12	-13.7	12.18	-25.9	-4.6
CM954	Vietnam	Coc Muoi	*Hystrix* sp.	12	-14.7	12.18	-26.9	-4.9
CM983	Vietnam	Coc Muoi	*Atherurus* sp.	12	-14.6	12.18	-26.8	-5.8
CM984	Vietnam	Coc Muoi	*Atherurus* sp.	12	-14.1	12.18	-26.3	-4.6
CM75	Vietnam	Coc Muoi	*Rusa unicolor*	220	-17.5	13.59	-31.1	-6.8
CM244	Vietnam	Coc Muoi	*Rusa unicolor*	220	-13.4	13.59	-27.0	-6.9
CM245	Vietnam	Coc Muoi	*Rusa unicolor*	220	-15.2	13.59	-28.8	-6.5
CM246	Vietnam	Coc Muoi	*Rusa unicolor*	220	-14	13.59	-27.6	-5.8
CM247	Vietnam	Coc Muoi	*Rusa unicolor*	220	-17	13.59	-30.6	-7.8
CM249	Vietnam	Coc Muoi	*Rusa unicolor*	220	-16.3	13.59	-29.9	-7.3
CM287	Vietnam	Coc Muoi	*Capricornis* sp.	112	-15	13.14	-28.1	-5.9
CM173	Vietnam	Coc Muoi	*Capricornis* sp.	112	-17.7	13.14	-30.8	-5.1
CM286	Vietnam	Coc Muoi	*Capricornis* sp.	112	-17.9	13.14	-31.0	-7.6
CM147	Vietnam	Coc Muoi	*Capricornis* sp.	112	-14.5	13.14	-27.6	-4.9
CM149	Vietnam	Coc Muoi	*Capricornis* sp.	112	-17.1	13.14	-30.2	-5.5

CM370	Vietnam	Coc Muoi	*Bos* cf. *sauveli*	800	-14.8	14.5	-29.3	-5.8
CM225	Vietnam	Coc Muoi	*Bos* cf. *sauveli*	800	-8.9	14.5	-23.4	-6.6
CM226	Vietnam	Coc Muoi	*Bos* cf. *sauveli*	800	-11.1	14.5	-25.6	-5.9
CM227	Vietnam	Coc Muoi	*Bos* cf. *sauveli*	800	-11.3	14.5	-25.8	-6.9
CM46	Vietnam	Coc Muoi	*Bos* cf. *sauveli*	800	-3.6	14.5	-18.1	-4.1
CM152a	Vietnam	Coc Muoi	Bovidae (?*Bos sauveli*)	875	-13.4	14.57	-28.0	-5.9
CM1067	Vietnam	Coc Muoi	*Rhinoceros sondaicus*	1750	-17	14.28	-31.3	-5.4
CM1122	Vietnam	Coc Muoi	*Rhinoceros sondaicus*	1750	-16.6	14.28	-30.9	-4.9
CM1120	Vietnam	Coc Muoi	*Rhinoceros sondaicus*	1750	-15.6	14.28	-29.9	-5.8
CM1068	Vietnam	Coc Muoi	*Rhinoceros sondaicus*	1750	-15.5	14.28	-29.8	-5.8
CM1229	Vietnam	Coc Muoi	*Rhinoceros sondaicus*	1750	-16.3	14.28	-30.6	-4.7
CM1137	Vietnam	Coc Muoi	*Rhinoceros sondaicus*	1750	-17	14.28	-31.3	-7.8
CM1339	Vietnam	Coc Muoi	*Rhinoceros sondaicus*	1750	-16.5	14.28	-30.8	-5.4
CM998	Vietnam	Coc Muoi	*Rhinoceros sondaicus*	1750	-16.4	14.28	-30.7	-7.1
CM1151	Vietnam	Coc Muoi	*Rhinoceros unicornis*	2250	-16.3	14.4	-30.7	-5.8
CM1278	Vietnam	Coc Muoi	*Rhinoceros unicornis*	2250	-16.8	14.4	-31.2	-6.2
CM1035	Vietnam	Coc Muoi	*Dicerorhinus sumatrensis*	950	-15.8	14	-29.8	-6.4
CM1096	Vietnam	Coc Muoi	*Dicerorhinus sumatrensis*	950	-15.6	14	-29.6	-5
CM1349	Vietnam	Coc Muoi	*Megatapirus augustus*	500	-20.1	13.72	-33.8	-5.9
CM514	Vietnam	Coc Muoi	*Megatapirus augustus*	500	-18.7	13.72	-32.4	-5.5
CM515	Vietnam	Coc Muoi	*Tapirus indicus*	300	-17.8	13.5	-31.3	-6.2
CM516	Vietnam	Coc Muoi	*Tapirus indicus*	300	-18.3	13.5	-31.8	-6.8
CM1351	Vietnam	Coc Muoi	*Tapirus indicus*	300	-16.4	13.5	-29.9	-6.6
CM726	Vietnam	Coc Muoi	*Stegodon* sp.	4000	-14.2	14.66	-28.9	-7.9
CM637	Vietnam	Coc Muoi	*Stegodon* sp.	4000	-14.7	14.66	-29.4	-9.8
CM728	Vietnam	Coc Muoi	*Elephas maximus*	4250	-18.3	14.69	-33.0	-5.8
420	Vietnam	Coc Muoi	*Elephas maximus*	4250	-15.8	14.69	-30.5	-6
TH-860	Laos	Tam Hang South	*Cuon alpinus* cf. *antiquus*	15	-12.9	13.39	-26.3	-6.8
TH-861	Laos	Tam Hang South	*Cuon alpinus* cf. *antiquus*	15	-14.4	13.39	-27.8	-2.9
TH-870	Laos	Tam Hang South	*Cuon alpinus* cf. *antiquus*	15	-13	13.39	-26.4	-6.3
TH-539	Laos	Tam Hang South	*Capricornis sumatraensis*	112	-6.8	13.14	-19.9	-4.3
TH-767	Laos	Tam Hang South	*Capricornis sumatraensis*	112	-4.9	13.14	-18.0	-2.8
TH-768	Laos	Tam Hang South	*Capricornis sumatraensis*	112	-13.7	13.14	-26.8	-5.7
TH-873	Laos	Tam Hang South	*Helarctos malayanus*	50	-12.6	13.3	-25.9	-5.9
TH-877	Laos	Tam Hang South	*Helarctos malayanus*	50	-12.5	13.3	-25.8	-7.2
TH-H1	Laos	Tam Hang South	*Hystrix brachyura*	12	-13.7	12.18	-25.9	-5.3
TH-H2	Laos	Tam Hang South	*Hystrix brachyura*	12	-9.9	12.18	-22.1	-9.4
TH-H3	Laos	Tam Hang South	*Hystrix brachyura*	12	-10.2	12.18	-22.4	-5.4
TH-H4	Laos	Tam Hang South	*Hystrix brachyura*	12	-8.9	12.18	-21.1	-8.9
TH-H5	Laos	Tam Hang South	*Hystrix brachyura*	12	-14.7	12.18	-26.9	-6.7
TH-H6	Laos	Tam Hang South	*Hystrix brachyura*	12	-14	12.18	-26.2	-6.6

TH-789	Laos	Tam Hang South	*Bubalus bubalis*	1000	-2.9	14.66	-17.6	-6.7
TH-791	Laos	Tam Hang South	*Bubalus bubalis*	1000	-5	14.66	-19.7	-6.8
TH-455	Laos	Tam Hang South	*Bos* cf. *sauveli*	800	1.8	14.5	-12.7	-5.7
TH-458	Laos	Tam Hang South	*Bos* cf. *sauveli*	800	-10.9	14.5	-25.4	-8.2
TH-459	Laos	Tam Hang South	*Bos* cf. *sauveli*	800	-11.8	14.5	-26.3	-7.3
TH-546	Laos	Tam Hang South	*Bos* cf. *sauveli*	800	-14.9	14.5	-29.4	-6.4
TH-573	Laos	Tam Hang South	*Bos* cf. *sauveli*	800	-9.9	14.5	-24.4	-7.1
TH-790	Laos	Tam Hang South	*Bos* cf. *sauveli*	800	-12.9	14.5	-27.4	-8.2
TH-799	Laos	Tam Hang South	*Bos* cf. *sauveli*	800	-2.8	14.5	-17.3	-6.3
TH-72	Laos	Tam Hang South	*Macaca* sp.	6	-14	11.91	-25.9	-5.7
TH-73	Laos	Tam Hang South	*Macaca* sp.	6	-13.4	11.91	-25.3	-5
TH-74	Laos	Tam Hang South	*Macaca* sp.	6	-14.1	11.91	-26.0	-5
TH-75	Laos	Tam Hang South	*Macaca* sp.	6	-14	11.91	-25.9	-5.3
TH-76	Laos	Tam Hang South	*Macaca* sp.	6	-13.5	11.91	-25.4	-5.1
TH-79	Laos	Tam Hang South	*Macaca* sp.	6	-14.2	11.91	-26.1	-5.3
TH-593	Laos	Tam Hang South	*Megatapirus augustus*	500	-15.8	13.72	-29.5	-7.5
TH-213	Laos	Tam Hang South	*Muntiacus muntjak*	24	-13.2	12.17	-25.4	-6.8
TH-215	Laos	Tam Hang South	*Muntiacus muntjak*	24	-12.6	12.17	-24.8	-7.5
TH-216	Laos	Tam Hang South	*Muntiacus muntjak*	24	-12.8	12.17	-25.0	-7.7
TH-219	Laos	Tam Hang South	*Muntiacus muntjak*	24	-13.3	12.17	-25.5	-8.1
TH-130	Laos	Tam Hang South	*Panthera tigris*	212	-14.1	14.53	-28.6	-7.8
TH-132	Laos	Tam Hang South	*Panthera tigris*	212	-14	14.53	-28.5	-7.4
TH-376	Laos	Tam Hang South	*Rhinoceros* spp.	2000	-13.5	14.34	-27.8	-6.8
TH-379	Laos	Tam Hang South	*Rhinoceros* spp.	2000	-13.3	14.34	-27.6	-7
TH-576	Laos	Tam Hang South	*Rhinoceros* spp.	2000	-14.4	14.34	-28.7	-7.7
TH-445	Laos	Tam Hang South	*Rusa unicolor*	220	-14.5	13.59	-28.1	-5
TH-469	Laos	Tam Hang South	*Rusa unicolor*	220	-12.7	13.59	-26.3	-6.8
TH-499	Laos	Tam Hang South	*Rusa unicolor*	220	-4.8	13.59	-18.4	-8.2
TH-549	Laos	Tam Hang South	*Rusa unicolor*	220	-15.8	13.59	-29.4	-6.1
TH-555	Laos	Tam Hang South	*Rusa unicolor*	220	-16.2	13.59	-29.8	-6.7
TH-717	Laos	Tam Hang South	*Rusa unicolor*	220	-15	13.59	-28.6	-8.8
TH-719	Laos	Tam Hang South	*Rusa unicolor*	220	-13.5	13.59	-27.1	-6.5
TH-748	Laos	Tam Hang South	*Rusa unicolor*	220	-13	13.59	-26.6	-8.6
TH-390-2	Laos	Tam Hang South	*Sus scrofa*	137	-12.7	13.19	-25.9	-8.6
TH-410	Laos	Tam Hang South	*Sus scrofa*	137	-12.6	13.19	-25.8	-8.2
TH-420	Laos	Tam Hang South	*Sus scrofa*	137	-1.4	13.19	-14.6	-6.3
TH-427	Laos	Tam Hang South	*Sus scrofa*	137	-12	13.19	-25.2	-6.2
TH-430	Laos	Tam Hang South	*Sus scrofa*	137	-11.8	13.19	-25.0	-8.5
TH-431	Laos	Tam Hang South	*Sus scrofa*	137	-12.3	13.19	-25.5	-6.7
TH-433	Laos	Tam Hang South	*Sus scrofa*	137	-12.7	13.19	-25.9	-8.9
TH-637	Laos	Tam Hang South	*Sus scrofa*	137	-13.2	13.19	-26.4	-7.8

TH-642	Laos	Tam Hang South	*Sus scrofa*	137	-11.2	13.19	-24.4	-5.3
TH-645-2	Laos	Tam Hang South	*Sus scrofa*	137	-12.3	13.19	-25.5	-8.1
TH-646	Laos	Tam Hang South	*Sus scrofa*	137	-12.7	13.19	-25.9	-8.5
TH-371	Laos	Tam Hang South	*Tapirus indicus* cf. *intermedius*	300	-16.5	13.5	-30.0	-6.5
TH-378	Laos	Tam Hang South	*Tapirus indicus* cf. *intermedius*	300	-16.3	13.5	-29.8	-5.6
TH-129	Laos	Tam Hang South	*Ursus thibetanus* cf. *kokeni*	100	-14.4	13.3	-27.7	-8.5
TH-139	Laos	Tam Hang South	*Ursus thibetanus* cf. *kokeni*	100	-14.4	13.3	-27.7	-6.1

Annex S2. Faunal lists from already-published sites (Bacon et al., 2018b, Bourgon et al. 2020) of the same latitudinal band used as comparison in the present study (Nam Lot and Tam Hay Marklot), with associated $\delta^{13}C_{apatite}$, $\delta^{13}C_{carbon\ source}$ and $\delta^{18}O_{apatite}$values, as well as body mass and $\delta^{13}C$ (‰) Enrichment Factor used to obtain $\delta^{13}C_{carbon\ source}$. (*) The incisor NL 433 has been identified by using palaeoproteomics.

Number	Country	Site	Taxon	Body Mass (kg)	$\delta^{13}C_{apatite}$ (‰ VPDB)	$\delta^{13}C$ (‰) Enrichment Factor	$\delta^{13}C_{carbon\ source}$ (‰ VPDB)	$\delta^{18}O_{apatite}$ (‰ VPDB)
NL-8	Laos	Nam Lot	*Capricornis sumatraensis*	112	-14.6	13.14	-27.7	-4.1
NL-9	Laos	Nam Lot	*Capricornis sumatraensis*	112	-13	13.14	-26.1	-3.3
NL-17	Laos	Nam Lot	*Rusa unicolor*	220	-12.9	13.59	-26.5	-8.7
NL-19	Laos	Nam Lot	*Rusa unicolor*	220	-7.5	13.59	-21.1	-5.8
NL-29	Laos	Nam Lot	*Rusa unicolor*	220	-14.1	13.59	-27.7	-6.7
NL-22	Laos	Nam Lot	*Rusa unicolor*	220	-0.9	13.59	-14.5	-5.5
NL-24	Laos	Nam Lot	*Rusa unicolor*	220	-2.7	13.59	-16.3	-6.1
NL-63-1	Laos	Nam Lot	*Muntiacus muntjak*	24	-13.4	12.17	-25.6	-6.4
NL-65-1	Laos	Nam Lot	*Muntiacus muntjak*	24	-13.8	12.17	-26.0	-7.3
NL-69	Laos	Nam Lot	*Muntiacus muntjak*	24	-13.9	12.17	-26.1	-3.3
NL-116	Laos	Nam Lot	Bovidae indet.	875	-9.8	14.57	-24.4	-5.9
NL-117	Laos	Nam Lot	Bovidae indet.	875	0.3	14.57	-14.3	-4.9
NL-125	Laos	Nam Lot	Bovidae indet.	875	-13.5	14.57	-28.1	-4.7
NL-161	Laos	Nam Lot	Rhinocerotidae indet.	1633	-13.3	14.25	-27.6	-5.9
NL-162	Laos	Nam Lot	Rhinocerotidae indet.	1633	-13.1	14.25	-27.4	-2.5
NL-254-1-1	Laos	Nam Lot	Rhinocerotidae indet.	1633	-12.7	14.25	-27.0	-6.7
NL-256-1	Laos	Nam Lot	Rhinocerotidae indet.	1633	-15.1	14.25	-29.4	-6.6
NL-256-2	Laos	Nam Lot	Rhinocerotidae indet.	1633	-16.3	14.25	-30.6	-4.9
NL-256-3	Laos	Nam Lot	Rhinocerotidae indet.	1633	-15.1	14.25	-29.4	-6.7
NL-139	Laos	Nam Lot	*Bubalus bubalis*	1000	-4.7	14.66	-19.4	-6.5
NL-143	Laos	Nam Lot	*Bubalus bubalis*	1000	-9.5	14.66	-24.2	-4.4
NL-186	Laos	Nam Lot	*Ailuropoda melanoleuca*	92	-14.5	10.51	-25.0	-6.2
NL-277	Laos	Nam Lot	*Ailuropoda melanoleuca*	92	-14.9	10.51	-25.4	-4.2
NL-162	Laos	Nam Lot	*Sus* sp.	137	-13.7	13.19	-26.9	-5.7
NL-208	Laos	Nam Lot	*Sus* sp.	137	-12.4	13.19	-25.6	-6
NL-216	Laos	Nam Lot	*Sus* sp.	137	-12.8	13.19	-26.0	-5.3
NL-218	Laos	Nam Lot	*Sus* sp.	137	-14.1	13.19	-27.3	-6.8
NL-SS-1	Laos	Nam Lot	*Sus* sp.	137	-13.1	13.19	-26.3	-6
NL-258	Laos	Nam Lot	*Tapirus* sp.	300	-17.5	13.5	-31.0	-6.4
NL-259	Laos	Nam Lot	*Tapirus* sp.	300	-14.9	13.5	-28.4	-6.8
NL-260	Laos	Nam Lot	*Tapirus* sp.	300	-15.3	13.5	-28.8	-5
NL-286	Laos	Nam Lot	*Cuon alpinus*	15	-13.3	13.39	-26.7	-6.4
NL-368	Laos	Nam Lot	*Cuon alpinus*	15	-13	13.39	-26.4	-3

NL-269	Laos	Nam Lot	*Ursus thibetanus* cf. *kokeni*	100	-15.3	13.3	-28.6	-9
NL-271	Laos	Nam Lot	*Ursus thibetanus* cf. *kokeni*	100	-14.1	13.3	-27.4	-8.3
NL-275	Laos	Nam Lot	*Ursus thibetanus* cf. *kokeni*	100	-12.6	13.3	-25.9	-6.5
NL-310	Laos	Nam Lot	*Ursus thibetanus* cf. *kokeni*	100	-13.4	13.3	-26.7	-3.1
NL-288	Laos	Nam Lot	*Crocuta crocuta*	70	-13.7	14.04	-27.7	-6.4
NL-295	Laos	Nam Lot	*Crocuta crocuta*	70	-13.6	14.04	-27.6	-6.1
NL-433*	Laos	Nam Lot	*Pongo* sp.	55	-14.5	12.78	-27.3	-3.3
NL-302	Laos	Nam Lot	*Pongo* sp.	55	-14.5	12.78	-27.3	-6.1
NL-297	Laos	Nam Lot	*Macaca* sp.	6	-14.1	11.91	-26.0	-4.8
NL-314	Laos	Nam Lot	*Macaca* sp.	6	-11.5	11.91	-23.4	-3.7
NL-323	Laos	Nam Lot	*Macaca* sp.	6	-14.8	11.91	-26.7	-6.1
NL-357	Laos	Nam Lot	*Macaca* sp.	6	-14.6	11.91	-26.5	-3.6
NL-362	Laos	Nam Lot	*Elephas* sp.	4250	-16.1	14.69	-30.8	-6.2
NL-365	Laos	Nam Lot	*Stegodon orientalis*	4000	-18.2	14.66	-32.9	-4.3
NL-367	Laos	Nam Lot	*Stegodon orientalis*	4000	-15.4	14.66	-30.1	-6.5
NL-369	Laos	Nam Lot	*Hystrix* sp.	12	-13.6	12.18	-25.8	-6.5
NL-385	Laos	Nam Lot	*Hystrix* sp.	12	-12.1	12.18	-24.3	-5.7
NL-392	Laos	Nam Lot	*Hystrix* sp.	12	-14.1	12.18	-26.3	-5.2
NL-397	Laos	Nam Lot	*Hystrix* sp.	12	-13.3	12.18	-25.5	-5.7
NLII-1	Laos	Nam Lot	*Crocuta crocuta*	70	-14.3	14.04	-28.3	-6.6
NLII-2	Laos	Nam Lot	*Crocuta crocuta*	70	-12.5	14.04	-26.5	-6.8
NLII-3	Laos	Nam Lot	*Crocuta crocuta*	70	-15.1	14.04	-29.1	-6.2
NLII-4	Laos	Nam Lot	*Crocuta crocuta*	70	-11.8	14.04	-25.8	-7.2
NLII-5	Laos	Nam Lot	Felidae (*Neofelis nebulosa* ?)	18	-15	13.46	-28.5	-6.6
MI-20	Laos	Tam Hay Marklot	*Capricornis* cf. *sumatraensis*	112	-13.4	13.14	-26.5	-2.2
MI-21	Laos	Tam Hay Marklot	*Capricornis* cf. *sumatraensis*	112	-14.7	13.14	-27.8	-5.2
MI-22	Laos	Tam Hay Marklot	*Capricornis* cf. *sumatraensis*	112	-15.2	13.14	-28.3	-5.2
MI-23	Laos	Tam Hay Marklot	*Capricornis* cf. *sumatraensis*	112	-13.5	13.14	-26.6	-7
MI-24	Laos	Tam Hay Marklot	*Capricornis* cf. *sumatraensis*	112	-14.6	13.14	-27.7	-7.6
MI-25	Laos	Tam Hay Marklot	*Naemorhedus* cf. *caudatus*	27	-2.4	12.24	-14.6	0.2
MI-26	Laos	Tam Hay Marklot	*Naemorhedus* cf. *caudatus*	27	-3.9	12.24	-16.1	-2.5
MI-27	Laos	Tam Hay Marklot	*Naemorhedus* cf. *caudatus*	27	-3.7	12.24	-15.9	-1.8
MI-28	Laos	Tam Hay Marklot	*Naemorhedus* cf. *caudatus*	27	-2.5	12.24	-14.7	-1.6
MI-103	Laos	Tam Hay Marklot	*Helarctos malayanus*	50	-14.7	12.59	-27.3	-3.9
MI-121	Laos	Tam Hay Marklot	*Helarctos malayanus*	50	-14.9	12.59	-27.5	-5.5
MI-117	Laos	Tam Hay Marklot	*Ursus thibetanus*	100	-15.4	13.3	-28.7	-7.4
MI-119	Laos	Tam Hay Marklot	*Ursus thibetanus*	100	-13.3	13.3	-26.6	-6.6
MI-122	Laos	Tam Hay Marklot	*Ursus thibetanus*	100	-14.4	13.3	-27.7	-6.3
MI-134	Laos	Tam Hay Marklot	*Panthera pardus*	41	-7.9	13.81	-21.7	-7.3
MI-135	Laos	Tam Hay Marklot	*Panthera pardus*	41	-4	13.81	-17.8	-7.3
MI-136	Laos	Tam Hay Marklot	*Panthera pardus*	41	-13.8	13.81	-27.6	-6.8

MI-166	Laos	Tam Hay Marklot	*Rusa unicolor*	220	-7.5	13.59	-21.1	-5.7
MI-180	Laos	Tam Hay Marklot	*Rusa unicolor*	220	-5.3	13.59	-18.9	-4.4
MI-185	Laos	Tam Hay Marklot	*Rusa unicolor*	220	-6	13.59	-19.6	-4.9
MI-187	Laos	Tam Hay Marklot	*Rusa unicolor*	220	-3.4	13.59	-17.0	-4.9
MI-191	Laos	Tam Hay Marklot	*Rusa unicolor*	220	-7.9	13.59	-21.5	-6.1
MI-512	Laos	Tam Hay Marklot	*Rucervus eldii*	123	2.4	13.19	-10.8	-5.3
MI-595	Laos	Tam Hay Marklot	*Rucervus eldii*	123	1.8	13.19	-11.4	-3.1
MI-556	Laos	Tam Hay Marklot	*Axis* cf. *porcinus*	43	-0.8	12.53	-13.3	-5.8
MI-557	Laos	Tam Hay Marklot	*Axis* cf. *porcinus*	43	-0.6	12.53	-13.1	-5.3
MI-627	Laos	Tam Hay Marklot	*Muntiacus* sp.	24	-13.8	12.17	-26.0	-7.4
MI-628	Laos	Tam Hay Marklot	*Muntiacus* sp.	24	-14.8	12.17	-27.0	-8.1
MI-629	Laos	Tam Hay Marklot	*Muntiacus* sp.	24	-12.7	12.17	-24.9	-8.1
MI-630	Laos	Tam Hay Marklot	*Muntiacus* sp.	24	-14.4	12.17	-26.6	-5
MI-631	Laos	Tam Hay Marklot	*Muntiacus* sp.	24	-14.5	12.17	-26.7	-7.5
MI-650	Laos	Tam Hay Marklot	*Bubalus bubalis*	1000	-10.3	14.66	-25.0	-6
MI-651	Laos	Tam Hay Marklot	*Bubalus bubalis*	1000	-4	14.66	-18.7	-6.6
MI-652	Laos	Tam Hay Marklot	*Bubalus bubalis*	1000	1	14.66	-13.7	-4.7
MI-653	Laos	Tam Hay Marklot	*Bubalus bubalis*	1000	-10.9	14.66	-25.6	-6.3
MI-654	Laos	Tam Hay Marklot	*Bubalus bubalis*	1000	0.1	14.66	-14.6	-5.9
MI-655	Laos	Tam Hay Marklot	*Bos* sp.	800	-2.5	14.5	-17.0	-4.2
MI-656	Laos	Tam Hay Marklot	*Bos* sp.	800	-14.4	14.5	-28.9	-6.1
MI-657	Laos	Tam Hay Marklot	*Bos* sp.	800	-0.3	14.5	-14.8	-4.6
MI-658	Laos	Tam Hay Marklot	*Bos* sp.	800	-10	14.5	-24.5	-7.6
MI-659	Laos	Tam Hay Marklot	*Bos* sp.	800	-13.1	14.5	-27.6	-5.9
MI-130	Laos	Tam Hay Marklot	*Panthera tigris*	212	-4.3	14.53	-18.8	-3.2
MI-693	Laos	Tam Hay Marklot	*Panthera tigris*	212	-6.9	14.53	-21.4	-3.5
MI-694	Laos	Tam Hay Marklot	*Panthera tigris*	212	-10	14.53	-24.5	-6.8
MI-662	Laos	Tam Hay Marklot	*Sus* sp.	137	-7.6	13.19	-20.8	-7.5
MI-663	Laos	Tam Hay Marklot	*Sus* sp.	137	-8.6	13.19	-21.8	-10
MI-664	Laos	Tam Hay Marklot	*Sus* sp.	137	-6	13.19	-19.2	-5.9
MI-665	Laos	Tam Hay Marklot	*Sus* sp.	137	-13.5	13.19	-26.7	-5.8
MI-666	Laos	Tam Hay Marklot	*Sus* sp.	137	-14	13.19	-27.2	-7.6
MI-667	Laos	Tam Hay Marklot	*Sus* sp.	137	-13.2	13.19	-26.4	-5.4
MI-131	Laos	Tam Hay Marklot	*Cuon alpinus*	15	-16	13.39	-29.4	-7.3
MI-681	Laos	Tam Hay Marklot	*Cuon alpinus*	15	-11.4	13.39	-24.8	-3.2
MI-683	Laos	Tam Hay Marklot	*Pongo* sp.	55	-14.8	12.78	-27.6	-4.1
MI-685	Laos	Tam Hay Marklot	*Pongo* sp.	55	-13.5	12.78	-26.3	-4.6
MI-682	Laos	Tam Hay Marklot	Canidae	15	-13.2	13.39	-26.6	-6
MI-684	Laos	Tam Hay Marklot	*Ailuropoda melanoleuca*	92	-16.7	10.51	-27.2	-6.3
MI-691	Laos	Tam Hay Marklot	Tapiridae	300	-15.5	13.5	-29.0	-5.9
MI-692	Laos	Tam Hay Marklot	*Tapirus* sp.	300	-11.3	13.5	-24.8	-7.9

MI-695	Laos	Tam Hay Marklot	*Macaca* sp.	6	-13.9	11.91	-25.8	-5.3
MI-696	Laos	Tam Hay Marklot	*Macaca* sp.	6	-14.2	11.91	-26.1	-5.3
MI-697	Laos	Tam Hay Marklot	*Macaca* sp.	6	-15.1	11.91	-27.0	-5.6
MI-698	Laos	Tam Hay Marklot	*Macaca* sp.	6	-13.3	11.91	-25.2	-4.7
MI-699	Laos	Tam Hay Marklot	*Macaca* sp.	6	-12.9	11.91	-24.8	-4.6
MI-700	Laos	Tam Hay Marklot	*Hystrix* sp.	12	-11.5	12.18	-23.7	-7.6
MI-701	Laos	Tam Hay Marklot	*Hystrix* sp.	12	-11.5	12.18	-23.7	-5.5
MI-702	Laos	Tam Hay Marklot	*Hystrix* sp.	12	-7.9	12.18	-20.1	-5.2
MI-703	Laos	Tam Hay Marklot	*Hystrix* sp.	12	-11.6	12.18	-23.8	-8.5
MI-686	Laos	Tam Hay Marklot	*Rhinoceros sondaicus*	1750	-16.2	14.28	-30.5	-6.5
MI-687	Laos	Tam Hay Marklot	*Rhinoceros sondaicus*	1750	-13.3	14.28	-27.6	-5.5
MI-688	Laos	Tam Hay Marklot	*Rhinoceros sondaicus*	1750	-15.6	14.28	-29.9	-6.3
MI-689	Laos	Tam Hay Marklot	*Rhinoceros sondaicus*	1750	-15.3	14.28	-29.6	-7.1
MI-690	Laos	Tam Hay Marklot	*Rhinoceros sondaicus*	1750	-14.6	14.28	-28.9	-7.5

Annex S3. Data from Pleistocene Chinese sites ($\delta^{13}C_{apatite}$, $\delta^{13}C_{carbon\ source}$ and $\delta^{18}O_{apatite}$values (‰ VPDB), body mass and $\delta^{13}C$ (‰ VPDB) Enrichment Factor used to obtain $\delta^{13}C_{carbon\ source}$).

Yugong cave, Guangxi Zhuang Autonomous region, southern China (~22° 14” N, 107° 23” E). Dated to the late Middle Pleistocene on the composition of the faunal assemblage, in comparison with those of other sites from south China (Sanhe, Boyueshan, Juyuan, Mohui) (Sun et al., 2019). Faunal list (non- exhaustive list): *Sus xiaozhu wenzhongi*, *Sus* cf. *pei*, *Muntiacus* sp., *Cervus* (*Rusa*) cf. *unicolor*, Caprinae gen. et sp. indet., *Leptobos* sp., *Bos* (*Bibos*) sp., *Ailuropoda baconi*, *Ursus thibetanus*, *Stegodon* sp.

Baxian cave, Guangxi Zhuang Autonomous region, southern China (22° 34” N, 107° 21” E). Dated to the early Late Pleistocene on the similarity of the faunal assemblage with those from Zhiren, Fuyan, Daoxian, and Hunan sites (Ma et al., 2017; Sun et al., 2019). Faunal list: *Macaca* sp., *Namascus* sp., *Rhinopithecus* sp., *Pongo* sp., *Ailuropoda baconi*, *Ursus thibetanus*, *Arctonyx collaris*, *Panthera tigris*, *Elephas maximus*, *Stegodon orientalis*, *Rhinoceros sondaicus*, *Megatapirus augustus*, *Sus scrofa*, *Muntiacus* sp., *Cervus* (*Rusa*) sp., *Bos* (*Bibos*) sp.

Quzai cave, Guangxi Zhuang Autonomous region, southern China (22° 27” N, 107° 46” E). Dated to the early Late Pleistocene on the similarity of the faunal assemblage with those from Zhiren and Fuyan (Ma et al., 2019). Faunal list: *Macaca* sp., *Namascus* sp., *Rhinopithecus* sp., *Pongo* sp., *Ailuropoda baconi*, *Ursus thibetanus*, *Arctonyx collaris*, *Felis* sp., *Rhinoceros sondaicus*, *Megatapirus augustus*, *Elephas maximus*, *Stegodon orientalis*, *Sus scrofa*, *Cervus unicolor*, *Bos* (*Bibos*) sp.

**Country**	**Site**	**Taxon**	**Body Mass (kg)**	**$\delta^{13}C$ (‰) Enrichment Factor**	**$\delta^{13}C_{carbon\ source}$ (‰ VPDB)**	**$\delta^{13}C_{apatite}$ (‰ VPDB)**	**$\delta^{18}O_{apatite}$ (‰ VPDB)**
China	Yugong	*Cervus* sp.	220	13,59	-27.59	-14	-6.7
China	Yugong	*Cervus* sp.	220	13.59	-25.49	-11.9	-7.9
China	Yugong	*Cervus* sp.	220	13.59	-27.49	-13.9	-5.9
China	Yugong	*Cervus* sp.	220	13.59	-33.79	-20.2	-6.2
China	Yugong	*Leptobos* sp.	320	13.85	-33.05	-19.2	-5.7
China	Yugong	*Leptobos* sp.	320	13.85	-30.85	-17	-6.5
China	Yugong	*Leptobos* sp.	320	13.85	-27.65	-13.8	-5.9
China	Yugong	*Leptobos* sp.	320	13.85	-20.05	-6.2	-6.5
China	Yugong	*Rhinoceros sondaicus*	1750	14.28	-30.48	-16.2	-6.6
China	Yugong	*Rhinoceros sondaicus*	1750	14.28	-30.48	-16.2	-7
China	Yugong	*Rhinoceros sondaicus*	1750	14.28	-29.28	-15	-8.2

China	Yugong	*Rhinoceros sondaicus*	1750	14.28	-29.08	-14.8	-6.4
China	Yugong	*Stegodon* sp.	4000	14.66	-30.16	-15.5	-7.1
China	Yugong	*Stegodon* sp.	4000	14.66	-30.36	-15.7	-8.3
China	Yugong	*Stegodon* sp.	4000	14.66	-31.26	-16.6	-8.2
China	Yugong	*Ailuropoda baconi*	92	10.51	-26.51	-16	-6.2
China	Yugong	*Ailuropoda baconi*	92	10.51	-26.91	-16.4	-5.7
China	Yugong	*Ailuropoda baconi*	92	10.51	-28.01	-17.5	-4.1
China	Yugong	*Ailuropoda baconi*	92	10.51	-27.61	-17.1	-6.2
China	Yugong	*Ailuropoda baconi*	92	10.51	-28.81	-18.3	-6.3
China	Yugong	*Ailuropoda baconi*	92	10.51	-27.91	-17.4	-5.3
China	Yugong	*Ailuropoda baconi*	100	13.3	-26.9	-13.6	-5.7
China	Yugong	*Ailuropoda baconi*	100	13.3	-26.7	-13.4	-5.6
China	Yugong	Suidae	137	13.19	-28.79	-15.6	-6.9
China	Yugong	Suidae	137	13.19	-27.49	-14.3	-6.4
China	Yugong	Suidae	137	13.19	-27.79	-14.6	-6.5
China	Yugong	Suidae	137	13.19	-25.59	-12.4	-7.7
China	Yugong	Suidae	137	13.19	-26.59	-13.4	-7.7
China	Yugong	Suidae	137	13,19	-27,99	-14,8	-8,7
China	Baxian	*Elephas maximus*	4250	14,69	-29,19	-14,5	-9
China	Baxian	*Elephas maximus*	4250	14,69	-31,29	-16,6	-6,8
China	Baxian	*Elephas maximus*	4250	14,69	-30,79	-16,1	-6,3
China	Baxian	*Elephas maximus*	4250	14,69	-31,49	-16,8	-6,6
China	Baxian	*Elephas maximus*	4250	14,69	-30,09	-15,4	-8,4
China	Baxian	*Elephas maximus*	4250	14,69	-32,29	-17,6	-6,8
China	Baxian	*Elephas maximus*	4250	14,69	-30,49	-15,8	-5,9
China	Baxian	*Elephas maximus*	4250	14,69	-28,99	-14,3	-8,5
China	Baxian	*Elephas maximus*	4250	14,69	-31,89	-17,2	-7,2
China	Baxian	*Elephas maximus*	4250	14.69	-29.79	-15.1	-6.4
China	Baxian	*Elephas maximus*	4250	14.69	-29.19	-14.5	-8.2
China	Baxian	*Cervus* sp.	220	13.59	-32.89	-19.3	-7.3
China	Baxian	*Cervus* sp.	220	13.59	-30.59	-17	-7.1
China	Baxian	*Muntiacus* sp.	24	12.17	-26.57	-14.4	-7.1
China	Baxian	*Muntiacus* sp.	24	12.17	-27.77	-15.6	-4.6
China	Baxian	*Muntiacus* sp.	24	12.17	-27.67	-15.5	-5.8
China	Baxian	*Sus scrofa*	137	13.19	-28.89	-15.7	-7.8

China	Baxian	*Sus scrofa*	137	13.19	-27.79	-14.6	-6.9
China	Baxian	*Sus scrofa*	137	13.19	-26.09	-12.9	-6.7
China	Baxian	*Rhinoceros sondaicus*	1750	14.28	-31.38	-17.1	-7.3
China	Baxian	*Rhinoceros sondaicus*	1750	14.28	-30.58	-16.3	-5.5
China	Baxian	*Rhinoceros sondaicus*	1750	14.28	-31.48	-17.2	-6.8
China	Baxian	*Rhinoceros sondaicus*	1750	14.28	-32.38	-18.1	-9.2
China	Baxian	*Rhinoceros sondaicus*	1750	14.28	-30.58	-16.3	-8.5
China	Baxian	*Megatapirus augustus*	500	13.72	-31.22	-17.5	-6.1
China	Baxian	*Stegodon orientalis*	4000	14.66	-30.56	-15.9	-8.4
China	Baxian	*Stegodon orientalis*	4000	14.66	-30.16	-15.5	-8.2
China	Baxian	*Bos* (*Bibos*) sp.	800	14.5	-29.2	-14.7	-6.6
China	Baxian	*Macaca* sp.	6	11.91	-27.81	-15.9	-7.1
China	Baxian	*Ailuropoda baconi*	92	10.51	-26.41	-15.9	-8
China	Baxian	*Ailuropoda baconi*	92	10.51	-28.51	-18	-7.1
China	Baxian	*Ailuropoda baconi*	92	10.51	-27.81	-17.3	-6.4
China	Baxian	*Cervus* sp.	220	13.59	-33.59	-20	-4.7
China	Baxian	*Cervus* sp.	220	13.59	-26.69	-13.1	-5.9
China	Baxian	Suidae	137	13.19	-27.99	-14.8	-9.1
China	Baxian	Suidae	137	13.19	-28.29	-15.1	-6.9
China	Baxian	Suidae	137	13.19	-27.89	-14.7	-7.1
China	Baxian	*Ursus thibetanus*	100	13.3	-30	-16.7	-6.9
China	Baxian	*Ursus thibetanus*	100	13.3	-29.8	-16.5	-8.1
China	Baxian	*Ursus thibetanus*	100	13.3	-28.4	-15.1	-9.8
China	Baxian	*Ursus thibetanus*	100	13.3	-28.7	-15.4	-7.5
China	Baxian	*Ursus thibetanus*	100	13.3	-28.8	-15.5	-7.2
China	Baxian	*Ailuropoda melanoleuca*	92	10.51	-25.41	-14.9	-3
China	Baxian	*Ailuropoda melanoleuca*	92	10.51	-28.71	-18.2	-2
China	Baxian	*Ailuropoda melanoleuca*	92	10.51	-28.11	-17.6	-4.2
China	Baxian	*Ailuropoda melanoleuca*	92	10.51	-28.11	-17.6	-2.7
China	Baxian	*Ailuropoda melanoleuca*	92	10.51	-28.11	-17.6	-4.6
China	Baxian	*Ailuropoda melanoleuca*	92	10.51	-27.81	-17.3	-5.3
China	Baxian	*Ailuropoda melanoleuca*	92	10.51	-28.81	-18.3	-4.7
China	Baxian	*Ailuropoda melanoleuca*	92	10.51	-27.81	-17.3	-4.7
China	Baxian	*Ailuropoda melanoleuca*	92	10.51	-27.81	-17.3	-5.8
China	Baxian	*Ailuropoda melanoleuca*	92	10.51	-26.91	-16.4	-6.5

China	Ouzai	*Elephas maximus*	4250	14.69	-27.79	-13.1	-4.7
China	Ouzai	*Elephas maximus*	4250	14.69	-30.19	-15.5	-5
China	Ouzai	*Elephas maximus*	4250	14.69	-31.49	-16.8	-5.6
China	Ouzai	*Elephas maximus*	4250	14.69	-32.59	-17.9	-7.5
China	Ouzai	*Elephas maximus*	4250	14.69	-30.59	-15.9	-6.2
China	Ouzai	*Elephas maximus*	4250	14.69	-26.59	-11.9	-6
China	Ouzai	*Elephas maximus*	4250	14.69	-28.69	-14	-5.3
China	Ouzai	*Elephas maximus*	4250	14.69	-31.19	-16.5	-5.7
China	Ouzai	*Elephas maximus*	4250	14.69	-28.59	-13.9	-4.6
China	Ouzai	*Elephas maximus*	4250	14.69	-28.79	-14.1	-5.1
China	Ouzai	*Stegodon orientalis*	4000	14.66	-31.26	-16.6	-7.1
China	Ouzai	*Stegodon orientalis*	4000	14.66	-29.36	-14.7	-4.1
China	Ouzai	*Stegodon orientalis*	4000	14.66	-29.96	-15.3	-7.2
China	Ouzai	*Stegodon orientalis*	4000	14.66	-31.06	-16.4	-7.9
China	Ouzai	*Stegodon orientalis*	4000	14.66	-30.96	-16.3	-7.2
China	Ouzai	*Stegodon orientalis*	4000	14.66	-31.36	-16.7	-5
China	Ouzai	*Stegodon orientalis*	4000	14.66	-31.06	-16.4	-5.7
China	Ouzai	*Bos* (*Bibos*) sp.	800	14.5	-34.4	-19.9	-5.4
China	Ouzai	*Bos* (*Bibos*) sp.	800	14.5	-31.1	-16.6	-5.9
China	Ouzai	*Rusa unicolor*	220	13.59	-29.89	-16.3	-4.3
China	Ouzai	*Rusa unicolor*	220	13.59	-25.89	-12.3	-5.7
China	Ouzai	*Rusa unicolor*	220	13.59	-31.09	-17.5	-3.2
China	Ouzai	*Rhinoceros sondaicus*	1750	14.28	-26.28	-12	-5.5
China	Ouzai	*Rhinoceros sondaicus*	1750	14.28	-29.98	-15.7	-6.5
China	Ouzai	*Rhinoceros sondaicus*	1750	14.28	-31.68	-17.4	-5.5
China	Ouzai	*Rhinoceros sondaicus*	1750	14.28	-30.18	-15.9	-6.6
China	Ouzai	*Megatapirus augustus*	500	13.72	-29.02	-15.3	-6.4
China	Ouzai	*Megatapirus augustus*	500	13.72	-29.42	-15.7	-7.1
China	Ouzai	*Ursus thibetanus*	100	13.3	-27	-13.7	-8.2
China	Ouzai	*Ursus thibetanus*	100	13.3	-25.3	-12	-7.8
China	Ouzai	*Ursus thibetanus*	100	13.3	-25.6	-12.3	-7.9
China	Ouzai	*Sus scrofa*	137	13.19	-26.49	-13.3	-6.1
China	Ouzai	*Sus scrofa*	137	13.19	-27.19	-14	-7.9
China	Ouzai	*Sus scrofa*	137	13.19	-24.29	-11.1	-7.2
China	Ouzai	*Ailuropoda baconi*	92	10.51	-25.71	-15.2	-6.3

China	Ouzai	*Cuon* sp.	15	13.39	-25.29	-11.9	-7.3

Annex S4. Data from Pleistocene sites of Thailand ($\delta^{13}C_{apatite}$, $\delta^{13}C_{carbon\ source}$ and $\delta^{18}O_{apatite}$values (‰ VPDB), body mass and $\delta^{13}C$ (‰ VPDB) Enrichment Factor used to obtain $\delta^{13}C_{carbon\ source}$).

Tham Lod rockshelter, Pang Mapha District, Mae Hong Son, Northwest Thailand. The estimated age interval of Tham Lod (radiocarbon ^{14}C dates on organic materials and on freshwater bivalves *Margaritanopsis laosensis*, and thermoluminescence on sediments gave a 33,000 – 11,500 BP age interval; Marwick and Gagan, 2011). Isotopic records published in Suraprasit et al. (2020).

Faunal list: *Macaca arctoides*, *M. mulatta*, *Ursus thibetanus*, *Arctonyx collaris*, *Panthera tigris*, *Elephas maximus*, *Rhinoceros* sp., *Sus scrofa*, *Muntiacus muntjac*, *Rusa unicolor*, *Rucervus eldii*, *Axis porcinus*, *Bos* sp., *Bos javanicus*, *Bos gaurus*, *Bubalus arnee*, *Naemorhedus goral, N. griseus, Capricornis sumatraensis* (Wattanapituksakul et al., 2018).

Tham Wiman Nakin, Khon San Disctrict, Chaiyaphum, northeast Thailand, dated to >~170 ka (U-series dating on calcite) (Esposito et al., 1998, 2002). Isotopic records published in Pushkina et al., (2010) and Suraprasit et al. (2020).

Faunal list: *Macaca* cf. *nemestrina*, *Trachypithecus* sp., *Pongo pygmaeus, Crocuta Crocuta ultima, Ailuropoda melanoleuca baconi, Ursus thibetanus, Martes flavigula*, *Arctonyx collaris rostratus, Paguma larvata, Paradoxurus hermaphroditus, Elephas* cf. *maximus*, *Rhinoceros* cf. *unicornis*, *Rhinoceros sondaicus, Tapirus indicus* cf. *intermedius*, *Sus scrofa, Sus* cf. *barbatus*, *Bos javanicus, Bos sauveli, Bos frontalis, Bubalus bubalis, Naemorhedus sumatraensis* cf. *kanjereus*, *Muntiacus muntjac, Cervus unicolor, Cervus eldii, Axis porcinus*, Cervinae indet. (Tougard et al., 1998), *Meles* cf. *leucurus*, *Panthera* cf. *tigris*, *Naemorhedus goral* (Suraprasit et al., in press).

**Country**	**Site**	**Taxon**	**Body Mass (kg)**	**$\delta^{13}C$ (‰) Enrichment Factor**	**$\delta^{13}C_{carbon\ source}$ (‰ VPDB)**	**$\delta^{13}C_{apatite}$ (‰ VPDB)**	**$\delta^{18}O_{apatite}$ (‰ VPDB)**
Thailand	Tham Lod	*Naemorhedus goral*	28	12,26	-13.26	-1	-3.7
Thailand	Tham Lod	*Naemorhedus goral*	28	12.26	-16.26	-4	-1.8
Thailand	Tham Lod	*Naemorhedus goral*	28	12.26	-12.86	-0.6	-7.3
Thailand	Tham Lod	*Naemorhedus goral*	28	12.26	-14.76	-2.5	-5.5
Thailand	Tham Lod	*Naemorhedus goral*	28	12.26	-12.36	-0.1	-11.4
Thailand	Tham Lod	*Naemorhedus goral*	28	12.26	-17.56	-5.3	-7.5
Thailand	Tham Lod	*Naemorhedus goral*	28	12.26	-16.86	-4.6	-2.8
Thailand	Tham Lod	*Naemorhedus goral*	28	12.26	-16.76	-4.5	-7.4
Thailand	Tham Lod	*Naemorhedus griseus*	27	12.24	-12.64	-0.4	-2.8

Thailand	Tham Lod	*Naemorhedus griseus*	27	12.24	-16.54	-4.3	-5
Thailand	Tham Lod	*Naemorhedus griseus*	27	12.24	-13.04	-0.8	-6.9
Thailand	Tham Lod	*Naemorhedus griseus*	27	12.24	-12.04	0.2	-5.6
Thailand	Tham Lod	*Naemorhedus griseus*	27	12.24	-11.64	0.6	-5.4
Thailand	Tham Lod	*Naemorhedus griseus*	27	12.24	-11.84	0.4	-8.9
Thailand	Tham Lod	*Naemorhedus griseus*	27	12.24	-15.44	-3.2	-4.6
Thailand	Tham Lod	*Naemorhedus griseus*	27	12.24	-19.34	-7.1	-0.3
Thailand	Tham Lod	*Naemorhedus griseus*	27	12.24	-12.04	0.2	-6.1
Thailand	Tham Lod	*Naemorhedus griseus*	27	12.24	-15.24	-3	-3.5
Thailand	Tham Lod	*Naemorhedus griseus*	27	12.24	-11.34	0.9	-6.7
Thailand	Tham Lod	*Naemorhedus griseus*	27	12.24	-10.34	1.9	-3.90
Thailand	Tham Lod	*Naemorhedus griseus*	27	12.24	-12.54	-0.3	-7.60
Thailand	Tham Lod	*Naemorhedus griseus*	27	12.24	-15.24	-3	-6.50
Thailand	Tham Lod	*Naemorhedus griseus*	27	12.24	-15.14	-2.9	-7.80
Thailand	Tham Lod	*Naemorhedus griseus*	27	12.24	-12.24	0	-4.1
Thailand	Tham Lod	*Naemorhedus griseus*	27	12.24	-11.74	0.5	-4.4
Thailand	Tham Lod	*Naemorhedus griseus*	27	12.24	-12.84	-0.6	-6.7
Thailand	Tham Lod	*Naemorhedus griseus*	27	12.24	-15.94	-3.7	-7.2
Thailand	Tham Lod	*Capricornis sumatraensis*	112	13.14	-15.84	-2.7	0.1
Thailand	Tham Lod	*Capricornis sumatraensis*	112	13.14	-25.44	-12.3	-7.9
Thailand	Tham Lod	*Capricornis sumatraensis*	112	13.14	-25.94	-12.8	-5.6
Thailand	Tham Lod	*Capricornis sumatraensis*	112	13.14	-24.64	-11.5	-6.9
Thailand	Tham Lod	*Capricornis sumatraensis*	112	13.14	-25.44	-12.3	-8.1
Thailand	Tham Lod	*Capricornis sumatraensis*	112	13.14	-26.34	-13.2	-7.2
Thailand	Tham Lod	*Capricornis sumatraensis*	112	13.14	-27.14	-14	-3.9
Thailand	Tham Lod	*Capricornis sumatraensis*	112	13.14	-16.74	-3.6	-4.4
Thailand	Tham Lod	*Capricornis sumatraensis*	112	13.14	-25.14	-12	-6.3
Thailand	Tham Lod	*Capricornis sumatraensis*	112	13.14	-17.74	-4.6	-6.4
Thailand	Tham Lod	*Capricornis sumatraensis*	112	13.14	-11.24	1.9	-7.8
Thailand	Tham Lod	*Capricornis sumatraensis*	112	13.14	-27.44	-14.3	-4.1
Thailand	Tham Lod	*Ursus thibetanus*	100	13.3	-28.60	-15.3	-5.8
Thailand	Tham Lod	*Ursus thibetanus*	100	13.3	-27.10	-13.8	-5.5
Thailand	Tham Lod	*Ursus thibetanus*	100	13.3	-26.40	-13.1	-6.2
Thailand	Tham Lod	*Panthera tigris*	212	14.53	-27.53	-13	-7.4
Thailand	Tham Lod	*Sus scrofa*	137	13.19	-21.09	-7.9	-9.7

Thailand	Tham Lod	*Sus scrofa*	137	13.19	-20.09	-6.9	-10.1
Thailand	Tham Lod	*Rucervus eldii*	123	13.19	-10.89	2.3	-5.5
Thailand	Tham Lod	*Rucervus eldii*	123	13.19	-11.69	1.5	-6.1
Thailand	Tham Lod	*Rusa unicolor*	220	13.59	-18.79	-5.2	-8.6
Thailand	Tham Lod	*Rusa unicolor*	220	13.59	-18.49	-4.9	-8
Thailand	Tham Lod	*Bubalus arnee*	1000	14.66	-12.26	2.4	-6.4
Thailand	Tham Lod	*Bubalus arnee*	1000	14.66	-11.66	3	-7.5
Thailand	Tham Wiman Nakin	*Naemorhedus goral*	28	12.26	-15.76	-3.5	-2.7
Thailand	Tham Wiman Nakin	*Naemorhedus goral*	28	12.26	-13.86	-1.6	-6.5
Thailand	Tham Wiman Nakin	*Naemorhedus goral*	28	12.26	-11.86	0.4	-5.7
Thailand	Tham Wiman Nakin	*Naemorhedus goral*	28	12.26	-13.06	-0.8	-1.7
Thailand	Tham Wiman Nakin	*Capricornis sumatraensis*	112	13.14	-11.44	1.7	-3.6
Thailand	Tham Wiman Nakin	*Capricornis sumatraensis*	112	13.14	-14.44	-1.3	-4.9
Thailand	Tham Wiman Nakin	*Capricornis sumatraensis*	112	13.14	-24.84	-11.7	-5.5
Thailand	Tham Wiman Nakin	*Capricornis sumatraensis*	112	13.14	-25.94	-12.8	-8.9
Thailand	Tham Wiman Nakin	*Capricornis sumatraensis*	112	13.14	-14.74	-1.6	-6
Thailand	Tham Wiman Nakin	*Capricornis sumatraensis*	112	13.14	-23.34	-10.2	-5.7
Thailand	Tham Wiman Nakin	*Capricornis sumatraensis*	112	13.14	-24.14	-11	-5.2
Thailand	Tham Wiman Nakin	*Capricornis sumatraensis*	112	13.14	-24.54	-11.4	-7
Thailand	Tham Wiman Nakin	*Capricornis sumatraensis*	112	13.14	-25.14	-12	-6
Thailand	Tham Wiman Nakin	*Rhinoceros* sp.	2000	14.34	-29.54	-15.2	-2.9
Thailand	Tham Wiman Nakin	*Rhinoceros* sp.	2000	14.34	-29.04	-14.7	-7.3
Thailand	Tham Wiman Nakin	*Rhinoceros* sp.	2000	14.34	-29.44	-15.1	-3.7
Thailand	Tham Wiman Nakin	*Rhinoceros* sp.	2000	14.34	-29.24	-14.9	-7.8
Thailand	Tham Wiman Nakin	*Rhinoceros* sp.	2000	14.34	-28.74	-14.4	-8.5
Thailand	Tham Wiman Nakin	*Sus scrofa*	137	13.19	-26.19	-13	-6.6
Thailand	Tham Wiman Nakin	*Sus scrofa*	137	13.19	-21.79	-8.6	-4.5
Thailand	Tham Wiman Nakin	*Sus scrofa*	137	13.19	-21.09	-7.9	-7.4
Thailand	Tham Wiman Nakin	*Sus scrofa*	137	13.19	-26.29	-13.1	-9.7
Thailand	Tham Wiman Nakin	*Sus scrofa*	137	13.19	-26.99	-13.8	-8.8
Thailand	Tham Wiman Nakin	Cervidae	220	13.59	-15.79	-2.2	-8.7
Thailand	Tham Wiman Nakin	Cervidae	220	13.59	-12.99	0.6	-8
Thailand	Tham Wiman Nakin	Cervidae	220	13.59	-17.29	-3.7	-4
Thailand	Tham Wiman Nakin	Cervidae	220	13.59	-14.49	-0.9	-6.2
Thailand	Tham Wiman Nakin	Cervidae	220	13.59	-20.39	-6.8	-6.3

Thailand	Tham Wiman Nakin	*Crocuta crocuta*	70	14.04	-18.14	-4.1	-4.2
Thailand	Tham Wiman Nakin	*Crocuta crocuta*	70	14.04	-20.14	-6.1	-7
Thailand	Tham Wiman Nakin	*Hystrix* sp.	12	12.18	-21.58	-9.4	-5
Thailand	Tham Wiman Nakin	*Hystrix* sp.	12	12.18	-23.88	-11.7	-6.9
Thailand	Tham Wiman Nakin	*Hystrix* sp.	12	12.18	-20.48	-8.3	-7.9
Thailand	Tham Wiman Nakin	*Hystrix* sp.	12	12.18	-17.68	-5.5	-5.5
Thailand	Tham Wiman Nakin	*Hystrix* sp.	12	12.18	-25.08	-12.9	-7
Thailand	Tham Wiman Nakin	*Pongo* sp.	55	12.78	-25.88	-13.1	-4

Annex S5. Data from Boh Dambang, Cambodia (25 -18 ka; Bacon et al., 2018c) ($\delta^{13}C_{apatite}$, $\delta^{13}C_{carbon\ source}$ (‰ VPDB), body mass and $\delta^{13}C$ (‰ VPDB) Enrichment Factor used to obtain $\delta^{13}C_{carbon\ source}$).

**Number**	**Country**	**Site**	**Taxon**	**Body Mass (kg)**	**$\delta^{13}C_{apatite}$ (‰ VPDB)**	**$\delta^{13}C$ (‰) Enrichment Factor**	**$\delta^{13}C_{carbon\ source}$ (‰ VPDB)**
BD-477	Cambodia	Boh Dambang	*Bos* cf. *frontalis*	800	0.6	14.5	-15.1
BD-85	Cambodia	Boh Dambang		800	-2.7	14.5	-17.2
BD-95	Cambodia	Boh Dambang		800	-0.3	14.5	-14.8
BD-528	Cambodia	Boh Dambang		800	0.9	14.5	-15.4
BD-163	Cambodia	Boh Dambang	*Capricornis sumatraensis*	112	-11.9	13.14	-25.04
BD-363	Cambodia	Boh Dambang		112	-13.2	13.14	-26.34
BD-166	Cambodia	Boh Dambang		112	-11.0	13.14	-24.14
BD-443	Cambodia	Boh Dambang		112	-1.6	13.14	-14.74
BD-410	Cambodia	Boh Dambang	*Rucervus eldii*	123	1.6	13.19	-14.79
BD-134	Cambodia	Boh Dambang		123	-0.4	13.19	-13.59
BD-385	Cambodia	Boh Dambang		123	0.8	13.19	-13.99
BD-146	Cambodia	Boh Dambang		123	0.0	13.19	-13.19
BD-533	Cambodia	Boh Dambang	*Bubalus bubalis*	1000	-2.2	14.66	-16.86
BD-122	Cambodia	Boh Dambang		1000	-1.5	14.66	-16.16
BD-129	Cambodia	Boh Dambang		1000	2.8	14.66	-17.46
BD-388	Cambodia	Boh Dambang	*Rusa unicolor*	220	-14.6	13.59	-28.19
BD-179	Cambodia	Boh Dambang		220	-1.4	13.59	-14.99
BD-133	Cambodia	Boh Dambang		220	-0.7	13.59	-14.29
BD-138	Cambodia	Boh Dambang		220	-2.1	13.59	-15.69
BD-375	Cambodia	Boh Dambang		220	-12.3	13.59	-25.89
BD-381	Cambodia	Boh Dambang		220	-0.1	13.59	-13.69
BD-50	Cambodia	Boh Dambang	*Rhinoceros* sp.	1633	1.3	14.25	-15.55
BD-514	Cambodia	Boh Dambang		1633	-14.3	14.25	-28.55
BD-557	Cambodia	Boh Dambang		1633	-14.0	14.25	-28.25
BD-21b	Cambodia	Boh Dambang	*Sus* cf. *scrofa*	137	-6.3	13.19	-19.49
BD-20	Cambodia	Boh Dambang		137	-11.6	13.19	-24.79
BD-19	Cambodia	Boh Dambang		137	-7.0	13.19	-20.19
BD-184	Cambodia	Boh Dambang	*Muntiacus muntjak*	24	-13.8	12.17	-25.97

BD-438	Cambodia	Boh Dambang		24	-13.3	12.17	-25.47
BD-238	Cambodia	Boh Dambang	*Hystrix brachyura*	12	-13.5	12.18	-25.68
BD-240	Cambodia	Boh Dambang		12	-12.8	12.18	-24.98
BD-251	Cambodia	Boh Dambang		12	-14.1	12.18	-26.28
BD-41	Cambodia	Boh Dambang	*Pongo pygmaeus*	55	-14.3	12.78	-27.08
BD-45	Cambodia	Boh Dambang		55	-12.1	12.78	-24.88
BD-47	Cambodia	Boh Dambang		55	-13.2	12.78	-25.98
BD-331	Cambodia	Boh Dambang	*Macaca* sp.	6	-12.8	11.91	-24.71
BD-333	Cambodia	Boh Dambang		6	-12.5	11.91	-24.41
BD-257	Cambodia	Boh Dambang	*Crocuta crocuta ultima*	70	-3.5	14.04	-17.54
BD-204	Cambodia	Boh Dambang		70	-3.9	14.04	-17.94
BD-203	Cambodia	Boh Dambang		70	-4.1	14.04	-18.14
BD-252	Cambodia	Boh Dambang		70	-1.4	14.04	-15.44
BD-200	Cambodia	Boh Dambang		70	-4.1	14.04	-18.14
BD-263	Cambodia	Boh Dambang		70	-6.1	14.04	-20.14
BD-267	Cambodia	Boh Dambang		70	-4.3	14.04	-28.34
BD-285	Cambodia	Boh Dambang		70	-3.8	14.04	-17.84
BD-16	Cambodia	Boh Dambang	*Canis aureus*	8	-7.8	13.13	-20.98
BD-304	Cambodia	Boh Dambang		8	-5.4	13.13	-18.53
BD-278	Cambodia	Boh Dambang	*Panthera pardus*	41	-6.5	13.81	-20.31
BD-286	Cambodia	Boh Dambang		41	-14	13.81	-27.81
BD-291	Cambodia	Boh Dambang		41	-7.7	13.81	-21.51
BD-51	Cambodia	Boh Dambang	*Ursus thibetanus*	100	-2.6	13.3	-15.9
BD-52	Cambodia	Boh Dambang		100	-12.6	13.3	-25.9

Annex S6. Crown area dimensions of teeth (r, right; l, left; m, molar; p, premolar).

n°	Tooth type	Taxon	Length	Width	Crown area
**CM127**	rm3	*Muntiacus* sp.	18,7	9,58	179,15
**CM168**	rm3	*Muntiacus* sp.	18,27	9,79	178,86
**CM307**	lm3	*Muntiacus* sp.	17,05	9,03	153,96
**CM357**	lm3	*Muntiacus* sp.	18,61	9,99	185,91
**THS214**	lm3	*Muntiacus muntjak*	19,32	8,96	173,11
**THS217**	lm3	*Muntiacus muntjak*	18,15	8,81	159,90
**THS218**	lm3	*Muntiacus muntjak*	18,2	9,25	168,35
**THS893**	lm3	*Muntiacus muntjak*	20,77	9,15	190,05
**THS213**	rm3	*Muntiacus muntjak*	17,99	9,33	167,85
**THS216**	rm3	*Muntiacus muntjak*	18,34	8,54	156,62
**THS219**	rm3	*Muntiacus muntjak*	17,84	8,69	155,03
**THS895**	rm3	*Muntiacus muntjak*	18,59	9,15	170,10
**NL67b**	rm3	*Muntiacus muntjak*	16,5	7,7	127,05
**DU392**	lm3	*Muntiacus muntjak*	17,72	9,32	165,15
**DU426**	lm3	*Muntiacus muntjak*	14,13	7,3	103,15
**DU433**	lm3	*Muntiacus muntjak*	15,8	8,45	133,51
**DU437**	lm3	*Muntiacus muntjak*	16,34	8,78	143,47
**DU461**	lm3	*Muntiacus muntjak*	17,98	9,05	162,72
**DU465**	lm3	*Muntiacus muntjak*	18,88	9,18	173,32
**DU511**	lm3	*Muntiacus muntjak*	17,8	9,61	171,06
**DU394**	rm3	*Muntiacus muntjak*	16,94	8,75	148,23
**DU429**	rm3	*Muntiacus muntjak*	17,12	9,58	164,01
**DU436**	rm3	*Muntiacus muntjak*	17,43	9,07	158,09
**DU475**	rm3	*Muntiacus muntjak*	17,15	8,97	153,84
**DU1134**	rm3	*Muntiacus muntjak*	16,08	8,05	129,44
**THM627**	lm3	*Muntiacus* sp.	16,79	9,37	157,32
**THM628**	lm3	*Muntiacus* sp.	17,45	8,92	155,65
**THM629**	lm3	*Muntiacus* sp.	18,56	9,22	171,12
**THM630**	lm3	*Muntiacus* sp.	18,6	9,17	170,56
**THM631**	lm3	*Muntiacus* sp.	18,84	9,6	180,86
**THM632**	lm3	*Muntiacus* sp.	15,55	8,76	136,22
**THM633**	lm3	*Muntiacus* sp.	18,7	9,58	179,15
**THM634**	lm3	*Muntiacus* sp.	15,57	8,17	127,21
**THM635**	lm3	*Muntiacus* sp.	17,73	9,29	164,71
**THM636**	lm3	*Muntiacus* sp.	17,04	9,19	156,60
**THM637**	lm3	*Muntiacus* sp.	16,16	8,4	135,74
**THM638**	lm3	*Muntiacus* sp.	16,29	8,94	145,63
**THM639**	lm3	*Muntiacus* sp.	18,1	9,23	167,06
**THM640**	rm3	*Muntiacus* sp.	17,88	9,4	168,07
**THM642**	rm3	*Muntiacus* sp.	17,28	9,41	162,60
**THM643**	rm3	*Muntiacus* sp.	18,76	9,51	178,41
**THM644**	rm3	*Muntiacus* sp.	19	9,53	181,07

**CM157**	lm3	*Capricornis* sp.	26,11	11,26	294,00
**CM284**	rm3	*Capricornis* sp.	24,79	11,49	284,84
**THS570**	m3	*Capricornis sumatraensis*	24,3	11,3	274,59
**NL7**	lm3	*Capricornis sumatraensis*	25,7	9,9	254,43
**NL8**	rm3	*Capricornis sumatraensis*	24,9	10,6	263,94
**NL9**	rm3	*Capricornis sumatraensis*	24,8	10,3	255,44
**DU538**	lm3	*Capricornis sumatraensis*	26,84	11,41	306,24
**DU569**	lm3	*Capricornis sumatraensis*	26,24	11,69	306,75
**DU574**	rm3	*Capricornis sumatraensis*	26,33	11,49	302,53
**DU613**	rm3	*Capricornis sumatraensis*	25,47	10,56	268,96
**THM19**	lm3	*Capricornis* cf. *sumatraensis*	30,43	13,4	407,76
**THM20**	lm3	*Capricornis* cf. *sumatraensis*	25,28	10,81	273,28
**THM21**	lm3	*Capricornis* cf. *sumatraensis*	31,81	11,82	375,99
**THM22**	lm3	*Capricornis* cf. *sumatraensis*	24,94	12,66	315,74
**THM23**	lm3	*Capricornis* cf. *sumatraensis*	26,32	11,45	301,36
**THM24**	lm3	*Capricornis* cf. *sumatraensis*	29,02	10,88	315,74
**THM44**	rm3	*Capricornis* cf. *sumatraensis*	27,3	11,84	323,23
**THM45**	rm3	*Capricornis* cf. *sumatraensis*	24,27	11,36	275,71
**CM78**	rp3	*Rusa unicolor*	15,35	9,09	139,53
**CM93**	rp3	*Rusa unicolor*	17,6	9,63	169,49
**CM140**	rp3	*Rusa unicolor*	17,92	11,66	208,95
**CM183**	rp3	*Rusa unicolor*	14,32	8,4	120,29
**CM184**	rp3	*Rusa unicolor*	16,74	10	167,40
**CM217a**	rp3	*Rusa unicolor*	16,83	9,61	161,74
**CM270**	rp3	*Rusa unicolor*	13,18	8,65	114,01
**CM347**	rp3	*Rusa unicolor*	14,86	9,36	139,09
**CM379**	rp3	*Rusa unicolor*	13,35	8,05	107,47
**CM491**	rp3	*Rusa unicolor*	15,75	10,51	165,53
**CM862**	rp3	*Rusa unicolor*	16,96	10,91	185,03
**CM141**	lp3	*Rusa unicolor*	14,66	9,39	137,66
**CM142**	lp3	*Rusa unicolor*	16,64	8,45	140,61
**CM220**	lp3	*Rusa unicolor*	15,15	8,74	132,41
**CM269**	lp3	*Rusa unicolor*	16,69	9,74	162,56
**CM863**	lp3	*Rusa unicolor*	14,79	8,73	129,12
**THS495**	lp3	*Rusa unicolor*	17,95	9,82	176,27
**THS739**	lp3	*Rusa unicolor*	16,94	10,84	183,63
**THS744**	lp3	*Rusa unicolor*	13,42	8,97	120,38
**NL22**	rp3	*Rusa unicolor*	20,33	12,85	261,24
**NL23**	rp3	*Rusa unicolor*	21,7	12,85	278,85
**NL27**	rp3	*Rusa unicolor*	18,64	9,95	185,47
**NL29**	rp3	*Rusa unicolor*	20,08	11,27	226,30
**NL34**	rp3	*Rusa unicolor*	15,82	10,29	162,79
**NL25**	lp3	*Rusa unicolor*	22,88	11,08	253,51
**NL103**	lp3	*Rusa unicolor*	18,26	9,45	172,56
**NL104**	lp3	*Rusa unicolor*	23,31	13,22	308,16

**THM239**	rp3	*Rusa unicolor*	17,14	8,8	150,83
**THM241**	rp3	*Rusa unicolor*	16,34	9,49	155,07
**THM242**	rp3	*Rusa unicolor*	16,42	8,55	140,39
**THM243**	rp3	*Rusa unicolor*	17,7	9,83	173,99
**THM244**	rp3	*Rusa unicolor*	17,43	10,61	184,93
**THM245**	rp3	*Rusa unicolor*	16,59	8,81	146,16
**THM246**	rp3	*Rusa unicolor*	16,37	9,36	153,22
**THM247**	rp3	*Rusa unicolor*	18,27	10,31	188,36
**THM248**	rp3	*Rusa unicolor*	16,27	9,7	157,82
**THM249**	rp3	*Rusa unicolor*	19,48	9,63	187,59
**THM250**	rp3	*Rusa unicolor*	16,52	10,2	168,50
**THM251**	rp3	*Rusa unicolor*	17,53	9	157,77
**THM252**	rp3	*Rusa unicolor*	15,87	9,42	149,50
**THM252b**	rp3	*Rusa unicolor*	17,42	9,49	165,32
**THM253**	rp3	*Rusa unicolor*	17,28	10,55	182,30
**THM254**	rp3	*Rusa unicolor*	20,01	9,35	187,09
**THM255**	rp3	*Rusa unicolor*	16,68	8,36	139,44
**THM256**	rp3	*Rusa unicolor*	17,38	9,39	163,20
**THM198**	lp3	*Rusa unicolor*	16,68	8,25	137,61
**THM200**	lp3	*Rusa unicolor*	15,97	9,31	148,68
**THM201**	lp3	*Rusa unicolor*	17,26	8,98	154,99
**THM202**	lp3	*Rusa unicolor*	17,07	9,5	162,17
**THM203**	lp3	*Rusa unicolor*	16,99	10,19	173,13
**THM204**	lp3	*Rusa unicolor*	18,36	9,73	178,64
**THM205**	lp3	*Rusa unicolor*	17,67	10,38	183,41
**THM206**	lp3	*Rusa unicolor*	16,44	9,86	162,10
**THM207**	lp3	*Rusa unicolor*	18,33	9,21	168,82
**THM208**	lp3	*Rusa unicolor*	15,6	8,88	138,53
**THM209**	lp3	*Rusa unicolor*	16,53	10,11	167,12
**THM210**	lp3	*Rusa unicolor*	16,47	10,51	173,10
**THM211**	lp3	*Rusa unicolor*	17,52	10,51	184,14
**THM212**	lp3	*Rusa unicolor*	17,88	10,38	185,59
**THM213**	lp3	*Rusa unicolor*	15,44	8,99	138,81
**THM214**	lp3	*Rusa unicolor*	16,52	9,24	152,64
**CM495**	lp3	*Sus scrofa*	11,68	6,56	76,62
**CM687**	lp3	*Sus scrofa*	13,98	7,18	100,38
**CM688**	lp3	*Sus scrofa*	12,59	7,42	93,42
**CM689**	lp3	*Sus scrofa*	11,89	7,14	84,89
**CM690**	lp3	*Sus scrofa*	13	7,08	92,04
**CM791**	lp3	*Sus scrofa*	13,64	7,57	103,25
**CM891**	lp3	*Sus scrofa*	12,05	6,16	74,23
**CM892**	lp3	*Sus scrofa*	14,28	7,7	109,96
**CM460**	rp3	*Sus scrofa*	14,79	8,79	130,00
**CM788**	rp3	*Sus scrofa*	12,66	7,25	91,79
**CM789**	rp3	*Sus scrofa*	13,35	8,29	110,67

**CM790**	rp3	*Sus scrofa*	13,6	7,27	98,87
**CM792**	rp3	*Sus scrofa*	12,03	6,58	79,16
**THS492**	rp3	*Sus scrofa*	12,72	7,79	99,09
**THS461**	rp3	*Sus scrofa*	10,16	8,06	81,89
**THS500**	rp3	*Sus scrofa*	11,6	6,99	81,08
**THS501**	rp3	*Sus scrofa*	12,96	9,52	123,38
**THS673**	rp3	*Sus scrofa*	13,51	7,08	95,65
**THS703**	rp3	*Sus scrofa*	14,02	7,98	111,88
**THS708**	rp3	*Sus scrofa*	13,51	9,07	122,54
**THS709**	rp3	*Sus scrofa*	14,07	8,9	125,22
**THS710**	rp3	*Sus scrofa*	14,11	7,97	112,46
**THS676**	lp3	*Sus scrofa*	13,67	8,69	118,79
**THS702**	lp3	*Sus scrofa*	13,03	8,44	109,97
**THS704**	lp3	*Sus scrofa*	13,9	7,98	110,92
**NL209**	lp3	*Sus scrofa*	14,62	7,88	115,21
**NL213**	rp3	*Sus scrofa*	14,95	8,95	133,80
**NL219**	rp3	*Sus scrofa*	14,05	7,83	110,01
**NL220**	rp3	*Sus scrofa*	14,18	8,88	125,92
**DU866**	rp3	*Sus scrofa*	12,61	6,62	83,48
**DU867**	rp3	*Sus scrofa*	12,1	5,55	67,16
**DU868**	rp3	*Sus scrofa*	13,91	7,71	107,25
**DU869**	rp3	*Sus scrofa*	11,59	6,12	70,93
**DU883**	rp3	*Sus scrofa*	14,05	8,37	117,60
**DU884**	rp3	*Sus scrofa*	14,41	7,25	104,47
**DU886**	rp3	*Sus scrofa*	12,8	6,41	82,05
**DU891**	rp3	*Sus scrofa*	14,13	7,96	112,47
**DU1129**	rp3	*Sus scrofa*	12,38	5,94	73,54
**DU865**	lp3	*Sus scrofa*	12,5	6,6	82,50
**DU870**	lp3	*Sus scrofa*	12,88	6,83	87,97
**DU878**	lp3	*Sus scrofa*	13,53	6,25	84,56
**DU885**	lp3	*Sus scrofa*	12,63	7,01	88,54
**DU887**	lp3	*Sus scrofa*	12,25	6,33	77,54
**THM823**	rp3	*Sus scrofa*	12,8	6,47	82,82
**THM858**	rp3	*Sus scrofa*	14,16	8,02	113,56
**THM859**	rp3	*Sus scrofa*	13,37	7,76	103,75
**THM860**	rp3	*Sus scrofa*	13,21	6,58	86,92
**THM862**	rp3	*Sus scrofa*	13,56	8,65	117,29
**THM864**	rp3	*Sus scrofa*	12,79	7,84	100,27
**THM865**	rp3	*Sus scrofa*	11,7	5,86	68,56
**THM866**	rp3	*Sus scrofa*	11,58	6,12	70,87
**THM879b**	rp3	*Sus scrofa*	15,01	7,89	118,43
**THM880**	rp3	*Sus scrofa*	11,5	5,73	65,90
**THM867**	lp3	*Sus scrofa*	13,05	6,39	83,39
**THM868**	lp3	*Sus scrofa*	11,5	5,55	63,83
**THM873**	lp3	*Sus scrofa*	13,19	8,17	107,76
**THM874**	lp3	*Sus scrofa*	14,74	7,7	113,50

**THM875**	lp3	*Sus scrofa*	13,07	8,03	104,95
**THM876**	lp3	*Sus scrofa*	13,32	7,51	100,03
**THM877**	lp3	*Sus scrofa*	13,2	6,83	90,16
**THM878**	lp3	*Sus scrofa*	12,63	7,52	94,98
**CM473**	lm3	*Macaca* sp.	11,28	6,94	78,28
**CM613**	lm3	*Macaca* sp.	9,39	6,17	57,93
**THS71**	rm3	*Macaca* sp.	12,68	7,84	99,41
**THS73**	lm3	*Macaca* sp.	11,65	7,01	81,67
**THS74**	lm3	*Macaca* sp.	11,76	7,64	89,85
**THS75**	lm3	*Macaca* sp.	13,73	9,01	123,71
**THS76**	lm3	*Macaca* sp.	12,31	7,85	96,63
**THS855**	lm3	*Macaca* sp.	11,53	7,76	89,47
**THS856**	rm3	*Macaca* sp.	10,17	7,02	71,39
**NL314**	rm3	*Macaca* sp.	11,8	7,2	84,96
**NL323**	rm3	*Macaca* sp.	13	8,2	106,6
**DU186**	lm3	*Macaca* sp.	10,23	7,55	77,24
**DU187**	lm3	*Macaca* sp.	12,3	7,32	90,04
**DU188**	lm3	*Macaca* sp.	10,71	6,86	73,47
**DU189**	lm3	*Macaca* sp.	10,98	7,44	81,69
**DU190**	rm3	*Macaca* sp.	11,45	7,09	81,18
**DU191**	rm3	*Macaca* sp.	10,38	6,59	68,40
**DU192**	rm3	*Macaca* sp.	10,37	6,46	66,99
**DU193**	lm3	*Macaca* sp.	10,42	6,44	67,10
**DU194**	lm3	*Macaca* sp.	11,27	7,6	85,65
**DU195d**	rm3	*Macaca* sp.	12,32	7,56	93,14
**DU196**	rm3	*Macaca* sp.	12,41	7,54	93,57
**DU1130**	rm3	*Macaca* sp.	12,18	7,89	96,10
**DU1160**	lm3	*Macaca* sp.	11,68	6,92	80,83
**DU1161**	rm3	*Macaca* sp.	11,55	6,91	79,81
**DU1162**	rm3	*Macaca* sp.	11,75	7,28	85,54
**THM1193**	rm3	*Macaca* sp.	11,55	7,54	87,08
